# Supplementary material for: The effects of high-heeled shoes on gait parameters in healthy adult women
Source: PLoS One. 2025 Jul 3;20(7):e0327250. doi: 10.1371/journal.pone.0327250 (PMC12225787; doi:10.1371/journal.pone.0327250)
Supplement: Attachment — Submitted filename: PONE-D-24-32099_reviewer_reviewers comments.pdf [file pone.0327250.s001.pdf]

PLOS ONE

Effects of high-heeled shoes on gait parameters in healthy adult women

--Manuscript Draft--

**Manuscript Number:**	PONE-D-24-32099
**Article Type:**	Research Article
**Full Title:**	Effects of high-heeled shoes on gait parameters in healthy adult women
**Short Title:**	Effects of high-heeled shoes on gait parameters
**Corresponding Author:**	Tomoki Aoyama, MD, PhD<br>Kyoto University: Kyoto Daigaku<br>Kyoto City, Kyoto JAPAN
**Keywords:**	High-heeled shoes; shoes; walking; Women; IMS
**Abstract:**	Walking is an essential aspect of daily life. Research has shown that footwear type significantly affects walking patterns; for example, high-heeled shoes (most commonly worn by women) increase the risk of knee osteoarthritis and falls. However, most studies assessing this influence have used treadmill walking or unfamiliar footwear, which may not reflect daily walking patterns. This study therefore investigated the impact of high-heeled shoes on walking parameters in healthy adult women using in-shoe motion sensors. Seventeen healthy adult women without pain during walking participated in this study. All participants walked for 6 minutes along a 30-meter corridor wearing high-heeled shoes and sneakers. Walking data were recorded using an in-shoe motion sensor system every 2 minutes. The average of three valid consecutive steps was calculated automatically. Statistical analysis was performed to compare the mean walking parameters between the high-heel and sneaker groups. The high-heel group showed significantly lower foot clearance, stride length, peak plantar angle in dorsiflexion, and peak plantar angle in plantarflexion, as well as a significantly higher toe-out angle in spatial parameters. Spatiotemporal parameters revealed significantly lower walking speed and maximum swing phase speed in the high-heel group. No significant inter-group differences were observed in temporal parameters. This study provides insights into possible methods for injury prevention in healthy adult women walking in high-heeled shoes, and thus has the potential to contribute to future research on daily walking evaluations. Future studies should include diverse participants and measurement devices that assess hip and knee joint movements and neurological responses to comprehensively evaluate the impact of high-heeled shoes. Additionally, understanding the biomechanical and neurological effects of different footwear can guide the development of safer shoe designs for daily use.
**Order of Authors:**	Kyoma Tanigawa
	Hiroki Shimizu
	Anuradhi Bandara
	Misa Toyota
	Shota Suzuki
	Momoko Nagai-Tanima
	Tomoki Aoyama
**Additional Information:**	
**Question**	**Response**
**Financial Disclosure**<br><br>Enter a financial disclosure statement that describes the sources of funding for the work included in this submission. Review	The author(s) received no specific funding for this work.

the submission guidelines for detailed requirements. View published research articles from *PLOS ONE* for specific examples.<br><br>This statement is required for submission and **will appear in the published article** if the submission is accepted. Please make sure it is accurate.<br><br>**Funded studies**<br>Enter a statement with the following details:<br>• Initials of the authors who received each award<br>• Grant numbers awarded to each author<br>• The full name of each funder<br>• URL of each funder website<br>• Did the sponsors or funders play any role in the study design, data collection and analysis, decision to publish, or preparation of the manuscript?<br><br>Did you receive funding for this work?	
**Competing Interests**<br><br>Use the instructions below to enter a competing interest statement for this submission. On behalf of all authors, disclose any competing interests that could be perceived to bias this work—acknowledging all financial support and any other relevant financial or non-financial competing interests.<br><br>This statement is required for submission and **will appear in the published article** if the submission is accepted. Please make sure it is accurate and that any funding sources listed in your Funding Information later in the submission form are also declared in your Financial Disclosure statement.<br><br>View published research articles from *PLOS ONE* for specific examples.	The authors have declared that no competing interests exist.

Powered by Editorial Manager® and ProduXion Manager® from Aries Systems Corporation

**NO authors have competing interests**<br><br>Enter: *The authors have declared that no competing interests exist.*<br><br>**Authors with competing interests**<br><br>Enter competing interest details beginning with this statement:<br><br>*I have read the journal's policy and the authors of this manuscript have the following competing interests: [insert competing interests here]*<br><br>* typeset	
**Ethics Statement**<br><br>Enter an ethics statement for this submission. This statement is required if the study involved:<br><br>• Human participants<br>• Human specimens or tissue<br>• Vertebrate animals or cephalopods<br>• Vertebrate embryos or tissues<br>• Field research<br><br>Write "N/A" if the submission does not require an ethics statement.<br><br>General guidance is provided below. Consult the submission guidelines for detailed instructions. **Make sure that all information entered here is included in the Methods section of the manuscript.**	This study was approved by the Kyoto University Medical Ethics Committee (Approval number: R3664-3). Prior to study initiation, written informed consent was obtained from all participants in accordance with the ethical standards and guidelines.

Powered by Editorial Manager® and ProduXion Manager® from Aries Systems Corporation

**Format for specific study types**<br><br>**Human Subject Research (involving human participants and/or tissue)**<br>• Give the name of the institutional review board or ethics committee that approved the study<br>• Include the approval number and/or a statement indicating approval of this research<br>• Indicate the form of consent obtained (written/oral) or the reason that consent was not obtained (e.g. the data were analyzed anonymously)<br><br>**Animal Research (involving vertebrate animals, embryos or tissues)**<br>• Provide the name of the Institutional Animal Care and Use Committee (IACUC) or other relevant ethics board that reviewed the study protocol, and indicate whether they approved this research or granted a formal waiver of ethical approval<br>• Include an approval number if one was obtained<br>• If the study involved *non-human primates*, add *additional details* about animal welfare and steps taken to ameliorate suffering<br>• If anesthesia, euthanasia, or any kind of animal sacrifice is part of the study, include briefly which substances and/or methods were applied<br><br>**Field Research**<br><br>Include the following details if this study involves the collection of plant, animal, or other materials from a natural setting:<br>• Field permit number<br>• Name of the institution or relevant body that granted permission	
**Data Availability**<br><br>Authors are required to make all data underlying the findings described fully available, without restriction, and from the time of publication. PLOS allows rare exceptions to address legal and ethical concerns. See the PLOS Data Policy and FAQ for detailed information.	No - some restrictions will apply

A Data Availability Statement describing where the data can be found is required at submission. Your answers to this question constitute the Data Availability Statement and **will be published in the article**, if accepted.<br><br>**Important:** Stating 'data available on request from the author' is not sufficient. If your data are only available upon request, select 'No' for the first question and explain your exceptional situation in the text box.<br><br>Do the authors confirm that all data underlying the findings described in their manuscript are fully available without restriction?	
**Describe where the data may be found in full sentences. If you are copying our sample text, replace any instances of XXX with the appropriate details.**<br><br>• If the data are **held or will be held in a public repository**, include URLs, accession numbers or DOIs. If this information will only be available after acceptance, indicate this by ticking the box below. For example: *All XXX files are available from the XXX database (accession number(s) XXX, XXX.*).<br>• If the data are all contained **within the manuscript and/or Supporting Information files**, enter the following: *All relevant data are within the manuscript and its Supporting Information files.*<br>• If neither of these applies but you are able to provide **details of access elsewhere**, with or without limitations, please do so. For example:<br><br>*Data cannot be shared publicly because of [XXX]. Data are available from the XXX Institutional Data Access / Ethics Committee (contact via XXX) for researchers who meet the criteria for access to confidential data.*<br><br>*The data underlying the results presented in the study are available from (include the name of the third party*	The dataset used in this study is available from the corresponding author upon request.

and contact information or URL).<br>• This text is appropriate if the data are owned by a third party and authors do not have permission to share the data.<br><br>* typeset	
Additional data availability information:	

Click here to access/download;Manuscript;Manuscript_Kyoma_Tanigawa.do

Effects of high-heeled shoes on gait parameters in healthy adult women

Short Title: Effects of high-heeled shoes on gait parameters

Kyoma Tanigawa, Hiroki Shimizu, Anuradhi Bandara, Misa Toyota, Shota Suzuki, Momoko Nagai-Tanima, Tomoki Aoyama*

Department of Physical Therapy, Human Health Sciences, Graduate School of Medicine, Kyoto University, Kyoto, Japan

*Corresponding author

E-mail: aoyama.tomoki.4e@kyoto-u.ac.jp (TA)

Abstract

Walking is an essential aspect of daily life. Research has shown that footwear type significantly affects walking patterns; for example, high-heeled shoes (most commonly worn by women) increase the risk of knee osteoarthritis and falls. However, most studies assessing this influence have used treadmill walking or unfamiliar footwear, which may not reflect daily walking patterns. This study therefore investigated the impact of high-heeled shoes on walking parameters in healthy adult women using in-shoe motion sensors. Seventeen healthy adult women without pain during walking participated in this study. All participants walked for 6 minutes along a 30-meter corridor wearing high-heeled shoes and sneakers. Walking data were recorded using an in-shoe motion sensor system every 2 minutes. The average of three valid consecutive steps was calculated automatically. Statistical analysis was performed to compare the mean walking parameters between the high-heel and sneaker groups. The high-heel group showed significantly lower foot clearance, stride length, peak plantar angle in dorsiflexion, and peak plantar angle in plantarflexion, as well as a significantly higher toe-out angle in spatial parameters. Spatiotemporal parameters revealed significantly lower walking speed and maximum swing phase speed in the high-heel group. No significant inter-group differences were observed in temporal parameters. This study provides insights into possible methods for injury prevention in healthy adult women walking in high-heeled shoes, and thus has the potential to contribute to future research on daily walking evaluations. Future studies should include diverse participants and measurement devices that assess hip and knee joint movements and neurological responses to comprehensively evaluate the impact of high-

heeled shoes. Additionally, understanding the biomechanical and neurological effects of different footwear can guide the development of safer shoe designs for daily use.

Introduction

Walking is a crucial means of transporting the body safely and efficiently [1], which further exerts beneficial effects on both physical and mental health, enhances quality of life (QOL), and plays a significant role in the prevention and treatment of various diseases [2]. As such, walking is indispensable in daily life. However, walking is known to be influenced by various external factors [3], including footwear. Shoes, which come in various types, have multiple functions, including protecting the feet from external environments, assisting walking functions, and adapting to foot deformities. These functions are related to the occurrence of various conditions, such as falls, osteoarthritis, and back pain [4]. Numerous studies have previously investigated the impact of shoes on walking parameters [5,6]. For example, one study by Shakoor et al. comparing walking barefoot and wearing athletic shoes found that the latter condition was associated with decreased step length, increased number of steps, and reduced joint angles of the lower limbs [5]. Thus, research on how shoes affect walking is of significant importance [7].

One type of shoe that warrants attention is high-heeled shoes, which are defined as shoes with heels higher than the forefoot, narrow toe boxes, and curved soles [8]. High-heeled shoes have been widely

worn by women worldwide for centuries, with an estimated 37% to 69% of women wearing them regularly and for an average of 1 to 8 hours per day [8]. However, despite their regular use, high-heeled shoes are associated with conditions such as osteoarthritis and falls [9]. The incidence of such injuries doubled from 7.1% to 14.2% between 2002 and 2012 [10]. As such, it is crucial to investigate the impact of high-heeled shoes on the walking parameters of healthy adult women [7]. Indeed, several prior studies have already examined this impact. For example, it has been shown that wearing high-heeled shoes does not change temporal walking parameters, such as stance time and cadence [11]. Additionally, a study by Barkema et al. noted that a higher the heel height was associated with a greater the maximum knee adduction moment, indicating a relationship between heel height and knee osteoarthritis [9].

Although the impact of high-heeled shoes on walking parameters has been investigated, most evaluations were conducted using treadmill walking at constant speeds. Additionally, participants often used prescribed shoes they were unaccustomed to, meaning that the experimental conditions were distinct from those of daily life [12]. Such differences could alter individual walking styles in response to footwear, as such, these studies may not accurately reflect everyday walking [11]. Inertial Measurement Units (IMUs), which are used to estimate three-dimensional movement from angular velocity and acceleration [13,14], represent a more suitable evaluation method. Due to their low cost, small size, and ease of use, IMUs have been widely used to evaluate daily life walking in recent years

[15]. Through the use of an In-Shoe Motion Sensor system (IMS), which measures the acceleration and angular velocity of the feet using IMUs and calculates walking parameters, it is possible to obtain walking data which is a more accurate reflection of daily life than conventional studies [16].

Thus, this study aimed to elucidate the impact of high-heeled shoes on the walking parameters of healthy adult women using IMS.

Materials and methods

Study design

This was an intervention study.

Participants

The participants in this study were healthy adult women enrolled at Kyoto University. The inclusion criteria were: age 18 years or older, and able walk for at least 6 minutes without rest. The exclusion criteria were: neurological or orthopedic conditions that could affect walking function. The purpose of the study was thoroughly explained to the participants, and their consent was obtained prior to measurement. Additionally, the recruitment was conducted from July 1 to 31, 2023.

Ethics statement

This study was approved by the Kyoto University Medical Ethics Committee (Approval number: R3664-3). Prior to study initiation, written informed consent was obtained from all participants in accordance with the ethical standards and guidelines.

Equipment

The In-shoe Motion Sensor System (IMS, A-RROWG, NEC Corporation, Tokyo, Japan) was used for all measurements. The IMS is a device used for gait analysis which comprises several key components, including an ARM Cortex-M4F MCU (nRF52832 by Nordic Semiconductor). This component features a 64 MHz CPU, 64 KB of RAM, and 512 KB of ROM. Additionally, it includes a Bosch IMU (BMI160) for precise motion tracking, an ABLIC EEPROM (S-24C32C, 32K-bit) for storage, and an Epson real-time clock (RX8130CE). Measurements were conducted from 6:00 AM to 10:00 PM at 2-minute intervals. The IMU is active for the first minute of the two-minute period, but goes to sleep as soon as the measurement is complete. During the first minute, vibration detection and stable walking discrimination are performed, and if it is determined that walking has started, walking parameters are measured for 3 steps in the next 9 seconds. The three steps can be non-continuous, and if you successfully measure three steps in 9 seconds, the average value will be recorded. If it is less than 2 steps, it will not be recorded as a measurement failure. Even if three or more measurements are successful, the first three measurements will be valid. Also, if the above

sequence is performed up to three times in one minute and all three times fail, the measurement in that time slot will fail and no data will be recorded.

The following 16 parameters were calculated: Stride length, Maximum (peak) dorsiflexion angle, Maximum (peak) plantar flexion angle, Foot clearance, Foot height, Circumduction, Toe-in/out angle, Roll angle of heel contact, Roll angle of toe-off, Walking speed, Peak swing angular velocity, Maximum speed during swinging phase, Stance time, Swing time, Pushing time, Cadence. Table 1 categorizes the definitions of each parameter into spatial, spatiotemporal, and temporal parameters, as disclosed by NEC Corporation. These parameters have either been validated for accuracy in previous studies or calculated from validated parameters [17-19]. The participants' own daily-worn sneakers were used for comparison with high-heeled shoes. The definition of each type of shoe was based on the "model number tracked to the purchase source, with the manufacturing company selling the shoes as high heels or sneakers." If the model number cannot be traced, high heels are defined as "shoes with a flat forefoot and a heel higher than the forefoot, without an ankle strap," and sneakers are defined as "shoes with rubber soles."

Table 1: Definitions of Each Parameter

Classification	Parameters	Definition
**Spatial parameters**	Stride length (cm)	Distance from the starting point to the end point of the foot trajectory

	Maximum (peak) dorsiflexion angle (°)	Peak foot-sole angle in dorsiflexion
	Maximum (peak) plantar flexion angle (°)	Peak foot-sole angle in plantarflexion
	Foot clearance (cm)	Maximum heel height of the foot trajectory
	Foot height (cm)	Maximum vertical height of the foot trajectory
	Circumduction (cm)	Displacement in the medial–lateral direction during the swing phase
	Toe-in/out angle (°)	Average adduction/abduction angle during the swing phase
	Roll angle of heel contact (°)	Plantar roll angle when the heel touches the ground
	Roll angle of toe-off (°)	Plantar roll angle when the toe leaves the ground
**Spatiotemporal parameters**	Walking speed (km/h)	Stride length divided by stride time

	Peak swing angular velocity (° /s)	Peak angular velocity of the foot during the swing phase
	Maximum speed during swinging phase (km/h)	Maximum forward speed of the swinging leg during the swinging phase
**Temporal parameters**	Stance time (s)	The amount of time that the foot is on the ground during the gait cycle.
	Swing time (s)	The amount of time that the foot is off the ground during the gait cycle.
	Pushing time (s)	The time between heel leaving the ground and toe leaving the ground.
	Cadence (steps/min)	The number of steps per minute

Procedure

All measurements were conducted in a 30-meter corridor at the Faculty of Human Health Sciences building of Kyoto University. Initially, we verified whether the shoes worn by participants met the aforementioned criteria. Subsequently, we collected data on the participants' age, height, weight, foot size, heel height of the high-heeled shoes, and weight of each shoe. Participants walked 5 meters with

insoles equipped with IMS inserted into their shoes to ensure they experienced no discomfort during walking. The measurer stood at the center of the path during measurements to confirm that participants were walking normally and safely. Additionally, the order of wearing high-heeled shoes and sneakers was determined using the random function of Excel. All participants were instructed to walk at their usual pace continually for 6 minutes, starting from one end of the corridor, and turning upon reaching the other end.

Data analysis

Statistical analysis was performed using JMP PRO16 (SAS Institute). Shapiro-Wilk tests were conducted to assess the normality of distributions for comparisons between the heel group and sneaker group. Normally and non-normally distributed data were compared using paired t-tests and Wilcoxon signed-rank tests, respectively. The statistical significance level for this study was set at less than 5% ($P < 0.05$).

Results

Physical characteristics

This study included 17 participants, with no dropouts or adverse events reported (Table 2). The mean values were as follows: age, 23.4 years; height, 161.78 cm; weight, 53.46 kg; BMI, 20.38; shoe

size, 24.15 cm; weight of high-heeled shoes, 201.76 g; weight of sneakers, 265.00 g; heel height of high-heeled shoes, 4.93 cm.

Table 2: Basic information of the study participants

N=17	Mean±SD
Age(years)	23.4±1.23
Height(cm)	161.78±6.14
Weight(kg)	53.46±5.61
BMI(kg/m^2)	20.38±1.09
Size of shoes(cm)	24.15±1.01
Weight of heel shoes(g)	201.76±37.12
Weight of sneakers(g)	265.00±59.48
Heel height of heel shoes(cm)	4.93±1.17

SD, standard deviation; BMI, body mass index

Comparison of walking parameters between high-heeled and sneaker shoes

Table 3 shows the results of comparisons between the high-heel group (HG) and the sneaker group (SG). Analysis of spatial parameters revealed that the high-heeled group had a significantly shorter stride length, lower peak dorsiflexion angle of the foot, lower peak plantar flexion angle of the foot, and significantly higher toe angle. Regarding spatiotemporal parameters, the high-heeled group exhibited significantly lower walking speed and swing phase maximum velocity. There were no significant differences in temporal parameters between the two groups.

Table 3: Comparisons of the mean values of each parameter in each category. (mean±SD).

Classification	Parameters	HG	SG	P-value
**Spatial parameters**	Stride length(cm)	130.42±18.14	143.88±16.09	0.0006*
	Maximum (peak) dorsiflexion angle (°)	24.21±5.25	29.88±5.13	0.0017*
	Maximum (peak) plantar flexion angle (°)	62.33±11.15	78.31±6.49	<0.0001*
	Foot clearance (cm)	21.17±3.31	24.05±2.15	0.0005*
	Foot height (cm)	13.33±2.40	15.70±1.76	0.0023*
	Circumduction (cm)	4.10±1.58	3.72±2.01	0.5397
	Toe-in/out angle (°)	12.05±8.82	10.14±7.97	0.0476*

	Roll angle of heel contact (°)	5.21±4.86	3.71±5.70	0.2343
	Roll angle of toe-off (°)	-2.05±6.17	-1.36±4.52	0.5178
**Spatiotemporal parameters**	Walking speed (km/h)	4.61±0.68	5.08±0.66	0.0005*
	Peak swing angular velocity (°/s)	531.64±95.96	565.64±75.54	0.0932
	Maximum speed during swinging phase (km/h)	15.15±1.65	15.97±1.59	0.0085*
**Temporal parameters**	Stance time(s)	0.62±0.05	0.62±0.04	0.8271
	Swing time(s)	0.41±0.04	0.40±0.01	0.3225
	Pushing time (s)	0.19±0.03	0.20±0.02	0.6805
	Cadence (steps/min)	117.22±6.41	116.92±5.69	0.8030

SD, standard deviation. *$p<0.05$.

Discussion

The aim of this study was to investigate the effects of high-heeled shoes on walking parameters in healthy adult women using IMS. Overall, our results showed that, in terms of spatial parameters, high-heeled walking was associated with significantly shorter stride length, lower maximum

dorsiflexion angle, lower maximum plantar flexion angle, and significantly higher toe-out angle compared to the sneaker group. Additionally, in regards to spatiotemporal parameters, high-heeled walking was associated with significantly lower walking and maximum speeds during the swinging phase. However, no significant differences were observed between the groups in terms of temporal parameters.

Previous studies have shown that wearing high-heeled shoes can results in shortening of the gastrocnemius muscle and Achilles tendon due to the proximity of ground reaction forces to the ankle joint as well as the curved shape of the sole, which causes the ankle joint to adopt a plantarflexed position [11]. Consequently, it has been suggested that the ankle plantarflexion moment decreases, indicating weaker propulsive force from the foot during high-heeled shoe use compared to sneakers or barefoot conditions [20]. Similarly, in the present study, the high-heeled group exhibited significantly lower maximum walking speed, maximum speed during swinging phase, and stride length, possibly for similar reasons.

Similarly, foot clearance and foot height were found to be significantly lower in the high-heeled group. Previous studies comparing kinematics such as lower limb sagittal plane joint angles with varying heel heights during steady walking speeds have shown that hip flexion angles during the swing phase do not change with heel height, while knee flexion angles significantly decrease as heel height increases [21-23]. In the present study, it is likely that the high-heeled group exhibited

decreased knee flexion angles during the swing phase, resulting in relatively lower positions of the lower leg and decreased foot clearance. Indeed, prior research has shown that decreased foot clearance increases the risk of falling [24].

As such, the high-heeled group showed significantly lower stride length, peak dorsiflexion angle of the foot, peak plantar flexion angle of the foot, walking speed, and swing phase maximum velocity, in addition to a significantly higher toe angle during the swing phase. A higher toe angle indicates a larger ankle eversion angle. Studies investigating the influence of heel height on the anterior-posterior joint moments of the lower limb have shown that knee internal rotation moments increase as heel height increases, potentially increasing the load on the medial compartment of the knee [9,25]. Conversely, walking with toes pointing outward reduces knee internal rotation moments and decreases the load on the medial compartment [26]. In the present study, it was suggested that the high-heeled group adopted a walking strategy with toes pointing outward to reduce the load on the medial compartment of the knee. Additionally, as the knee joint movements during stance and toe direction were not measured in this study, further experiments are required.

In conclusion, while significant differences were observed in spatial/spatiotemporal parameters, similar to previous studies, we did not observe any differences in temporal parameters [27]. Previous studies have shown that cadence during inclined and level walking remains unchanged [28]. Furthermore, Alkjaer et al. demonstrated that walking in high heels requires the adaptation of a

different neural control system compared to normal walking [29]. In the present study, it has been suggested that the use of a similar neural control system can lead to changes in spatial/spatiotemporal parameters of the lower limb, without altering temporal parameters.

This study has two main limitations which should be mentioned. First, the study was limited to healthy adult women, which may restrict the generalizability of the findings to other demographic groups. Second, measurements of joint movements and neurological responses, aside from those of the ankle joints (such as hip and knee joints), were not conducted. Therefore, the impact of these factors on the results remains unclear. Future research should aim to expand participant demographics and utilize other devices capable of measuring movements of joints other than the ankle, as well as neurological responses, concurrent with IMS. This comprehensive approach is required to more thoroughly evaluate the effects of different types of footwear on walking.

Conclusions

Overall, this study investigated the effects of high-heeled shoes on walking parameters in healthy adult women using IMS. The results were as follows: In terms of spatial parameters, the high-heeled group showed significantly shorter stride length, lower maximum dorsiflexion angle, lower maximum plantar flexion angle, and significantly higher toe-out angle compared to the sneaker group. Additionally, in terms of spatiotemporal parameters, the high-heeled group exhibited significantly

lower walking and maximum speed during swinging phase. Moving forward, it will be essential to more comprehensively evaluate the impact of high-heeled shoes on daily walking using experimental devices that could measure hip and knee joint movements and neurological responses concurrently with IMS. Nevertheless, this study provides new insights into injury prevention related to high-heeled shoe use in healthy adult women and can contribute to future research evaluating everyday walking.

Acknowledgments

We would like to express our gratitude to all the participants, instructors, and laboratory members for their cooperation and valuable input throughout this study. We would also like to thank Editage (www.editage.com) for English editing.

References

1. Prince F, Corriveau H., Hebert R, Winter DA. Gait in elderly. Gait Posture. 1997;5(2): 128-135. doi: 10.1016/S0966-6362(97)01118-1
2. Bai X, Kim GS, Dev RDO, Talib O, Xiao W, Cai H. Effect of brisk walking on health-related physical fitness balance and life satisfaction among the elderly: A systematic review. Front Pub Health. 2022;9. doi: https://doi.org/10.3389/fpubh.2021.829367

3. Menant JC, Steele JR, Menz HB, Munro BJ, Lord SR. Effects of walking surfaces and footwear on temporo-spatial gait parameters in young and older people. Gait Posture. 2009;29(3):392-397. doi: https://doi.org/10.1016/j.gaitpost.2008.10.057
4. Barton CJ, Bonanno D, Menz HB. Development and evaluation of a tool for the assessment of footwear characteristics. J Foot Ankle Res. 2009;2(10). doi: 10.1186/1757-1146-2-10
5. Shakoor N, Block JA. Walking barefoot decreases loading on the lower extremity joints in knee osteoarthritis. Arthritis Rheum. 2006;54(9):2923-2927. doi: 10.1002/art.22123
6. Keenan GS, Franz JR, Dicharry J, Della Croce U, Casey Kerrigan D. Lower limb joint kinetics in walking: The role of industry recommended footwear. Gait Posture. 2011;33(3):350-355. doi: 10.1016/j.gaitpost.2010.09.019
7. Luximon Y, Cong Y, Luximon A, Zhang M. Effects of heel base size, walking speed, and slope angle on center of pressure trajectory and plantar pressure when wearing high-heeled shoes. Hum Mov Sci. 2015;41;307-319. doi: 10.1016/j.humov.2015.04.003
8. Zeng Z, Liu Y, Hu X, Li P, Wang L. Effects of high-heeled shoes on lower extremity biomechanics and balance in females: a systematic review and meta-analysis. BMC Public Health. 2023;23(726). doi: 10.1186/s12889-023-15641-8
9. Barkema DD, Derrick TR, Martin PE. Heel height affects lower extremity frontal plane joint moments during walking. Gait Posture. 2012;35(3):483-488. doi: 10.1016/j.gaitpost.2011.11.013

10. Xavier MJ, Lambert B, Jenkins GP, McGwin Jr G. Epidemiology of high-heel shoe injuries in U.S. women: 2002 to 2012. J Foot Ankle Surg. 2015;54(4):615-619. doi: 10.1053/j.jfas.2015.04.008

11. Cronin NJ. The effects of high heeled shoes on female gait: A review. J Electromyogr Kinesiol. 2014;24(2):258-263. doi:10.1016/j.jelekin.2014.01.004

12. Annoni I, Mapelli A, Sidequersky FV, Zago M, Sforza C. The effect of high-heeled shoes on overground gait kinematics in young healthy women. Sport Sci Health. 2014;10(2):149-157. doi: 10.1007/s11332-014-0191-z

13. Aminian K, Renzakhanlou K, De Andres E, Fritsch C, Leyvraz PF, Robert P. Temporal feature estimation during walking using miniature accelerometers: an analysis of gait improvement after hip arthroplasty. Med Biol Eng Comput. 1999;37(6):686-691. doi: 10.1007/BF02513368

14. Jasiewicz JM, Allum JHJ, Middleton JW, Barriskill A, Condie P, Purcell B, et al. Gait event detection using linear accelerometers or angular velocity transducers in able-bodied and spinal-cord injured individuals. Gait Posture. 2006;24(4):502-509. doi: https://doi.org/10.1016/j.gaitpost.2005.12.017

15. Anwary AR, Yu H, Vassallo M. Optimal foot location for placing wearable IMU sensors and automatic feature extraction for gait analysis. IEEE Sensors J. 2018;18(6):2555-2567. doi: 10.1109/JSEN.2017.2786587

16. Yamamoto A, Fujita K, Yamada E, Ibara T, Nihey F, Inai T, et al. Foot characteristics of the daily-life gait in postmenopausal females with distal radius fractures: a cross-sectional study. BMC Musculoskelet Disord. 2023;24(1):706. doi: 10.1186/s12891-023-06845-5
17. Fukushi K, Huang C, Wang Z, Kajitani H, Nihey F, Nakahara K. On-Line Algorithms of Stride-Parameter Estimation for in-Shoe Motion-Sensor System. IEEE Sensors Journal. 2022;22(10). doi: 10.1109/JSEN.2022.3164057
18. Huang C, Fukushi K, Wang Z, Nihey F, Kajitani H, Nakahara K. Method for Estimating Temporal Gait Parameters Concerning Bilateral Lower Limbs of Healthy Subjects Using a Single In-Shoe Motion Sensor through a Gait Event Detection Approach. Sensors (Basel). 2022;22(1). doi: 10.3390/s22010351
19. Huang C, Fukushi K, Wang Z, Nihey F, Kajitani H, Nakahara K. An algorithm for real time minimum toe clearance estimation from signal of in-shoe motion sensor. 2021 43rd Annual International Conference of the IEEE Engineering in Medicine & Biology Society (EMBC)2021. doi: 10.1109/EMBC46164.2021.9629875
20. Esenyel M, Walsh K, Walden JG, Gitter A. Kinetics of high-heeled gait. J Am Podiatr Med Assoc. 2003;93(1):27-32. doi: 10.7547/87507315-93-1-27

21. Snow RE, Williams KR. High heeled shoes: Their effect on center of mass position, posture, three-dimensional kinematics, rearfoot motion, and ground reaction forces. Arch Phys Med Rehab. 1994;75(5):568-576. doi: 10.1016/S0003-9993(21)01622-1

22. Opila KA. Kinematics of high-heeled gait. Arch Phys Med Rehab. 1990;71(5):304-309. doi: 10.7547/87507315-93-1-27

23. Gehlsen G, Braarz JS, Assmann N. Effects of heel height on knee rotation and gait. Human Movement Science. 1986;5(2):149-155. doi: https://doi.org/10.1016/0167-9457(86)90021-7

24. Dadashi F, Mariani B, Rochat S, Bula CJ, Santos-Eggiman, B, Aminian K. Gait and foot clearance parameters obtained using shoe-worn inertial sensors in a large-population sample of older adults. Sensors. 2014;14(1):443-457. doi: 10.3390/s140100443

25. Teichtahl AJ, Morrie ME, Wluka AE, Baker R, Wolfe R, Davis SR, et al. Foot rotation-a potential target to modify the knee adduction moment. J Sci Med Sport. 2006;9(1-2):67-71. doi: https://doi.org/10.1016/j.jsams.2006.03.011

26. Chang A, Hurwitz D, Dunlop D, Song J, Cahue S, Hayes K, et al. The relationship between toe-out angle during gait and progression of medial tibiofemoral osteoarthritis. Ann Rheum Dis. 2007;66(10):1271-1275. doi: 10.1136/ard.2006.062927

27. Adrian MJ, Karpovich PV. Foot instability during walking in shoes with high heels. Res Q Am Assoc Health Phys Educ Recreat. 1966;37(2):168–175. doi: 10.1080/10671188.1966.10613359

28. Dewolf AH, Ivanenko YP, Zelik KE, Lacquaniti F, Willems PA. Differential activation of lumbar and sacral motor pools during walking at different speeds and slopes. J Neurophysiol. 2019;122(2):872-887. doi: 10.1152/jn.00167.2019

29. Alkjaer T, Raffalt P, Petersen NC, Simonsen EB. Movement behavior of high-heeled walking: How does the nervous system control the ankle joint during an unstable walking condition? PLoS ONE. 2012;7(5):e37390. doi: https://doi.org/10.1371/journal.pone.0037390
