# Supplementary material for: The effects of high-heeled shoes on gait parameters in healthy adult women
Source: PLoS One. 2025 Jul 3;20(7):e0327250. doi: 10.1371/journal.pone.0327250 (PMC12225787; doi:10.1371/journal.pone.0327250)
Supplement: Attachment — Submitted filename: PONE-D-24-32099_R1_reviewer_reviewer comments.pdf [file pone.0327250.s003.pdf]

PLOS ONE

Effects of high-heeled shoes on gait parameters in healthy adult women

--Manuscript Draft--

**Manuscript Number:**	PONE-D-24-32099R1
**Article Type:**	Research Article
**Full Title:**	Effects of high-heeled shoes on gait parameters in healthy adult women
**Short Title:**	Effects of high-heeled shoes on gait parameters
**Corresponding Author:**	Tomoki Aoyama<br>Kyoto University: Kyoto Daigaku<br>JAPAN
**Keywords:**	High-heeled shoes; shoes; Walking; women; IMS
**Abstract:**	Walking is an essential aspect of daily life. Research has shown that footwear type significantly affects walking patterns; for example, high-heeled shoes (most commonly worn by women) increase the risk of knee osteoarthritis and falls. However, most studies assessing this influence have used treadmill walking or unfamiliar footwear, which may not reflect daily walking patterns. This study therefore investigated the impact of high-heeled shoes on walking parameters in healthy adult women using in-shoe motion sensors. Seventeen healthy adult women without pain during walking participated in this study. All participants walked for 6 minutes along a 30-meter corridor wearing high-heeled shoes and sneakers. Walking data were recorded using an in-shoe motion sensor system every 2 minutes. The average of three valid consecutive steps was calculated automatically. Statistical analysis was performed to compare the mean walking parameters between the high-heel and sneaker groups. The high-heel group showed significantly lower foot clearance, stride length, peak plantar angle in dorsiflexion, and peak plantar angle in plantarflexion, as well as a significantly higher toe-out angle in spatial parameters. Spatiotemporal parameters revealed significantly lower walking speed and maximum swing phase speed in the high-heel group. No significant inter-group differences were observed in temporal parameters. This study provides insights into possible methods for injury prevention in healthy adult women walking in high-heeled shoes, and thus has the potential to contribute to future research on daily walking evaluations. Future studies should include diverse participants and measurement devices that assess hip and knee joint movements and neurological responses to comprehensively evaluate the impact of high-heeled shoes. Additionally, understanding the biomechanical and neurological effects of different footwear can guide the development of safer shoe designs for daily use.
**Order of Authors:**	Kyoma Tanigawa
	Hiroki Shimizu
	Anuradhi Bandara
	Misa Toyota
	Shota Suzuki
	Momoko Nagai-Tanima
	Tomoki Aoyama
**Response to Reviewers:**	We are very grateful to the editors for providing such valuable, thoughtful, and thorough comments. The comments and questions raised have prompted us to consider our data from new and interesting angles, the culmination of which we feel has greatly strengthened the work. Thank you for your time.<br><br>Editor’s comments:<br><br>[Comment #1]<br>Please use the space provided to explain your answers to the questions above. You may also include additional comments for the author, including concerns about dual

Powered by Editorial Manager® and ProduXion Manager® from Aries Systems Corporation

publication, research ethics, or publication ethics. (Please upload your review as an attachment if it exceeds 20,000 characters)

[Author action #1]
Thank you for your detailed review and for providing the opportunity to clarify our responses. We have addressed each of your questions thoroughly in the space provided, as well as in the manuscript itself where appropriate. We are committed to maintaining high standards of research and publication ethics, and we confirm that this study adheres to all ethical guidelines, including informed consent from participants and approval from the relevant ethics committee. We appreciate your time and consideration in reviewing our work.

Reviewer #1:
[Comment #1]
The purpose of this quasiexperimental study was to compare walking temporal spatial parameters in women wearing sneakers and high heeled shoes. The authors provided sufficient background perhaps identifying a need for this project. The manuscript was well organized, although insufficient information in the methods section would not allow this research to be replicated. The results, discussion and conclusion related to the study's primary purpose; although the conclusion needs to be expanded a bit. There were some minor errors in word choice and tense. See pdf for specific comments.

[Author action #1]
Thank you very much for your valuable feedback. As you suggested, we have expanded the methods section and conclusion, providing more detail in the methods to enhance reproducibility. In the conclusion, we clarified the implications of the results, future directions, and the significance of this study. We have also corrected errors related to word choice and tense. We would appreciate it if you could review it at your convenience. Thank you for your time and consideration.
The changed text is highlighted in yellow as follows:
Participants
The participants in this study were healthy adult women enrolled at Kyoto University. The inclusion criteria were individuals aged 18 years and older who could walk for at least 6 minutes without rest. To minimize the influence of unfamiliarity with high-heeled shoes on the results, we selected participants with extensive experience wearing high heels in various settings, such as work, social events, shopping, or formal occasions, based on previous studies [14]. The exclusion criteria were individuals with neurological or orthopedic conditions that could affect walking function. The purpose of the study was thoroughly explained to the participants, and their consent was obtained prior to measurement. The sample size for this study was calculated using G Power 3.1 (Heinrich Heine University). Based on prior research [24], with an assumed effect size of 0.62, an alpha level of 0.05, and a statistical power of 0.8, the minimum required number of participants was calculated to be 15. To account for potential data loss or incomplete data, an additional five participants were included, setting the final target number of participants at 20.
Line numbers where the changed text appears: 127-136

Equipment
The IMS System (A-RROWG, NEC Corporation, Tokyo, Japan) was used for the measurements. The IMS is a device used for gait analysis and consists of key components including an ARM Cortex-M4F MCU (nRF52832 by Nordic Semiconductor). This component features a 64 MHz CPU, 64 KB of RAM, and 512 KB of ROM. Additionally, it includes a Bosch IMU (BMI160) for precise motion tracking, an ABLIC EEPROM (S-24C32C, 32K-bit) for storage, and an Epson real-time clock (RX8130CE). This device performs gait measurements at 2-minute intervals, capturing gait parameters on both sides. The system detects the gait cycle during flat walking, operates for 1 min, and then enters sleep mode to conserve energy. During this 1-minute period, it detects vibrations and identifies stable walking patterns, and upon detecting the start of a gait cycle, it measures the parameters for three steps within the following 9 s. These steps do not need to be consecutive; if the three steps are successfully measured within a specified time, the average value is recorded. If fewer than three steps were measured, the average value was not recorded. Additionally, this measurement process can be repeated up to three times within a 1-minute window; if all three attempts fail, the measurement for that period is considered

Powered by Editorial Manager® and ProduXion Manager® from Aries Systems Corporation

unsuccessful, and no data is recorded [25,26,27].
The following 16 parameters are calculated: Stride length, Maximum (peak) dorsiflexion angle, Maximum (peak) plantar flexion angle, Foot clearance, Foot height, Circumduction, Toe-in/out angle, Roll angle of heel contact, Roll angle of toe-off, Walking speed, Peak swing angular velocity, Maximum speed during swinging phase, Stance time, Swing time, Pushing time, and Cadence. Table 1 below, disclosed by NEC Corporation, categorizes the definitions of each parameter into spatial, spatiotemporal, and temporal parameters. These parameters were validated in previous studies for accuracy or were derived from validated parameters [25,26,27].
High-heeled shoes were defined as "footwear with a heel elevated higher than the forefoot, a narrow toe box, and a curved sole" [11], while sneakers were defined as "light footwear that has a top made of cloth and a bottom made of rubber."
Line numbers where the changed text appears: 149-176

Procedure
The measurements were conducted in a 30-meter corridor at the Faculty of Human Health Sciences building, Kyoto University. First, we verified whether the shoes worn by the participants met the aforementioned criteria. Subsequently, we measured the age (years), height (cm), body mass(kg), size of the shoes(cm), heel height (cm) of the high-heeled shoes, and weight mass (g) of each shoe. Prior to data collection, participants walked 5 m with insoles equipped with an IMS inserted into their shoes to ensure that they experienced no discomfort during walking. For data collection, Participants placed a smartphone, used for data recording, in either pocket of their pants, as there were no restrictions on which side to use. They were instructed to begin walking from one end of the corridor, turn around at the opposite end, and continue this back-and-forth walking for 6 min. The measurements were conducted twice: once in sneakers and once in high-heeled shoes, with the order of conditions determined using Excel's random function. Participants were given the option to take a break between trials upon request. The observer stood in the middle of the pathway during the test to ensure that the participants walked normally and without safety issues, providing updates on the remaining time every minute. If no data were collected, the 6-minute walking test was repeated.
Line numbers where the changed text appears: 179-198

Data analysis
Statistical analyses were performed using the JMP PRO16 software (SAS Institute). First, the Shapiro-Wilk test was conducted to determine whether each dataset followed a normal distribution. For data that were normally distributed, a paired t-test was used, because the same participants were measured under different conditions (high-heeled shoes and sneakers). For data that did not follow a normal distribution, the Wilcoxon signed-rank test, suitable for paired datasets, was applied. The group wearing high-heeled shoes was referred to as the "high-heeled group" (HG) and the group wearing sneakers as the "sneaker group" (SG). Data are presented as mean standard deviation ± standard deviation(SD). The effect size was evaluated using Cohen's d to assess the magnitude of the differences between groups. The statistical significance level for this study was set at less than 5%.
Line numbers where the changed text appears: 200-213

Conclusions
This study investigated the effects of high-heeled shoes on walking parameters in healthy adult women using the IMS. As a result, in terms of spatial parameters, the high-heeled group showed significantly shorter stride length, lower maximum dorsiflexion angle, lower maximum plantar flexion angle, and significantly higher toe-out angle compared to the sneaker group, with effect sizes ranging from moderate to high. Additionally, in spatiotemporal parameters, the high-heeled group exhibited significantly lower walking speed and maximum speed during the swinging phase, with large effect sizes. However, no significant differences were observed between the groups in temporal parameters.
These findings suggest that wearing high-heeled shoes has a substantial impact on gait stability and efficiency, potentially increasing the risk of falls and musculoskeletal strain. Future studies should include a more diverse population, such as older adults or individuals with joint disorders, and incorporate motion capture systems or wearable sensors that can measure lower limb joint dynamics. These additional studies would allow for a comprehensive understanding of how footwear affects not only the ankle

Powered by Editorial Manager® and ProduXion Manager® from Aries Systems Corporation

but also hip and knee joint movements across a wider population. Because this study collected gait data under conditions similar to those of daily life, it provides data suitable for practical applications and may contribute to future research focused on evaluating gait in everyday scenarios.
Line numbers where the changed text appears: 342-364

[Comment #2]
Line 57
you claim "several prior studies" but only cited two. I recommend providing 4-6 total references related to high-heel research but perhaps highlighting only the two
[Author action #2]
Thank you for your valuable feedback. We have added two more references related to high-heel research, bringing the total to four references. As per your suggestion, we have highlighted the two most relevant references.
Additional Previous Research:
12. Alkjaer T, Raffalt P, Petersen NC, Simonsen EB. Movement Behavior of High-Heeled Walking: How Does the Nervous System Control the Ankle Joint during an Unstable Walking Condition? PLoS ONE. 2012;7(5). doi: https://doi.org/10.1371/journal.pone.0037390
13. Falola JM, Koussihouèdé FEN, Falola SMD, Avossevou YG, Bio NI, Dansou HP. Effect of the Base of the Shoe Heel on Postural Stability During Walking in Women. American Journal of BioScience. 2015;3(5):167-170. doi: 10.11648/j.ajbio.20150305.11

[Comment #3]
Lione 101
if you successfully measure three steps in 9 seconds, the average value will be recorded.
this phrase is grammatically incorrect, ie, use of the phrase "if you"
[Author action #3]
Thank you for your valuable feedback. We have revised the sentence for clarity and grammatical accuracy. The changed text is highlighted in yellow as follows:
upon detecting the start of a gait cycle, it measures parameters for three steps within the following 9 s.
Line numbers where the changed text appears: 159

[Comment #4]
Line121
Please provide information about the data collection frequency and any calibration procedures related to the IMU.
Was there only one testing session?

[Author action #4]
Thank you for your valuable feedback. We have addressed your comment as follows:
Regarding calibration, the accuracy and validity of the calculated parameters are ensured based on prior studies. Additionally, we have emphasized in the manuscript that these parameters were validated in previous studies for accuracy or were derived from validated parameters [25,26,27], ensuring the reliability of the measurements.
Regarding the test session, the details of the procedure have been described in the “Procedure” section for further clarity.
We appreciate your time and consideration in reviewing our revisions and would be grateful if you could confirm the updated content at your convenience.
The changed text is highlighted in yellow as follows:
Equipment
The IMS System (A-RROWG, NEC Corporation, Tokyo, Japan) was used for the measurements. The IMS is a device used for gait analysis and consists of key components including an ARM Cortex-M4F MCU (nRF52832 by Nordic Semiconductor). This component features a 64 MHz CPU, 64 KB of RAM, and 512 KB of ROM. Additionally, it includes a Bosch IMU (BMI160) for precise motion tracking, an ABLIC EEPROM (S-24C32C, 32K-bit) for storage, and an Epson real-time clock (RX8130CE). This device performs gait measurements at 2-minute intervals, capturing gait parameters on both sides. The system detects the gait cycle during flat walking, operates for 1 min, and then enters sleep mode to conserve energy. During this 1-

Powered by Editorial Manager® and ProduXion Manager® from Aries Systems Corporation

minute period, it detects vibrations and identifies stable walking patterns, and upon detecting the start of a gait cycle, it measures the parameters for three steps within the following 9 s. These steps do not need to be consecutive; if the three steps are successfully measured within a specified time, the average value is recorded. If fewer than three steps were measured, the average value was not recorded. Additionally, this measurement process can be repeated up to three times within a 1-minute window; if all three attempts fail, the measurement for that period is considered unsuccessful, and no data is recorded [25,26,27].
The following 16 parameters are calculated: Stride length, Maximum (peak) dorsiflexion angle, Maximum (peak) plantar flexion angle, Foot clearance, Foot height, Circumduction, Toe-in/out angle, Roll angle of heel contact, Roll angle of toe-off, Walking speed, Peak swing angular velocity, Maximum speed during swinging phase, Stance time, Swing time, Pushing time, and Cadence. Table 1 below, disclosed by NEC Corporation, categorizes the definitions of each parameter into spatial, spatiotemporal, and temporal parameters. These parameters were validated in previous studies for accuracy or were derived from validated parameters [25,26,27].
Line numbers where the changed text appears: 149-176

(Additional references)

25. Fukushi K, Huang C, Wang Z, Kajitani H, Nihey F, Nakahara K. On-line algorithms of stride-parameter estimation for in-shoe motion-sensor system. IEEE Sensors Journal. 2022;22:9636–9648. doi:https://doi.org/10.1109/JSEN.2022.3164057
26. Huang C, Fukushi K, Wang Z, Nihey F, Kajitani H, Nakahara K. An algorithm for real time minimum toe clearance estimation from signal of in-shoe motion sensor. Annual International Conference of the IEEE Engineering in Medicine and Biology Society. 2021: 6775–6778. doi:https://doi.org/10.1109/EMBC46164.2021.9629875
27. Huang C, Fukushi K, Wang Z, Nihey F, Kajitani H, Nakahara K. Method for estimating temporal gait parameters concerning bilateral lower limbs of healthy subjects using a single in-shoe motion sensor through a gait event detection approach. Sensors (Basel). 2022;22(1):351. doi:https://doi.org/10.3390/S22010351

[Comment #5]
Line123 Line142 Table 2
weight
[Author action #5]
Thank you for your valuable feedback. We have revised the words “weight” to “mass” for improved accuracy.

[Comment #6]
Line142
should be mass since units were grams
[Author action #6]
Thank you for your valuable feedback. We have revised the words “weight” to “mass” for improved accuracy.

[Comment #7]
Discussion
please briefly also state the problem you identified and the rationale/need for this study

[Author action #7]
Thank you very much for your valuable feedback. As you suggested, I have clarified the theoretical rationale and need for this study in the Discussion, especially in comparison with prior studies. In doing so, we re-evaluated the novelty of this study, which we believe primarily consists of the following three aspects:
1.Measurement under conditions close to daily life: Unlike other studies that use prescribed footwear, our study measured participants in their habitual shoes, collecting data that more accurately represents natural, everyday walking styles. Furthermore, many previous studies have conducted measurements on treadmills, which differ significantly from actual walking conditions. By performing measurements in real-life conditions, our study is able to provide practical insights that more realistically reflect individual walking patterns.
2.High-precision data collection of daily movement using IMS: Instead of traditional

motion capture systems or treadmill-based experiments, we utilized the In-Shoe Motion Sensor (IMS) system, allowing measurements in an environment closer to everyday life. This enables a realistic evaluation of the impact of wearing high heels in typical environments outside the laboratory, offering valuable feedback applicable to real-world conditions.
3.Practical application perspective: The data collection method using IMS serves as foundational data that can be leveraged to evaluate fall risks in everyday walking and provide feedback for designing comfortable and safe footwear. This data can be applied in the future to develop more refined footwear suited for everyday walking and create effective gait training programs, adding further value to this study.
Anyway, the changed text is highlighted in yellow as follows:
(The introduction for Discussion)
This study aimed to evaluate the effect of high-heeled shoes on gait parameters in healthy adult women using an IMS system. In previous studies, participants often wore unfamiliar shoes and measurements were commonly conducted on a treadmill at a constant speed, which may not accurately reflect natural gait patterns in real-life settings. In contrast, this study utilized a compact and user-friendly IMS to collect gait data while participants wore their own familiar shoes. This approach allowed for accurate collection in a manner closer to daily life conditions, providing foundational data for assessing the effects of footwear under more realistic circumstances.
Line numbers where the changed text appears: 238-246

(Conclusion)
Because this study collected gait data under conditions similar to those of daily life, it provides data suitable for practical applications and may contribute to future research focused on evaluating gait in everyday scenarios.
Line numbers where the changed text appears: 355-364

[Comment #8]
Line 169
can results
[Author action #8]
Thank you for pointing out the grammatical issue. We have revised the sentence to ensure it adheres to proper grammatical standards.

[Comment #9]
Line187 “A higher toe angle indicates a larger ankle eversion angle.”
this is not intuitive and so it needs some explanation along with citing a reference
[Author action #9]
Thank you for your comment. I made a correction regarding the angle described. As mentioned in Table 1, the term “toe angle” in the manuscript should refer to abduction, not eversion. We're sorry for my mistake. We have revised the text to ensure the correct terminology is used.
The changed text is highlighted in yellow as follows:
A higher toe angle indicates a larger ankle abduction angle.
Line numbers where the changed text appears: 307

[Comment #10]
Conclusion
a conclusion needs to be more than a summary of major findings; please expand and consider expressing possible implications of your findings
[Author action #10]
Thank you very much for your valuable feedback. As you pointed out, I have expanded the conclusion to include the potential implications of the results. Additionally, I have revised other sections based on your suggestions to make the content more appropriate. I would appreciate it if you could review it at your convenience.
The changed text is highlighted in yellow as follows:
These findings suggest that wearing high-heeled shoes has a substantial impact on gait stability and efficiency, potentially increasing the risk of falls and musculoskeletal strain.
Line numbers where the changed text appears: 355-357

Reviewer #2:
[Comment #1]

Powered by Editorial Manager® and ProduXion Manager® from Aries Systems Corporation

My opinions are as follows:
- There are many studies in the literature on this subject (There is a systematic review and meta-analysis). Please clearly state the difference between your study and other studies. Similar biomechanical parameters to the ones you evaluated have been given in many studies. This study does not yield any different results than those studies, nor does it provide information that would help form a different hypothesis. I do not think that the study will add much to the literature.
Zeng et al. 2023. Effects of high-heeled shoes on lower extremity biomechanics and balance in females: a systematic review and meta-analysis.
[Author action #1]
Thank you very much for your valuable feedback. I apologize for the similarity to my response to Reviewer 1, but as you correctly pointed out, I have revised the Discussion section to clarify the novelty of this study in comparison with previous research. Additionally, I aimed to make this study as meaningful as possible for future research by providing a more detailed description of each parameter.
In revising, I re-evaluated the unique aspects of this study and identified three main points that highlight its novelty. The Discussion was revised based on these three points:
1.Measurement Under Real-Life Conditions: Unlike other studies that often employ standardized footwear, this study measured participants in the shoes they habitually wear, thus capturing data that closely reflects real-life walking patterns. Many previous studies have conducted evaluations using treadmill walking, which does not fully represent natural gait. By conducting measurements under conditions equivalent to daily life, this study provides a more realistic reflection of individual walking patterns and contributes valuable practical insights.
2.High-Precision Data Collection of Everyday Movements Using IMS: Replacing conventional motion capture systems and treadmill-based experiments, this study utilized an In-Shoe Motion Sensor (IMS) system, allowing for assessments in settings closer to everyday environments. This approach enables a more realistic evaluation of the effects of high-heeled shoes outside of laboratory conditions, providing insights that are applicable to real-life contexts.
3.Practical Applicability: The IMS-based data collection method offers foundational data that can be used to assess fall risks during daily walking and to inform the design of safer and more comfortable footwear. This data adds value by potentially contributing to the development of refined shoe designs suited for daily walking and to more effective gait training methods in the future.
Additionally, the changed text is highlighted in yellow as follows:
(The Introduction)
Research on the effects of high-heeled shoes on gait parameters has significantly progressed. However, many evaluations have been conducted using a treadmill with participants walking at a constant speed [14]. Treadmill walking occurs under flat, steady-speed conditions, which differs from everyday walking environments [15]. For example, because a treadmill maintains a fixed speed, participants cannot walk at their natural pace, potentially suppressing their typical gait patterns [16,17]. Additionally, on a treadmill, individuals often need to adjust their posture and gait mechanics to match the treadmill's motion in order to maintain visual and balance cues, which is known to influence gait biomechanics [15]. In certain studies, participants wore unfamiliar shoes that may not accurately reflect their usual gait patterns in daily life [11,14]. Shoes that are unfamiliar or uncomfortable for an individual often do not fit well with the person's foot shape or gait style, potentially affecting the gait biomechanics [18]. Specifically, when the shoe's size or shape does not align with the foot, it may cause foot pain and fatigue, while also reducing stability and increasing the risk of falls during walking [19]. Therefore, conditions different from those in daily life may alter an individual's natural gait patterns and may not accurately reflect habitual walking [11].
As a more suitable evaluation method, Inertial Measurement Units (IMUs) can be used. IMUs can estimate three-dimensional movement from angular velocity and acceleration [20,21]. Due to their low cost, small size, and ease of use, they have been widely used to evaluate daily life walking in recent years [22]. By using the In-Shoe Motion Sensor system (IMS), which measures the acceleration and angular velocity of the feet using IMUs and calculates walking parameters, it is possible to obtain walking data comparable to daily life more accurately than conventional studies [23].
Line numbers where the changed text appears: 86-119

(The introduction for Discussion)

Powered by Editorial Manager® and ProduXion Manager® from Aries Systems Corporation

This study aimed to evaluate the effect of high-heeled shoes on gait parameters in healthy adult women using an IMS system. In previous studies, participants often wore unfamiliar shoes and measurements were commonly conducted on a treadmill at a constant speed, which may not accurately reflect natural gait patterns in real-life settings. In contrast, this study utilized a compact and user-friendly IMS to collect gait data while participants wore their own familiar shoes. This approach allowed for accurate collection in a manner closer to daily life conditions, providing foundational data for assessing the effects of footwear under more realistic circumstances.
Line numbers where the changed text appears: 238-246

(Conclusion)
Because this study collected gait data under conditions similar to those of daily life, it provides data suitable for practical applications and may contribute to future research focused on evaluating gait in everyday scenarios.
Line numbers where the changed text appears: 355-364

[Comment #2]
- There is no sample size estimation in your study. One would always like to conduct a study that has adequate sample size and power so that the conclusions generated from the experiment can be applied to the broader population with ample confidence. The required sample size to test a hypothesis is governed by the effect size. Sample size was not calculated in the study. The power of the study should be calculated and added. Because, our aim should be to catch the most significant and best difference by performing the strongest statistical tests with the optimal sample size. Sample size calculation is of great importance here.
[Author action #2]
Thank you for your valuable comment. The sample size for this study was estimated based on the findings of Chard et al (2013), a prior study closely related to our research. Specifically, we calculated a sample size of 15 participants needed to detect significant differences, using an effect size of 0.62, an α level of 0.05, and a statistical power of 0.8. Additionally, we accounted for a potential dropout rate by recruiting several more 5 participants to ensure adequate power for data analysis. Details of this estimation are included in the Participants section.
The changed text is highlighted in yellow as follows:
The sample size for this study was calculated using G Power 3.1 (Heinrich Heine University). Based on prior research [24], with an assumed effect size of 0.62, an alpha level of 0.05, and a statistical power of 0.8, the minimum required number of participants was calculated to be 15. To account for potential data loss or incomplete data, an additional five participants were included, setting the final target number of participants at 20.
Line numbers where the changed text appears:132-136

(Additional references)
24. Chard A, Greene A, Hunt A, Vanwanseele B, Smith R. Effect of thong style flip-flops on children's barefoot walking and jogging kinematics. Journal of Foot and Ankle Research. 2013;6:8. doi:https://doi.org/10.1186/1757-1146-6-8

[Comment #3]
Since no power analysis was performed and it is difficult to interpret the obtained findings, thus, the discussion.
[Author action #3]
Thank you for your valuable comment. The sample size for this study was estimated based on the findings of Chard et al (2013), a prior study closely related to our research. Specifically, we calculated a sample size of 15 participants needed to detect significant differences, using an effect size of 0.62, an α level of 0.05, and a statistical power of 0.8. Additionally, we accounted for a potential dropout rate by recruiting several more 5 participants to ensure adequate power for data analysis. Based on this estimation, we believe the sample size in our study is appropriate. Thank you for your understanding. Details of this estimation are included in the Participants section.
The changed text is highlighted in yellow as follows:
The sample size for this study was calculated using G Power 3.1 (Heinrich Heine University). Based on prior research [24], with an assumed effect size of 0.62, an alpha level of 0.05, and a statistical power of 0.8, the minimum required number of

participants was calculated to be 15. To account for potential data loss or incomplete data, an additional five participants were included, setting the final target number of participants at 20.
Line numbers where the changed text appears: 132-136

(Additional references)
24. Chard A, Greene A, Hunt A, Vanwanseele B, Smith R. Effect of thong style flip-flops on children's barefoot walking and jogging kinematics. Journal of Foot and Ankle Research. 2013;6:8. doi:https://doi.org/10.1186/1757-1146-6-8

Reviewer #3:
[Comment #1]
Introduction:
Clarity of Rationale: The introduction touches upon various studies but lacks a clear argument for why this study is unique. It's essential to emphasize the gaps in previous research more clearly. For instance, highlight that previous studies used treadmill walking or unfamiliar footwear, and explain why in-shoe motion sensors offer a more reliable approach.
[Author action #1]
Thank you for your feedback. As you pointed out, we had not clearly articulated the gap between this study and previous research. Based on your suggestions, we have added a rationale explaining the issues with using treadmill walking and unfamiliar footwear, as well as why motion sensors provide a more reliable approach.
The changed text is highlighted in yellow as follows:
Research on the effects of high-heeled shoes on gait parameters has significantly progressed. However, many evaluations have been conducted using a treadmill with participants walking at a constant speed [14]. Treadmill walking occurs under flat, steady-speed conditions, which differs from everyday walking environments [15]. For example, because a treadmill maintains a fixed speed, participants cannot walk at their natural pace, potentially suppressing their typical gait patterns [16,17]. Additionally, on a treadmill, individuals often need to adjust their posture and gait mechanics to match the treadmill's motion in order to maintain visual and balance cues, which is known to influence gait biomechanics [15]. In certain studies, participants wore unfamiliar shoes that may not accurately reflect their usual gait patterns in daily life [11,14]. Shoes that are unfamiliar or uncomfortable for an individual often do not fit well with the person's foot shape or gait style, potentially affecting the gait biomechanics [18]. Specifically, when the shoe's size or shape does not align with the foot, it may cause foot pain and fatigue, while also reducing stability and increasing the risk of falls during walking [19]. Therefore, conditions different from those in daily life may alter an individual's natural gait patterns and may not accurately reflect habitual walking [11].
As a more suitable evaluation method, Inertial Measurement Units (IMUs) can be used. IMUs can estimate three-dimensional movement from angular velocity and acceleration [20,21]. Due to their low cost, small size, and ease of use, they have been widely used to evaluate daily life walking in recent years [22]. By using the In-Shoe Motion Sensor system (IMS), which measures the acceleration and angular velocity of the feet using IMUs and calculates walking parameters, it is possible to obtain walking data comparable to daily life more accurately than conventional studies [23].
Line numbers where the changed text appears: 86-119

[Comment #2]
Study Objectives: The objective of the study is mentioned indirectly, but it should be explicitly stated at the end of the introduction. Clearly articulate the novelty and specific aims of your study beyond what has been done before.
[Author action #2]
Thank you for your insightful feedback. In response to your comments, we have clarified the objectives of our study, highlighting how it differs from previous research.
The changed text is highlighted in yellow as follows:
Therefore, this study aimed to evaluate the impact of high-heeled shoes on gait parameters in healthy adult women using the IMS system. Unlike previous studies that typically used treadmill walking at a constant speed or required participants to wear unfamiliar shoes, This study allowed participants to walk in their own footwear that fitted their feet and that they were accustomed to, under conditions similar to those of daily life. This approach enabled us to capture a more natural gait pattern, closely resembling everyday walking, and to more accurately reflect the effects of high-heeled

shoes on gait parameters.
Line numbers where the changed text appears: 113-119

[Comment #3]
Methods:
Participant Recruitment and Demographics: The participant demographics (age, height, weight) are provided, but you need to offer more details on how participants were selected. For example, how did the recruitment process ensure variability in walking experience with high heels? Were any baseline tests performed to evaluate walking proficiency in high heels?
[Author action #3]
Thank you for your feedback. In this study, we primarily targeted women who commonly have opportunities to wear high-heeled shoes in daily life, based on previous research (Annoni et al., 2023). In the revised manuscript, we have described the participant selection process in as much detail as possible based on this context. I would appreciate it if you could review it at your convenience.
The changed text is highlighted in yellow as follows:
The participants in this study were healthy adult women enrolled at Kyoto University. The inclusion criteria were individuals aged 18 years and older who could walk for at least 6 minutes without rest. To minimize the influence of unfamiliarity with high-heeled shoes on the results, we selected participants with extensive experience wearing high heels in various settings, such as work, social events, shopping, or formal occasions, based on previous studies [14]. The exclusion criteria were individuals with neurological or orthopedic conditions that could affect walking function. The purpose of the study was thoroughly explained to the participants, and their consent was obtained prior to measurement.
Line numbers where the changed text appears: 127-129

(Additional references)
14. Annoni I, Mapelli A, Sidequersky FV, Zago M, Sforza C. The effect of high-heeled shoes on overground gait kinematics in young healthy women. Sport Sciences for Health. 2014;10(2):149-157. doi: 10.1007/s11332-014-0191-z

[Comment #4]
Sample Size: The study includes only 17 participants. Was a power analysis conducted to determine whether this sample size is sufficient for detecting significant differences? This omission weakens the study’s generalizability and statistical power. Justify the sample size or conduct a power analysis to support your choices.
Shoe Definitions and Comparisons: The criteria for shoe selection are unclear and could be inconsistent. The manuscript states that high-heeled shoes are defined by their heel height but also includes other characteristics like a "narrow toe box." This could lead to confusion about what exactly constitutes a "high-heeled" shoe. A more specific definition is needed.
[Author action #4]
Thank you for your valuable comment. The sample size for this study was estimated based on the findings of Chard et al (2013), a prior study closely related to our research. Specifically, we calculated a sample size of 15 participants needed to detect significant differences, using an effect size of 0.62, an α level of 0.05, and a statistical power of 0.8. Additionally, we accounted for a potential dropout rate by recruiting several more 5 participants to ensure adequate power for data analysis. Based on this estimation, we believe the sample size in our study is appropriate. Thank you for your understanding. Details of this estimation are included in the Participants section.
The changed text is highlighted in yellow as follows:
The sample size for this study was calculated using G Power 3.1 (Heinrich Heine University). Based on prior research [24], with an assumed effect size of 0.62, an alpha level of 0.05, and a statistical power of 0.8, the minimum required number of participants was calculated to be 15. To account for potential data loss or incomplete data, an additional five participants were included, setting the final target number of participants at 20.
Line numbers where the changed text appears: 132-136

(Additional references)
24. Chard A, Greene A, Hunt A, Vanwanseele B, Smith R. Effect of thong style flip-flops on children’s barefoot walking and jogging kinematics. Journal of Foot and Ankle

Research. 2013;6:8. doi:https://doi.org/10.1186/1757-1146-6-8

Regarding definitions, in this study, high-heeled shoes were defined as “footwear with a heel elevated higher than the forefoot, a narrow toe box, and a curved sole.” This definition is based on existing literature that has demonstrated the specific effects of high heels on walking parameters. In contrast, sneakers were defined as “light footwear that has a top made of cloth and a bottom made of rubber.” To the best of our knowledge, no academic definition of “sneakers” was available; thus, we referred to the general definition from the Cambridge Dictionary. Sneakers were chosen as the control group because obtaining walking data with footwear familiar to participants allows for a walking pattern more reflective of daily life.
These definitions were included in the “Equipment” section as follows:

High-heeled shoes were defined as “footwear with a heel elevated higher than the forefoot, a narrow toe box, and a curved sole” [11], while sneakers were defined as “light footwear that has a top made of cloth and a bottom made of rubber.”

Line numbers where the changed text appears: 173-176

[Comment #5]
Data Collection Process: The measurement process with IMS should be explained with greater detail. For example, how were the intervals between measurements selected? Why are only three steps used for calculating parameters? Provide a rationale for using these specific methods, or cite prior studies that validate this approach.
[Author action #5]
Thank you for your valuable feedback. The above methods are established by the manufacturer according to prior research, and their accuracy is validated through rigorous validation processes. I have added relevant prior studies and provided a more detailed description. I would appreciate it if you could review it at your convenience.
The changed text is highlighted in yellow as follows:
Equipment
The IMS System (A-RROWG, NEC Corporation, Tokyo, Japan) was used for the measurements. The IMS is a device used for gait analysis and consists of key components including an ARM Cortex-M4F MCU (nRF52832 by Nordic Semiconductor). This component features a 64 MHz CPU, 64 KB of RAM, and 512 KB of ROM. Additionally, it includes a Bosch IMU (BMI160) for precise motion tracking, an ABLIC EEPROM (S-24C32C, 32K-bit) for storage, and an Epson real-time clock (RX8130CE). This device performs gait measurements at 2-minute intervals, capturing gait parameters on both sides. The system detects the gait cycle during flat walking, operates for 1 min, and then enters sleep mode to conserve energy. During this 1-minute period, it detects vibrations and identifies stable walking patterns, and upon detecting the start of a gait cycle, it measures the parameters for three steps within the following 9 s. These steps do not need to be consecutive; if the three steps are successfully measured within a specified time, the average value is recorded. If fewer than three steps were measured, the average value was not recorded. Additionally, this measurement process can be repeated up to three times within a 1-minute window; if all three attempts fail, the measurement for that period is considered unsuccessful, and no data is recorded [25,26,27].
The following 16 parameters are calculated: Stride length, Maximum (peak) dorsiflexion angle, Maximum (peak) plantar flexion angle, Foot clearance, Foot height, Circumduction, Toe-in/out angle, Roll angle of heel contact, Roll angle of toe-off, Walking speed, Peak swing angular velocity, Maximum speed during swinging phase, Stance time, Swing time, Pushing time, and Cadence. Table 1 below, disclosed by NEC Corporation, categorizes the definitions of each parameter into spatial, spatiotemporal, and temporal parameters. These parameters were validated in previous studies for accuracy or were derived from validated parameters [25,26,27].
Line numbers where the changed text appears: 143-171

[Comment #6]
Results:
Lack of Effect Sizes: The results section presents P-values but lacks effect sizes, which are crucial for understanding the magnitude of differences between high heels and sneakers. Please include effect sizes for all statistically significant results.
[Author action #6]

Thank you for your valuable feedback regarding the need to include effect sizes in the results section. As suggested, we have now included effect sizes for all statistically significant results. Specifically, we have calculated both Cohen's d and Hedges' g to provide a clearer understanding of the magnitude of the differences between the conditions (high heels vs. sneakers).
The changed table is highlighted in yellow as follows:
ClassificationParametersHGSGP-valueEffect Size (Cohen's d)
Spatial parametersStride length (cm)130.42±18.14143.88±16.090.0006*-1.09
Maximum (peak) dorsiflexion angle (degree)24.21±5.2529.88±5.130.0017*-0.94
Maximum (peak) plantar flexion angle (degree)62.33±11.1578.31±6.49<0.0001*-1.47
Foot clearance (cm)21.17±3.3124.05±2.150.0005*-1.09
Foot height (cm)13.33±2.4015.70±1.760.0023*-0.90
Circumduction (cm)4.10±1.583.72±2.010.53970.15
Toe-in/out angle (degree)12.05±8.8210.14±7.970.0476*0.54
Roll angle of heel contact (degree)5.21±4.863.71±5.700.23430.30
Roll angle of toe-off (degree)-2.05±6.17-1.36±4.520.5178-0.17
Spatiotemporal parametersWalking speed (km/h)4.61±0.685.08±0.660.0005*-1.09
Peak swing angular velocity (degree/s)531.64±95.96565.64±75.540.0932-0.45
Maximum speed during swinging phase (km/h)15.15±1.6515.97±1.590.0085*-0.75
Temporal parametersStance time(s)0.62±0.050.62±0.040.8271-0.06
Swing time(s)0.41±0.040.40±0.010.32250.03
Pushing time (s)0.19±0.030.20±0.020.6805-0.13
Cadence (steps/min)117.22±6.41116.92±5.690.80300.06
SD, standard deviation. *p<0.05.
Effect size (Cohen's d): Small = 0.2–0.49, Medium = 0.5–0.79, Large ≥ 0.8

Line numbers where the changed text appears: 232

[Comment #7]
Detailed Parameter Reporting: Some parameters such as circumduction and roll angle show no significant difference but are still discussed in detail. Revisit how you report and discuss non-significant findings. Focus should be on significant outcomes unless there is a theoretical justification for the non-significant results.
[Author action #7]
Thank you for your valuable feedback. We acknowledge that providing theoretical justification for non-significant findings is challenging. To maintain the quality of our study, we have removed discussions on non-significant parameters and have elaborated more thoroughly on the significant results.

[Comment #8]
Discussion:
In-depth Analysis: The discussion needs more depth, especially concerning the mechanisms underlying the changes in gait parameters. For example, why do high heels specifically reduce stride length and foot clearance? Refer to the biomechanical literature more thoroughly.
[Author action #8]
Thank you very much for your valuable feedback. As per your suggestion, I have provided a more detailed discussion. I would appreciate it if you could review it at your convenience. The changed table is highlighted in yellow as follows:
Regarding spatial parameters such as walking speed, stride length, and maximum swing speed, it is generally known that wearing high-heeled shoes reduces the base of support during walking, prompting individuals to adopt a safer and more stable gait pattern [28,29]. Additionally, to minimize increases in energy expenditure, they tend to decrease their walking speed and stride length as a result [30,31]. In addition to these stable and efficient walking strategies, previous studies have shown that wearing high heels brings the ground reaction force closer to the ankle joint, and the curved shape of the shoe sole causes the ankle to remain in a plantarflexed position [11]. These factors can shorten the length of the triceps surae muscle and Achilles tendons. Consequently, compared to sneakers or barefoot walking, ankle plantarflexion moments decrease, and the effect of the stretch-shortening cycle on the muscle-tendon complex is reduced [32,33]. The stretch-shortening cycle is a process in which muscles and tendons quickly stretch and then shorten, store, and release elastic energy, and plays a crucial role in efficient force production [33]. However, the shortened state of the muscle-tendon complex induced by wearing high heels is

Powered by Editorial Manager® and ProduXion Manager® from Aries Systems Corporation

	thought to impede this function, leading to decreased energy efficiency and a weaker push-off force. As a result, the calculated parameters were significantly different between the two groups.<br>Similarly, foot clearance and foot height were significantly lower in the high-heeled group. As noted above, wearing high-heeled shoes is suggested to reduce the push-off force of the ankle. Additionally, previous studies investigating the role of the ankle in walking have shown that ankle push-off contributes to up to 50% of the power needed for leg swinging during the terminal stance and early swing phases [34,35,36]. This reduction in ankle push-off may decrease the leg swing speed, which is essential for the double-pendulum effect during walking [37]. Consequently, a lack of sufficient knee flexion in the double-pendulum mechanism may cause the lower leg to remain relatively low, potentially resulting in reduced foot clearance. In fact, prior research comparing joint angles in the sagittal plane under different heel heights at a constant walking speed found that while hip flexion during the swing phase remained unaffected by heel height, the knee flexion angle significantly decreased as heel height increased [38,39,40]. Moreover, a decrease in the foot clearance height can increase the risk of tripping over small obstacles or uneven surfaces while walking, potentially leading to falls [41]. High-heeled shoes also tilt the toes downward and elevate the heel, reducing the contact area of the foot with the ground, which may further destabilize balance. This combination of reduced foot clearance and a limited base of support likely exacerbates the risk of falls [14].<br>Line numbers where the changed text appears: 266-303<br><br>[Comment #9]<br>Missing Literature Context: While the discussion refers to previous studies, it does not critically evaluate their methods or findings in relation to the current study. More effort should be made to situate your results within the broader field of biomechanics and footwear research.<br>[Author action #9]<br>Thank you very much for your valuable feedback. As you suggested, I have clarified the theoretical rationale and need for this study in the Discussion, especially in comparison with prior studies. In doing so, we re-evaluated the novelty of this study, which we believe primarily consists of the following three aspects:<br>1.Measurement under conditions close to daily life: Unlike other studies that use prescribed footwear, our study measured participants in their habitual shoes, collecting data that more accurately represents natural, everyday walking styles. Furthermore, many previous studies have conducted measurements on treadmills, which differ significantly from actual walking conditions. By performing measurements in real-life conditions, our study is able to provide practical insights that more realistically reflect individual walking patterns.<br>2.High-precision data collection of daily movement using IMS: Instead of traditional motion capture systems or treadmill-based experiments, we utilized the In-Shoe Motion Sensor (IMS) system, allowing measurements in an environment closer to everyday life. This enables a realistic evaluation of the impact of wearing high heels in typical environments outside the laboratory, offering valuable feedback applicable to real-world conditions.<br>3.Practical application perspective: The data collection method using IMS serves as foundational data that can be leveraged to evaluate fall risks in everyday walking and provide feedback for designing comfortable and safe footwear. This data can be ap...
**Additional Information:**	
**Question**	**Response**
**Financial Disclosure**<br><br>Enter a financial disclosure statement that describes the sources of funding for the work included in this submission. Review the submission guidelines for detailed requirements. View published research articles from *PLOS ONE* for specific examples.	The author(s) received no specific funding for this work.

This statement is required for submission and **will appear in the published article** if the submission is accepted. Please make sure it is accurate.<br><br>**Funded studies**<br>Enter a statement with the following details:<br>• Initials of the authors who received each award<br>• Grant numbers awarded to each author<br>• The full name of each funder<br>• URL of each funder website<br>• Did the sponsors or funders play any role in the study design, data collection and analysis, decision to publish, or preparation of the manuscript?<br><br>Did you receive funding for this work?	
**Competing Interests**<br><br>Use the instructions below to enter a competing interest statement for this submission. On behalf of all authors, disclose any competing interests that could be perceived to bias this work—acknowledging all financial support and any other relevant financial or non-financial competing interests.<br><br>This statement is required for submission and **will appear in the published article** if the submission is accepted. Please make sure it is accurate and that any funding sources listed in your Funding Information later in the submission form are also declared in your Financial Disclosure statement.<br><br>View published research articles from *PLOS ONE* for specific examples.	The authors have declared that no competing interests exist.

**NO authors have competing interests**<br><br>Enter: *The authors have declared that no competing interests exist.*<br><br>**Authors with competing interests**<br><br>Enter competing interest details beginning with this statement:<br><br>*I have read the journal's policy and the authors of this manuscript have the following competing interests: [insert competing interests here]*<br><br>* typeset	
**Ethics Statement**<br><br>Enter an ethics statement for this submission. This statement is required if the study involved:<br><br>• Human participants<br>• Human specimens or tissue<br>• Vertebrate animals or cephalopods<br>• Vertebrate embryos or tissues<br>• Field research<br><br>Write "N/A" if the submission does not require an ethics statement.<br><br>General guidance is provided below. Consult the submission guidelines for detailed instructions. **Make sure that all information entered here is included in the Methods section of the manuscript.**	This study was approved by the Kyoto University Medical Ethics Committee (Approval number: R3664-3). Prior to study initiation, written informed consent was obtained from all participants in accordance with the ethical standards and guidelines.

Powered by Editorial Manager® and ProduXion Manager® from Aries Systems Corporation

**Format for specific study types**<br><br>**Human Subject Research (involving human participants and/or tissue)**<br>• Give the name of the institutional review board or ethics committee that approved the study<br>• Include the approval number and/or a statement indicating approval of this research<br>• Indicate the form of consent obtained (written/oral) or the reason that consent was not obtained (e.g. the data were analyzed anonymously)<br><br>**Animal Research (involving vertebrate animals, embryos or tissues)**<br>• Provide the name of the Institutional Animal Care and Use Committee (IACUC) or other relevant ethics board that reviewed the study protocol, and indicate whether they approved this research or granted a formal waiver of ethical approval<br>• Include an approval number if one was obtained<br>• If the study involved *non-human primates*, add *additional details* about animal welfare and steps taken to ameliorate suffering<br>• If anesthesia, euthanasia, or any kind of animal sacrifice is part of the study, include briefly which substances and/or methods were applied<br><br>**Field Research**<br><br>Include the following details if this study involves the collection of plant, animal, or other materials from a natural setting:<br>• Field permit number<br>• Name of the institution or relevant body that granted permission	
**Data Availability**<br><br>Authors are required to make all data underlying the findings described fully available, without restriction, and from the time of publication. PLOS allows rare exceptions to address legal and ethical concerns. See the PLOS Data Policy and FAQ for detailed information.	Yes - all data are fully available without restriction

A Data Availability Statement describing where the data can be found is required at submission. Your answers to this question constitute the Data Availability Statement and **will be published in the article**, if accepted.<br><br>**Important:** Stating 'data available on request from the author' is not sufficient. If your data are only available upon request, select 'No' for the first question and explain your exceptional situation in the text box.<br><br>Do the authors confirm that all data underlying the findings described in their manuscript are fully available without restriction?	
**Describe where the data may be found in full sentences. If you are copying our sample text, replace any instances of XXX with the appropriate details.**<br><br>• If the data are **held or will be held in a public repository**, include URLs, accession numbers or DOIs. If this information will only be available after acceptance, indicate this by ticking the box below. For example: *All XXX files are available from the XXX database (accession number(s) XXX, XXX.*).<br>• If the data are all contained **within the manuscript and/or Supporting Information files**, enter the following: *All relevant data are within the manuscript and its Supporting Information files.*<br>• If neither of these applies but you are able to provide **details of access elsewhere**, with or without limitations, please do so. For example:<br><br>*Data cannot be shared publicly because of [XXX]. Data are available from the XXX Institutional Data Access / Ethics Committee (contact via XXX) for researchers who meet the criteria for access to confidential data.*<br><br>*The data underlying the results presented in the study are available from (include the name of the third party*	We uploaded as supplementary information

and contact information or URL).<br>• This text is appropriate if the data are owned by a third party and authors do not have permission to share the data.<br><br>* typeset	
Additional data availability information:	

Powered by Editorial Manager® and ProduXion Manager® from Aries Systems Corporation

Manuscript Click here to access/download;Manuscript;Manuscript.ver2_Kyoma

The Effects of High-Heeled Shoes on Gait Parameters in Healthy Adult Women.

Short Title: The effects of high-heeled shoes on gait parameters

Kyoma Tanigawa[a], Hiroki Shimizu[a], Anuradhi Bandara[a], Toyota Misa[a], Shota Suzuki[a], Momoko Nagai-Tanima[a], Tomoki Aoyama*[a]

[a]Department of Physical Therapy, Human Health Sciences, Graduate School of Medicine, Kyoto University, Kyoto, 606-8507, Japan

*Corresponding author: Tomoki Aoyama

E-mail: aoyama.tomoki.4e@kyoto-u.ac.jp

Abstract

Background

Walking is essential in daily life, and footwear type significantly affects walking patterns. High-heeled shoes increase the risk of knee osteoarthritis and falls in women. Traditional studies often use treadmills or unfamiliar footwear, which may not reflect daily walking. This study investigates the impact of high-heeled shoes on walking parameters in healthy adult women using in-shoe motion sensors.

Methods

Seventeen healthy adult women without pain during walking participated. They walked for 6 minutes along a 30-meter corridor wearing high-heeled shoes and sneakers. Walking data were recorded using an in-shoe motion sensor system every 2 minutes. The average of three valid consecutive steps was calculated automatically. Statistical analysis compared the mean walking parameters between the high-heel and sneaker groups.

Results

The high-heel group showed significantly lower foot clearance, stride length, peak plantar angle in dorsiflexion, and peak plantar angle in plantarflexion, and significantly higher toe-out angle in spatial parameters. Spatiotemporal parameters revealed significantly lower walking speed and maximum swing

phase speed in the high-heel group. No significant differences were observed in temporal parameters between the groups.

Conclusions

Since this study collected gait data under conditions similar to daily life, it provides data suitable for practical applications and may contribute to future research evaluating everyday gait. Additionally, future studies should include a broader range of participants and incorporate measurement devices capable of capturing hip and knee joint movements, providing a more comprehensive evaluation of the effects of high-heeled shoes on gait in healthy adult women.

Introduction

Walking is a crucial means of transporting the body safely and efficiently [1]. Additionally, it has beneficial effects on both physical and mental health, enhances human quality of life (QOL), and plays a significant role in the prevention and treatment of various diseases [2]. As such, walking is indispensable in daily life. It is also known that walking is influenced by various external factors [3].

One of these external factors is footwear. Shoes come in various types and have multiple functions, including protecting the feet from external environments, assisting walking functions, and adapting to foot deformities. These functions are related to the occurrence of various conditions

such as falls, osteoarthritis, and back pain [4]. Numerous studies have investigated the impact of shoes on walking parameters [5,6]. For example, research comparing walking barefoot and wearing athletic shoes by Shakoor et al. found that wearing athletic shoes decreases step length, increases the number of steps, and reduces joint angles of the lower limbs [5]. Thus, research on how shoes affect walking is of great importance [7].

One type of shoe that warrants attention is high-heeled shoes. High-heeled shoes are characterized by heels higher than the forefoot, narrow toe boxes, and curved soles [8]. High-heeled shoes have been widely worn by women worldwide for centuries, with 37% to 69% of women wearing them regularly and for an average of 1 to 8 hours per day [8]. Despite their regular use, high-heeled shoes are associated with medical conditions, such as osteoarthritis, and can cause frequent falls [9]. The incidence of such injuries doubled from 7.1% to 14.2% between 2002 and 2012 [10]. Therefore, it is crucial to investigate the impact of high-heeled shoes on the walking parameters of healthy adult women [7]. Several previous studies have examined this effect [9,11,12,13]. For instance, it has been shown that wearing high-heeled shoes does not change the temporal walking parameters such as stance time and cadence [11]. Additionally, a study by Barkema et al. noted that the higher the heel height, the greater the maximum knee adduction moment, indicating a relationship between heel height and knee osteoarthritis [9].

Research on the effects of high-heeled shoes on gait parameters has significantly progressed. However, many evaluations have been conducted using a treadmill with participants walking at a constant speed [14]. Treadmill walking occurs under flat, steady-speed conditions, which differs from everyday walking environments [15]. For example, because a treadmill maintains a fixed speed, participants cannot walk at their natural pace, potentially suppressing their typical gait patterns [16,17]. Additionally, on a treadmill, individuals often need to adjust their posture and gait mechanics to match the treadmill's motion in order to maintain visual and balance cues, which is known to influence gait biomechanics [15]. In certain studies, participants wore unfamiliar shoes that may not accurately reflect their usual gait patterns in daily life [11,14]. Shoes that are unfamiliar or uncomfortable for an individual often do not fit well with the person's foot shape or gait style, potentially affecting the gait biomechanics [18]. Specifically, when the shoe's size or shape does not align with the foot, it may cause foot pain and fatigue, while also reducing stability and increasing the risk of falls during walking [19]. Therefore, conditions different from those in daily life may alter an individual's natural gait patterns and may not accurately reflect habitual walking [11].

As a more suitable evaluation method, Inertial Measurement Units (IMUs) can be used. IMUs can estimate three-dimensional movement from angular velocity and acceleration [20,21]. Due to their low cost, small size, and ease of use, they have been widely used to evaluate daily life walking

in recent years [22]. By using the In-Shoe Motion Sensor system (IMS), which measures the acceleration and angular velocity of the feet using IMUs and calculates walking parameters, it is possible to obtain walking data comparable to daily life more accurately than conventional studies–[23].

Therefore, this study aimed to evaluate the impact of high-heeled shoes on gait parameters in healthy adult women using the IMS system. Unlike previous studies that typically used treadmill walking at a constant speed or required participants to wear unfamiliar shoes, this study allowed participants to walk in their own footwear that fitted their feet and that they were accustomed to, under conditions similar to those of daily life. This approach enabled us to capture a more natural gait pattern, closely resembling everyday walking, and to more accurately reflect the effects of high-heeled shoes on gait parameters.

Materials and Methods

Study design

This was an intervention study.

Participants

The participants in this study were healthy adult women enrolled at Kyoto University. The inclusion criteria were individuals aged 18 years and older who could walk for at least 6 minutes without rest. To minimize the influence of unfamiliarity with high-heeled shoes on the results, we

selected participants with extensive experience wearing high heels in various settings, such as work, social events, shopping, or formal occasions, based on previous studies [14]. The exclusion criteria were individuals with neurological or orthopedic conditions that could affect walking function. The purpose of the study was thoroughly explained to the participants, and their consent was obtained prior to measurement. The sample size for this study was calculated using G Power 3.1 (Heinrich Heine University). Based on prior research [24], with an assumed effect size of 0.62, an alpha level of 0.05, and a statistical power of 0.8, the minimum required number of participants was calculated to be 15. To account for potential data loss or incomplete data, an additional five participants were included, setting the final target number of participants at 20.

Ethics statement

This study was approved by the Kyoto University Medical Ethics Committee (Approval number: R3664-3). Prior to study initiation, written informed consent was obtained from all participants in accordance with the ethical standards and guidelines.

Equipment

The IMS System (A-RROWG, NEC Corporation, Tokyo, Japan) was used for the measurements. The IMS is a device used for gait analysis and consists of key components including an ARM

Cortex-M4F MCU (nRF52832 by Nordic Semiconductor). This component features a 64 MHz CPU, 64 KB of RAM, and 512 KB of ROM. Additionally, it includes a Bosch IMU (BMI160) for precise motion tracking, an ABLIC EEPROM (S-24C32C, 32K-bit) for storage, and an Epson real-time clock (RX8130CE). This device performs gait measurements at 2-minute intervals, capturing gait parameters on both sides. The system detects the gait cycle during flat walking, operates for 1 min, and then enters sleep mode to conserve energy. During this 1-minute period, it detects vibrations and identifies stable walking patterns, and upon detecting the start of a gait cycle, it measures the parameters for three steps within the following 9 s. These steps do not need to be consecutive; if the three steps are successfully measured within a specified time, the average value is recorded. If fewer than three steps were measured, the average value was not recorded. Additionally, this measurement process can be repeated up to three times within a 1-minute window; if all three attempts fail, the measurement for that period is considered unsuccessful, and no data is recorded [25,26,27].

The following 16 parameters are calculated: Stride length, Maximum (peak) dorsiflexion angle, Maximum (peak) plantar flexion angle, Foot clearance, Foot height, Circumduction, Toe-in/out angle, Roll angle of heel contact, Roll angle of toe-off, Walking speed, Peak swing angular velocity, Maximum speed during swinging phase, Stance time, Swing time, Pushing time, and Cadence. Table 1 below, disclosed by NEC Corporation, categorizes the definitions of each parameter into

spatial, spatiotemporal, and temporal parameters. These parameters were validated in previous studies for accuracy or were derived from validated parameters [25,26,27].

High-heeled shoes were defined as “footwear with a heel elevated higher than the forefoot, a narrow toe box, and a curved sole” [11], while sneakers were defined as “light footwear that has a top made of cloth and a bottom made of rubber.”

Table 1. Definitions of Each Walking Parameter.

Classification	Parameters	Definition
**Spatial parameters**	Stride length (cm)	Distance from the starting point to end point of the foot trajectory
	Maximum (peak) dorsiflexion angle (degree)	Peak foot-sole angle in dorsiflexion
	Maximum (peak) plantar flexion angle (degree)	Peak foot-sole angle in plantarflexion
	Foot clearance (cm)	Maximum heel height of the foot trajectory
	Foot height (cm)	Maximum vertical height of the foot trajectory

	Circumduction (cm)	Displacement in the medial–lateral direction during the swing phase
	Toe-in/out angle (degree)	Average adduction/abduction angle during the swing phase
	Roll angle of heel contact (degree)	Plantar roll angle when the heel touches the ground
	Roll angle of toe-off (degree)	Plantar roll angle when the toe leaves the ground
**Spatiotemporal parameters**	Walking speed (km/h)	Stride length divided by stride time
	Peak swing angular velocity (degree/s)	Peak angular velocity of the foot during the swing phase
	Maximum speed during swinging phase (km/h)	Maximum forward speed of the swinging leg during the swinging phase
**Temporal parameters**	Stance time(s)	Amount of time that the foot is on the ground during the gait cycle

	Swing time(s)	Amount of time that the foot is off the ground during the gait cycle
	Pushing time (s)	Time between the heel contact and toe leaving the ground.
	Cadence (steps/min)	Number of steps per min

Procedure

The measurements were conducted in a 30-meter corridor at the Faculty of Human Health Sciences building, at Kyoto University. First, we verified whether the shoes worn by the participants met the aforementioned criteria. Subsequently, we measured the age (years), height (cm), body mass(kg), size of the shoes(cm), heel height (cm) of the high-heeled shoes, and mass (g) of each shoe. Prior to data collection, participants walked 5 m with insoles equipped with an IMS inserted into their shoes to ensure that they experienced no discomfort during walking. For data collection, Participants placed a smartphone, used for data recording, in either pocket of their pants, as there were no restrictions on which side to use. They were instructed to begin walking from one end of the corridor, turn around at the opposite end, and continue this back-and-forth walking for 6 min. The measurements were conducted twice: once in sneakers and once in high-heeled shoes, with the order of conditions determined using Excel’s random function. Participants were given the

option to take a break between trials upon request. The observer stood in the middle of the pathway during the test to ensure that the participants walked normally and without safety issues, providing updates on the remaining time every minute. If no data were collected, the 6-minute walking test was repeated.

Data analysis

Statistical analyses were performed using the JMP PRO16 software (SAS Institute). First, the Shapiro-Wilk test was conducted to determine whether each dataset followed a normal distribution. For data that were normally distributed, a paired t-test was used, because the same participants were measured under different conditions (high-heeled shoes and sneakers). For data that did not follow a normal distribution, the Wilcoxon signed-rank test, suitable for paired datasets, was applied. The group wearing high-heeled shoes was referred to as the "high-heeled group" (HG) and the group wearing sneakers as the "sneaker group" (SG). Data are presented as mean standard deviation ± standard deviation (SD). The effect size was evaluated using Cohen's d to assess the magnitude of the differences between groups. The statistical significance level for this study was set at less than 5%.

Results

A. Physical Characteristics (Table 2)

There were 17 participants in the study, with no dropouts or adverse events reported. The mean values were as follows: age, 23.4 years; height, 161.78 cm; body mass, 53.46 kg; BMI, 20.38; size of shoes, 24.15 cm; mass of high-heeled shoes, 201.76 g; mass of sneakers, 265.00 g; heel height of high-heeled shoes, 4.93 cm (Table1).

Table 2. Basic Information of Participants.

Characteristics	Mean±SD(N=17)
Age(years)	23.40±1.23
Height(cm)	161.78±6.14
Body Mass(kg)	53.46±5.61
BMI(kg/m^2)	20.38±1.09
Size of shoes(cm)	24.15±1.01
Mass of heel shoes(g)	201.76±37.12
Mass of sneakers(g)	265.00±59.48
Heel height of heel shoes(cm)	4.93±1.17

SD, standard deviation; BMI, body mass index

B. Comparison of Walking Parameters Between High-Heeled and Sneaker Shoes (Table 3)

From the table 3, in the comparison between the high-heeled group and the sneaker group, spatial parameters showed that the high-heeled group had a significantly shorter stride length, lower peak dorsiflexion angle of the foot, lower peak plantar flexion angle of the foot, and significantly higher toe angle compared to the sneaker group. Regarding spatiotemporal parameters, the high-heeled group exhibited significantly lower walking speed and maximum speed during swinging phase. There were no significant differences observed between the groups in temporal parameters.

Table 3. Comparisons of the mean values of each parameter in each category.

Classification	Parameters	HG	SG	P-value	Effect Size (Cohen's d)
**Spatial parameters**	Stride length (cm)	130.42±18.14	143.88±16.09	0.0006*	-1.09
	Maximum (peak) dorsiflexion angle (degree)	24.21±5.25	29.88±5.13	0.0017*	-0.94

	Maximum (peak) plantar flexion angle (degree)	62.33±11.15	78.31±6.49	<0.0001*	-1.47
	Foot clearance (cm)	21.17±3.31	24.05±2.15	0.0005*	-1.09
	Foot height (cm)	13.33±2.40	15.70±1.76	0.0023*	-0.90
	Circumduction (cm)	4.10±1.58	3.72±2.01	0.5397	0.15
	Toe-in/out angle (degree)	12.05±8.82	10.14±7.97	0.0476*	0.54
	Roll angle of heel contact (degree)	5.21±4.86	3.71±5.70	0.2343	0.30
	Roll angle of toe-off (degree)	-2.05±6.17	-1.36±4.52	0.5178	-0.17
**Spatiotemporal parameters**	Walking speed (km/h)	4.61±0.68	5.08±0.66	0.0005*	-1.09
	Peak swing angular velocity (degree/s)	531.64±95.96	565.64±75.54	0.0932	-0.45

	Maximum speed during swinging phase (km/h)	15.15±1.65	15.97±1.59	0.0085*	-0.75
**Temporal parameters**	Stance time(s)	0.62±0.05	0.62±0.04	0.8271	-0.06
	Swing time(s)	0.41±0.04	0.40±0.01	0.3225	0.03
	Pushing time (s)	0.19±0.03	0.20±0.02	0.6805	-0.13
	Cadence (steps/min)	117.22±6.41	116.92±5.69	0.8030	0.06

SD, standard deviation. *p<0.05.

Effect size (Cohen's d): Small = 0.2–0.49, Medium = 0.5–0.79, Large ≥ 0.8

Discussion

This study aimed to evaluate the effect of high-heeled shoes on gait parameters in healthy adult women using an IMS system. In previous studies, participants often wore unfamiliar shoes and measurements were commonly conducted on a treadmill at a constant speed, which may not accurately reflect natural gait patterns in real-life settings. In contrast, this study utilized a compact and user-friendly IMS to collect gait data while participants wore their own familiar shoes. This

approach allowed for accurate collection in a manner closer to daily life conditions, providing foundational data for assessing the effects of footwear under more realistic circumstances.

As a result, in terms of spatial parameters, the high-heeled group showed significantly shorter stride length, lower maximum dorsiflexion angle, lower maximum plantar flexion angle, and significantly higher toe-out angle compared to the sneaker group, with effect sizes ranging from moderate to high. Additionally, in spatiotemporal parameters, the high-heeled group exhibited significantly lower walking speed and maximum speed during the swinging phase, with large effect sizes. However, no significant differences were observed between the groups in temporal parameters.

Regarding spatial parameters such as walking speed, stride length, and maximum swing speed, it is generally known that wearing high-heeled shoes reduces the base of support during walking, prompting individuals to adopt a safer and more stable gait pattern [28,29]. Additionally, to minimize increases in energy expenditure, they tend to decrease their walking speed and stride length as a result [30,31]. In addition to these stable and efficient walking strategies, previous studies have shown that wearing high heels brings the ground reaction force closer to the ankle joint, and the curved shape of the shoe sole causes the ankle to remain in a plantarflexed position [11]. These factors can shorten the length of the triceps surae muscle and Achilles tendons. Consequently, compared to sneakers or barefoot walking, ankle plantarflexion moments decrease,

and the effect of the stretch-shortening cycle on the muscle-tendon complex is reduced [32,33]. The stretch-shortening cycle is a process in which muscles and tendons quickly stretch and then shorten, store, and release elastic energy, and plays a crucial role in efficient force production [33]. However, the shortened state of the muscle-tendon complex induced by wearing high heels is thought to impede this function, leading to decreased energy efficiency and a weaker push-off force. As a result, the calculated parameters were significantly different between the two groups. Similarly, foot clearance and foot height were significantly lower in the high-heeled group. As noted above, wearing high-heeled shoes is suggested to reduce the push-off force of the ankle. Additionally, previous studies investigating the role of the ankle in walking have shown that ankle push-off contributes to up to 50% of the power needed for leg swinging during the terminal stance and early swing phases [34,35,36]. This reduction in ankle push-off may decrease the leg swing speed, which is essential for the double-pendulum effect during walking [37]. Consequently, a lack of sufficient knee flexion in the double-pendulum mechanism may cause the lower leg to remain relatively low, potentially resulting in reduced foot clearance. In fact, prior research comparing joint angles in the sagittal plane under different heel heights at a constant walking speed found that while hip flexion during the swing phase remained unaffected by heel height, the knee flexion angle significantly decreased as heel height increased [38,39,40]. Moreover, a decrease in the foot clearance height can increase the risk of tripping over small obstacles or uneven surfaces while

walking, potentially leading to falls [41]. High-heeled shoes also tilt the toes downward and elevate the heel, reducing the contact area of the foot with the ground, which may further destabilize balance. This combination of reduced foot clearance and a limited base of support likely exacerbates the risk of falls [14].

Thus, the high-heeled group showed significantly lower stride length, lower peak dorsiflexion angle of the foot, lower peak plantar flexion angle of the foot, lower walking speed, and swing phase maximum velocity, while the toe angle during the swing phase was significantly higher. A higher toe angle indicates a larger ankle abduction angle. Studies investigating the influence of heel height on the anterior-posterior joint moments of the lower limb have shown that as heel height increases, knee internal rotation moments increase, potentially increasing the load on the medial compartment of the knee [9,42]. Conversely, walking with toes pointing outward reduces knee internal rotation moments and decreases the load on the medial compartment [43]. In this study, it is suggested that the high-heeled group adopted a walking strategy with toes pointing outward to reduce the load on the medial compartment of the knee. Additionally, since knee joint movements during stance and toe direction were not measured in this study, further experiments are necessary.

There are limitations in this study; the participants were limited to healthy adult women from the same university, which restricts the generalizability of our findings. To apply these results to other age groups, sexes, or individuals with specific health conditions, further studies with more diverse

participants are necessary. Second, we were unable to capture the movements of the hip and knee joints, making it challenging to fully assess how these joints affect gait mechanics, particularly balance and posture control. Specifically, it remains unclear to what extent high-heeled shoes stress on these joints under near-daily conditions and how this stress might increase the risk of falls or lead to long-term joint disorders. Future research should incorporate devices that measure the movements of these joints to provide a more comprehensive evaluation of the impact of different footwear types on gait and balance.

Conclusions

This study investigated the effects of high-heeled shoes on walking parameters in healthy adult women using the IMS. As a result, in terms of spatial parameters, the high-heeled group showed significantly shorter stride length, lower maximum dorsiflexion angle, lower maximum plantar flexion angle, and significantly higher toe-out angle compared to the sneaker group, with effect sizes ranging from moderate to high. Additionally, in spatiotemporal parameters, the high-heeled group exhibited significantly lower walking speed and maximum speed during the swinging phase, with large effect sizes. However, no significant differences were observed between the groups in temporal parameters. These findings suggest that wearing high-heeled shoes has a substantial impact on gait stability and efficiency, potentially increasing the risk of falls and musculoskeletal

strain. Future studies should include a more diverse population, such as older adults or individuals with joint disorders, and incorporate motion capture systems or wearable sensors that can measure lower limb joint dynamics. These additional studies would allow for a comprehensive understanding of how footwear affects not only the ankle but also hip and knee joint movements across a wider population. Because this study collected gait data under conditions similar to those of daily life, it provides data suitable for practical applications and may contribute to future research focused on evaluating gait in everyday scenarios.

Acknowledgments

We express our gratitude to all participants, instructors, and laboratory members for their cooperation and valuable input throughout this study. We also thank Editage (www.editage.com) for English editing.

References

1. Prince F, Corriveau H., Hebert R, Winter DA. Gait in elderly. Gait & Posture. 1997;5(2): 128-135. doi: 10.1016/S0966-6362(97)01118-1

2. Bai X, Kim GS, Dev RDO, Talib O, Xiao W, Cai H. Effect of Brisk Walking on Health-Related Physical Fitness Balance and Life Satisfaction Among the Elderly: A Systematic Review. Frontiers in Public Health. 2022;9. doi: https://doi.org/10.3389/fpubh.2021.829367
3. Menant JC, Steele JR, Menz HB, Munro BJ, Lord SR. Effects of walking surfaces and footwear on temporo-spatial gait parameters in young and older people. Gait & Posture. 2009;29(3):392-397. doi: https://doi.org/10.1016/j.gaitpost.2008.10.057
4. Barton CJ, Bonanno D, Menz HB. Development and evaluation of a tool for the assessment of footwear characteristics. Journal of Foot Ankle Research. 2009;2(10). doi: 10.1186/1757-1146-2-10
5. Shakoor N, Block JA. Walking Barefoot Decreases Loading on the Lower Extremity Joints in Knee Osteoarthritis. ARTHRITIS & RHEUMATISM. 2006;54(9):2923-2927. doi: 10.1002/art.22123
6. Keenan GS, Franz JR, Dicharry J, Della Croce U, Casey Kerrigan D. Lower limb joint kinetics in walking: The role of industry recommended footwear. Gait & Posture. 2011;33(3):350-355. doi: 10.1016/j.gaitpost.2010.09.019

7. Luximon Y, Cong Y, Luximon A, Zhang M. Effects of heel base size, walking speed, and slope angle on center of pressure trajectory and plantar pressure when wearing high-heeled shoes. Human Movement Science. 2015;41;307-319. doi: 10.1016/j.humov.2015.04.003
8. Zeng Z, Liu Y, Hu X, Li P, Wang L. Effects of high-heeled shoes on lower extremity biomechanics and balance in females: a systematic review and meta-analysis. BMC Public Health. 2023;23(726). doi: 10.1186/s12889-023-15641-8
9. Barkema DD, Derrick TR, Martin PE. Heel height affects lower extremity frontal plane joint moments during walking. Gait & Posture. 2012;35(3):483-488. doi: 10.1016/j.gaitpost.2011.11.013
10. Xavier MJ, Lambert B, Jenkins GP, McGwin Jr G. Epidemiology of High-Heel Shoe Injuries in U.S. Women: 2002 to 2012. ORIGINAL RESEARCH. 2015;54(4):615-619. doi: 10.1053/j.jfas.2015.04.008
11. Cronin NJ. The effects of high heeled shoes on female gait: A review. Journal of Electromyography and Kinesiology. 2014;24(2):258-263. doi:10.1016/j.jelekin.2014.01.004
12. Alkjaer T, Raffalt P, Petersen NC, Simonsen EB. Movement Behavior of High-Heeled Walking: How Does the Nervous System Control the Ankle Joint during an Unstable Walking Condition? PLoS ONE. 2012;7(5). doi: https://doi.org/10.1371/journal.pone.0037390

13. Falola JM, Koussihouèdé FEN, Falola SMD, Avossevou YG, Bio NI, Dansou HP. Effect of the Base of the Shoe Heel on Postural Stability During Walking in Women. American Journal of BioScience. 2015;3(5):167-170. doi: 10.11648/j.ajbio.20150305.11

14. Annoni I, Mapelli A, Sidequersky FV, Zago M, Sforza C. The effect of high-heeled shoes on overground gait kinematics in young healthy women. Sport Sciences for Health. 2014;10(2):149-157. doi: 10.1007/s11332-014-0191-z

15. Sloot LH, Van der Krogt MM, Harlaar J. Effects of adding a virtual reality environment to different modes of treadmill walking. Gait & Posture. 2014;39(3): 939-945. doi: https://doi.org/10.1016/j.gaitpost.2013.12.005

16. White SC, Yack HJ, Tucker CA, Lin HY. Comparison of vertical ground reaction forces during overground and treadmill walking. Medicine & Science in Sports & Exercise. 1998;30(10): 1537–1542. doi:10.1097/00005768-199810000-00011

17. Stolze H, Kuhtz-Buschbeck JP, Mondwurf C, Boczek-Funcke A, Jöhnk K, Deuschl G, Illert M. Gait analysis during treadmill and overground locomotion in children and adults. Electroencephalography and Clinical Neurophysiology. 1997;105(6): 490–497. doi:10.1016/s0924-980x(97)00055-6

18. Menz HB, Bonanno DR. Footwear comfort: a systematic search and narrative synthesis of the literature. Journal of Foot and Ankle Research. 2021;14:63. doi:https://doi.org/10.1186/s13047-021-00500-9

19. O’Rourke B, Walsh ME, Brophy R, Vallely S, Murphy N, Conroy B, et al. Does the shoe really fit? Characterising ill-fitting footwear among community-dwelling older adults attending geriatric services: an observational cross-sectional study. BMC Geriatrics. 2020; 20: 55. doi:https://doi.org/10.1186/s12877-020-1448-9

20. Aminian K, Renzakhanlou K, De Andres E, Fritsch C, Leyvraz PF, Robert P. Temporal feature estimation during walking using miniature accelerometers: an analysis of gait improvement after hip arthroplasty. Medical & Biological Engineering & Computing. 1999;37(6):686-691. doi: 10.1007/BF02513368

21. Jasiewicz Jan M, Allum John HJ, Middleton James W, Barriskill Andrew, Condie Peter, Pucell Brendan, et al. Gait event detection using linear accelerometers or angular velocity transducers in able-bodied and spinal-cord injured individuals. Gait & Posture. 2006;24(4):502-509. doi: https://doi.org/10.1016/j.gaitpost.2005.12.017

22. Anwary AR, Yu H, Vassallo M. Optimal Foot Location for Placing Wearable IMU Sensors and Automatic Feature Extraction for Gait Analysis. IEEE Sensors Journal. 2018;18(6):2555-2567. doi: 10.1109/JSEN.2017.2786587

23. Yamamoto A, Fujita K, Yamada E, Ibara T, Nihey F, Inai T, et al. Foot characteristics of the daily-life gait in postmenopausal females with distal radius fractures: a cross-sectional study. BMC Musculoskeletal Disorders. 2023;24(1). doi: 10.1186/s12891-023-06845-5

24. Chard A, Greene A, Hunt A, Vanwanseele B, Smith R. Effect of thong style flip-flops on children's barefoot walking and jogging kinematics. Journal of Foot and Ankle Research. 2013;6:8. doi:https://doi.org/10.1186/1757-1146-6-8

25. Fukushi K, Huang C, Wang Z, Kajitani H, Nihey F, Nakahara K. On-line algorithms of stride-parameter estimation for in-shoe motion-sensor system. IEEE Sensors Journal. 2022;22:9636–9648. doi:https://doi.org/10.1109/JSEN.2022.3164057

26. Huang C, Fukushi K, Wang Z, Nihey F, Kajitani H, Nakahara K. An algorithm for real time minimum toe clearance estimation from signal of in-shoe motion sensor. Annual International Conference of the IEEE Engineering in Medicine and Biology Society. 2021: 6775–6778. doi:https://doi.org/10.1109/EMBC46164.2021.9629875

27. Huang C, Fukushi K, Wang Z, Nihey F, Kajitani H, Nakahara K. Method for estimating temporal gait parameters concerning bilateral lower limbs of healthy subjects using a single in-shoe motion sensor through a gait event detection approach. Sensors (Basel). 2022;22(1):351. doi:https://doi.org/10.3390/S22010351

28. Esenyel M, Walsh K, Walden JG, Gitter A. Kinetics of high-heeled gait. Journal of the American Podiatric Medical Association. 2003;93(1):27-32. doi: 10.7547/87507315-93-1-27

29. Lee CM, Jeong EH, Freivalds A. Biomechanical effects of wearing high-heeled shoes. International Journal of Industrial Ergonomics. 2001;28(6): 321–326. doi:https://doi.org/10.1016/S0169-8141(01)00038-5

30. Ebbeling CJ, Hamill J, Crussemeyer JA. Lower extremity mechanics and energy cost of walking in high-heeled shoes. Journal of Orthopaedic & Sports Physical Therapy. 1994;19(4): 190–196. doi:https://doi.org/10.2519/jospt.1994.19.4.190

31. Curran SA, Holliday JL, Watkeys L. Influence of high-heeled footwear and pre-fabricated orthoses on energy efficiency in ambulation. The Foot and Ankle Online Journal. 2010;3(3): 1–11. doi:https://doi.org/10.3827/faoj.2010.0303.0001

32. Komi PV. Stretch-shortening cycle: a powerful model to study normal and fatigued muscle. Journal of Biomechanics. 2000;33(10): 1197–1206. doi:https://doi.org/10.1016/S0021-9290(00)00064-6

33. Roberts TJ, Azizi E. Flexible mechanisms: the diverse roles of biological springs in vertebrate movement. Journal of Experimental Biology. 2011;214(3): 353–361. doi:https://doi.org/10.1242/jeb.038588

34. Fickey SN, Browne MG, Franz JR. Biomechanical effects of augmented ankle power output during human walking. Journal of Experimental Biology. 2018;221(22): jeb182113. doi:https://doi.org/10.1242/jeb.182113

35. Neptune RR, Clark DJ, Kautz SA. Modular control of human walking: A simulation study. Journal of Biomechanics. 2009;42(9): 1282–1287. doi:https://doi.org/10.1016/j.jbiomech.2009.03.009

36. Meinders M, Gitter A, Czerniecki JM. 1998; The role of ankle plantar flexor muscle work during walking. Scandinavian Journal of Rehabilitation Medicine. 30(1): 39–46. doi:https://doi.org/10.1080/003655098444309

37. Cinto F, Nacusse M, Haimovich H. Making a double pendulum walk: from compass- to whip-like gait. 2022 IEEE Biennial Congress of Argentina (ARGENCON). 2022;pp.1–8. doi:https://doi.org/10.1109/ARGENCON55245.2022.9939939

38. Snow RE, Williams KR. High Heeled Shoes: Their Effect on Center of Mass Position, Posture, Three-Dimensional Kinematics, Rearfoot Motion, and Ground Reaction Forces. Archives of Physical Medicine and Rehabilitation. 1994;75(5):568-576. doi: https://doi.org/10.1016/S0003-9993(21)01622-1

39. Opila KA. Kinematics of high-heeled gait. Archives of Physical Medicine and Rehabilitation. 1990;71(5):304-309. doi: 10.7547/87507315-93-1-27

40. Gehlsen G, Braarz JS, Assmann N. Effects of heel height on knee rotation and gait. Human Movement Science. 1986;5(2):149-155. doi: https://doi.org/10.1016/0167-9457(86)90021-7

41. Dadashi F, Mariani B, Rochat S, Bula CJ, Santos-Eggiman, B, Aminian K. Gait and Foot Clearance Parameters Obtained Using Shoe-Worn Inertial Sensors in a Large-Population Sample of Older Adults. Sensors(Basel). 2014;14(1):443-457. doi: 10.3390/s140100443

42. Teichtahl AJ, Morrie ME, Wluka AE, Baker R, Wolfe R, Davis SR, et al. Foot rotation-a potential target to modify the knee adduction moment. Journal of Science and Medicine in Sport. 2006;9(1-2):67-71. doi: https://doi.org/10.1016/j.jsams.2006.03.011

43. Chang A, Hurwitz D, Dunlop D, Song J, Cahue S, Hayes K, et al. The relationship between toe-out angle during gait and progression of medial tibiofemoral osteoarthritis. Annals of the Rheumatic Diseases. 2007;66(10):1271-1275. doi: 10.1136/ard.2006.062927

Click here to access/download
Supporting Information
Raw Data for Repository Ver2.xlsx

The Effects of High-Heeled Shoes on Gait Parameters in Healthy Adult Women.

Short Title: The effects of high-heeled shoes on gait parameters

Kyoma Tanigawa[a], Hiroki Shimizu[a], Anuradhi Bandara[a], Toyota Misa[a], Shota Suzuki[a], Momoko Nagai-Tanima[a], Tomoki Aoyama*[a]

[a]Department of Physical Therapy, Human Health Sciences, Graduate School of Medicine, Kyoto University, Kyoto, 606-8507, Japan

*Corresponding author: Tomoki Aoyama

E-mail: aoyama.tomoki.4e@kyoto-u.ac.jp

Abstract

Background

Walking is essential in daily life, and footwear type significantly affects walking patterns. High-heeled shoes increase the risk of knee osteoarthritis and falls in women. Traditional studies often use treadmills or unfamiliar footwear, which may not reflect daily walking. This study investigates the impact of high-heeled shoes on walking parameters in healthy adult women using in-shoe motion sensors.

Methods

Seventeen healthy adult women without pain during walking participated. They walked for 6 minutes along a 30-meter corridor wearing high-heeled shoes and sneakers. Walking data were recorded using an in-shoe motion sensor system every 2 minutes. The average of three valid consecutive steps was calculated automatically. Statistical analysis compared the mean walking parameters between the high-heel and sneaker groups.

Results

The high-heel group showed significantly lower foot clearance, stride length, peak plantar angle in dorsiflexion, and peak plantar angle in plantarflexion, and significantly higher toe-out angle in spatial parameters. Spatiotemporal parameters revealed significantly lower walking speed and maximum swing

phase speed in the high-heel group. No significant differences were observed in temporal parameters between the groups.

Conclusions

~~This study provides insights into injury prevention related to high-heeled shoes in healthy adult women, contributing to future research on daily walking evaluations. Future studies should include diverse participants and measurement devices that assess hip and knee joint movements and neurological responses to evaluate the impact of high-heeled shoes comprehensively. Additionally, understanding the biomechanical and neurological effects of different footwear can guide the development of safer shoe designs for daily use.~~

Since this study collected gait data under conditions similar to daily life, it provides data suitable for practical applications and may contribute to future research evaluating everyday gait. Additionally, future studies should include a broader range of participants and incorporate measurement devices capable of capturing hip and knee joint movements, providing a more comprehensive evaluation of the effects of high-heeled shoes on gait in healthy adult women.

Introduction

Walking is a crucial means of transporting the body safely and efficiently [1]. Additionally, it has beneficial effects on both physical and mental health, enhances human quality of life (QOL), and plays a significant role in the prevention and treatment of various diseases [2]. As such, walking is

indispensable in daily life. It is also known that walking is influenced by various external factors [3].

One of these external factors is footwear. Shoes come in various types and have multiple functions, including protecting the feet from external environments, assisting walking functions, and adapting to foot deformities. These functions are related to the occurrence of various conditions such as falls, osteoarthritis, and back pain [4]. Numerous studies have investigated the impact of shoes on walking parameters [5,6]. For example, research comparing walking barefoot and wearing athletic shoes by Shakoor et al. found that wearing athletic shoes decreases step length, increases the number of steps, and reduces joint angles of the lower limbs [5]. Thus, research on how shoes affect walking is of great importance [7].

One type of shoe that warrants attention is high-heeled shoes. High-heeled shoes are characterized by heels higher than the forefoot, narrow toe boxes, and curved soles [8]. High-heeled shoes have been widely worn by women worldwide for centuries, with 37% to 69% of women wearing them regularly and for an average of 1 to 8 hours per day [8]. Despite their regular use, high-heeled shoes are associated with medical conditions, such as osteoarthritis, and can cause frequent falls [9]. The incidence of such injuries doubled from 7.1% to 14.2% between 2002 and 2012 [10]. Therefore, it is crucial to investigate the impact of high-heeled shoes on the walking parameters of healthy adult women [7]. Several previous studies have examined this effect

[9,11,12,13]. For instance, it has been shown that wearing high-heeled shoes does not change the temporal walking parameters such as stance time and cadence [11]. Additionally, a study by Barkema et al. noted that the higher the heel height, the greater the maximum knee adduction moment, indicating a relationship between heel height and knee osteoarthritis [9].

~~Although the impact of high-heeled shoes on walking parameters has been investigated, most evaluations are conducted using treadmill walking at constant speeds. Additionally, participants often use prescribed shoes they are not accustomed to, making the conditions far from those of daily life [12]. Such conditions can alter individual walking styles in response to footwear and may not accurately reflect everyday walking [11].~~ Research on the effects of high-heeled shoes on gait parameters has significantly progressed. However, many evaluations have been conducted using a treadmill with participants walking at a constant speed [14]. Treadmill walking occurs under flat, steady-speed conditions, which differs from everyday walking environments [15]. For example, because a treadmill maintains a fixed speed, participants cannot walk at their natural pace, potentially suppressing their typical gait patterns [16,17]. Additionally, on a treadmill, individuals often need to adjust their posture and gait mechanics to match the treadmill's motion in order to maintain visual and balance cues, which is known to influence gait biomechanics [15]. In certain studies, participants wore unfamiliar shoes that may not accurately reflect their usual gait patterns in daily life [11,14]. Shoes that are unfamiliar or uncomfortable for an individual often do not fit well

with the person's foot shape or gait style, potentially affecting the gait biomechanics [18]. Specifically, when the shoe's size or shape does not align with the foot, it may cause foot pain and fatigue, while also reducing stability and increasing the risk of falls during walking [19]. Therefore, conditions different from those in daily life may alter an individual's natural gait patterns and may not accurately reflect habitual walking [11].

As a more suitable evaluation method, Inertial Measurement Units (IMUs) can be used. IMUs can estimate three-dimensional movement from angular velocity and acceleration ~~[13,14]~~[20,21]. Due to their low cost, small size, and ease of use, they have been widely used to evaluate daily life walking in recent years ~~[15]~~[22]. By using the In-Shoe Motion Sensor system (IMS), which measures the acceleration and angular velocity of the feet using IMUs and calculates walking parameters, it is possible to obtain walking data comparable to daily life more accurately than conventional studies ~~[16]~~[23].

~~Thus, the purpose of this study is to elucidate the impact of high-heeled shoes on the walking parameters of healthy adult women using IMS.~~ Therefore, this study aimed to evaluate the impact of high-heeled shoes on gait parameters in healthy adult women using the IMS system. Unlike previous studies that typically used treadmill walking at a constant speed or required participants to wear unfamiliar shoes, this study allowed participants to walk in their own footwear that fitted their feet and that they were accustomed to, under conditions similar to those of daily life. This approach

enabled us to capture a more natural gait pattern, closely resembling everyday walking, and to more accurately reflect the effects of high-heeled shoes on gait parameters.

Materials and Methods

Study design

This was an intervention study.

Participants

The participants in this study were healthy adult women enrolled at Kyoto University. The inclusion criteria were individuals aged 18 years and older who could walk for at least 6 minutes without rest. To minimize the influence of unfamiliarity with high-heeled shoes on the results, we selected participants with extensive experience wearing high heels in various settings, such as work, social events, shopping, or formal occasions, based on previous studies [14]. The exclusion criteria were individuals with neurological or orthopedic conditions that could affect walking function. The purpose of the study was thoroughly explained to the participants, and their consent was obtained prior to measurement. The sample size for this study was calculated using G Power 3.1 (Heinrich Heine University). Based on prior research [24], with an assumed effect size of 0.62, an alpha level of 0.05, and a statistical power of 0.8, the minimum required number of participants was calculated to be 15. To account for potential data loss or incomplete data, an additional five participants were included, setting the final target number of participants at 20.

Ethics statement

This study was approved by the Kyoto University Medical Ethics Committee (Approval number: R3664-3). Prior to study initiation, written informed consent was obtained from all participants in accordance with the ethical standards and guidelines.

Equipment

The IMS System (A-RROWG, NEC Corporation, Tokyo, Japan) was used for the measurements. The IMS is a device used for gait analysis and consists of key components including an ARM Cortex-M4F MCU (nRF52832 by Nordic Semiconductor). This component features a 64 MHz CPU, 64 KB of RAM, and 512 KB of ROM. Additionally, it includes a Bosch IMU (BMI160) for precise motion tracking, an ABLIC EEPROM (S-24C32C, 32K-bit) for storage, and an Epson real-time clock (RX8130CE). ~~The IMS records the average walking data when it detects the start of walking and successfully measures three steps within 9 seconds. If fewer than three steps are measured, the average is not recorded. This measurement process is conducted up to three times per minute. If all three attempts fail, the measurement for that period is considered unsuccessful, and no data is recorded. The system detects the walking cycle during level walking, measuring once every two minutes, and calculates the average of three consecutive valid walks for both the left and right~~

sides. This device performs gait measurements at 2-minute intervals, capturing gait parameters on both sides. The system detects the gait cycle during flat walking, operates for 1 min, and then enters sleep mode to conserve energy. During this 1-minute period, it detects vibrations and identifies stable walking patterns, and upon detecting the start of a gait cycle, it measures the parameters for three steps within the following 9 s. These steps do not need to be consecutive; if the three steps are successfully measured within a specified time, the average value is recorded. If fewer than three steps were measured, the average value was not recorded. Additionally, this measurement process can be repeated up to three times within a 1-minute window; if all three attempts fail, the measurement for that period is considered unsuccessful, and no data is recorded [25,26,27].

The following 16 parameters are calculated: Stride length, Maximum (peak) dorsiflexion angle, Maximum (peak) plantar flexion angle, Foot clearance, Foot height, Circumduction, Toe-in/out angle, Roll angle of heel contact, Roll angle of toe-off, Walking speed, Peak swing angular velocity, Maximum speed during swinging phase, Stance time, Swing time, Pushing time, and Cadence. Table 1 below, disclosed by NEC Corporation, categorizes the definitions of each parameter into spatial, spatiotemporal, and temporal parameters. These parameters were validated in previous studies for accuracy or were derived from validated parameters [25,26,27].

For the shoes compared to high-heeled shoes, the participants' daily sneakers were used. The definition of each type of shoe was based on the "model number tracked to the purchase source,

with the manufacturing company selling the shoes as high heels or sneakers." High-heeled shoes were defined as “footwear with a heel elevated higher than the forefoot, a narrow toe box, and a curved sole” [11], while sneakers were defined as “light footwear that has a top made of cloth and a bottom made of rubber.”

Table 1. Definitions of Each Walking Parameter.

Classification	Parameters	Definition
**Spatial parameters**	Stride length (cm)	Distance from the starting point to end point of the foot trajectory
	Maximum (peak) dorsiflexion angle (degree)	Peak foot-sole angle in dorsiflexion
	Maximum (peak) plantar flexion angle (degree)	Peak foot-sole angle in plantarflexion
	Foot clearance (cm)	Maximum heel height of the foot trajectory
	Foot height (cm)	Maximum vertical height of the foot trajectory
	Circumduction (cm)	Displacement in the medial–lateral direction during the swing phase

	Toe-in/out angle (degree)	Average adduction/abduction angle during the swing phase
	Roll angle of heel contact (degree)	Plantar roll angle when the heel touches the ground
	Roll angle of toe-off (degree)	Plantar roll angle when the toe leaves the ground
**Spatiotemporal parameters**	Walking speed (km/h)	Stride length divided by stride time
	Peak swing angular velocity (degree/s)	Peak angular velocity of the foot during the swing phase
	Maximum speed during swinging phase (km/h)	Maximum forward speed of the swinging leg during the swinging phase
**Temporal parameters**	Stance time(s)	Amount of time that the foot is on the ground during the gait cycle
	Swing time(s)	Amount of time that the foot is off the ground during the gait cycle

	Pushing time (s)	Time between the heel contact and toe leaving the ground.
	Cadence (steps/min)	Number of steps per min

Procedure

The measurements were conducted in a 30-meter corridor at the Faculty of Human Health Sciences building, Kyoto University. First, we verified whether the shoes worn by the participants met the aforementioned criteria. Subsequently, we measured the age (years), height (cm), ~~weight~~ body mass(kg), size of the shoes(cm), heel height (cm) of the high-heeled shoes, and ~~weight~~ mass (g) of each shoe. Prior to data collection, participants walked 5 m with insoles equipped with an IMS inserted into their shoes to ensure that they experienced no discomfort during walking. ~~The measurer stood at the center of the path during measurements to confirm that participants walked normally and safely. Additionally, the order of wearing high-heeled shoes and sneakers was determined using Excel's random function. Participants were instructed to walk at their usual pace, starting from one end of the corridor, turning upon reaching the other end, and continuing for 6 minutes.~~ For data collection, Participants placed a smartphone, used for data recording, in either pocket of their pants, as there were no restrictions on which side to use. They were instructed to begin walking from one end of the corridor, turn around at the opposite end, and continue this back-

and-forth walking for 6 min. The measurements were conducted twice: once in sneakers and once in high-heeled shoes, with the order of conditions determined using Excel's random function. Participants were given the option to take a break between trials upon request. The observer stood in the middle of the pathway during the test to ensure that the participants walked normally and without safety issues, providing updates on the remaining time every minute. If no data were collected, the 6-minute walking test was repeated.

Data analysis

Statistical analyses were performed using the JMP PRO16 software (SAS Institute). ~~Shapiro-Wilk tests were conducted to assess normality of distributions for comparisons between the heel group and sneaker group. For data showing normal distribution, paired t-tests were employed. For data not showing normal distribution, Wilcoxon signed-rank tests were used.~~ First, the Shapiro-Wilk test was conducted to determine whether each dataset followed a normal distribution. For data that were normally distributed, a paired t-test was used, because the same participants were measured under different conditions (high-heeled shoes and sneakers). For data that did not follow a normal distribution, the Wilcoxon signed-rank test, suitable for paired datasets, was applied. The group wearing high-heeled shoes was referred to as the "high-heeled group" (HG) and the group wearing sneakers as the "sneaker group" (SG). Data are presented as mean standard deviation ±

standard deviation(SD). The effect size was evaluated using Cohen’s d to assess the magnitude of the differences between groups. The statistical significance level for this study was set at less than 5%.

Results

A. Physical Characteristics (Table 2)

There were 17 participants in the study, with no dropouts or adverse events reported. The mean values were as follows: age, 23.4 years; height, 161.78 cm; ~~weight~~ body mass, 53.46 kg; BMI, 20.38; size of shoes, 24.15 cm; ~~weight~~ mass of high-heeled shoes, 201.76 g; ~~weight~~ mass of sneakers, 265.00 g; heel height of high-heeled shoes, 4.93 cm (Table1).

Table 2. Basic Information of Participants.

Characteristics	Mean±SD(N=17)
Age(years)	23.40±1.23
Height(cm)	161.78±6.14
~~Weight~~ Body Mass(kg)	53.46±5.61
BMI(kg/m^2)	20.38±1.09
Size of shoes(cm)	24.15±1.01
~~Weight~~ Mass of heel shoes(g)	201.76±37.12

~~Weight~~ Mass of sneakers(g)	265.00±59.48
Heel height of heel shoes(cm)	4.93±1.17

SD, standard deviation; BMI, body mass index

B. Comparison of Walking Parameters Between High-Heeled and Sneaker Shoes (Table 3)

From the table 3, in the comparison between the high-heeled group and the sneaker group, spatial parameters showed that the high-heeled group had a significantly shorter stride length, lower peak dorsiflexion angle of the foot, lower peak plantar flexion angle of the foot, and significantly higher toe angle compared to the sneaker group. Regarding spatiotemporal parameters, the high-heeled group exhibited significantly lower walking speed and maximum speed during swinging phase. There were no significant differences observed between the groups in temporal parameters.

Table 3. Comparisons of the mean values of each parameter in each category.

Classification	Parameters	HG	SG	P-value	Effect Size (Cohen's d)

	Stride length (cm)	130.42±18.14	143.88±16.09	0.0006*	-1.09
	Maximum (peak) dorsiflexion angle (degree)	24.21±5.25	29.88±5.13	0.0017*	-0.94
	Maximum (peak) plantar flexion angle (degree)	62.33±11.15	78.31±6.49	<0.0001*	-1.47
**Spatial parameters**	Foot clearance (cm)	21.17±3.31	24.05±2.15	0.0005*	-1.09
	Foot height (cm)	13.33±2.40	15.70±1.76	0.0023*	-0.90
	Circumduction (cm)	4.10±1.58	3.72±2.01	0.5397	0.15
	Toe-in/out angle (degree)	12.05±8.82	10.14±7.97	0.0476*	0.54
	Roll angle of heel contact (degree)	5.21±4.86	3.71±5.70	0.2343	0.30
	Roll angle of toe-off (degree)	-2.05±6.17	-1.36±4.52	0.5178	-0.17
**Spatiotemporal parameters**	Walking speed (km/h)	4.61±0.68	5.08±0.66	0.0005*	-1.09

	Peak swing angular velocity (degree/s)	531.64±95.96	565.64±75.54	0.0932	-0.45
	Maximum speed during swinging phase (km/h)	15.15±1.65	15.97±1.59	0.0085*	-0.75
**Temporal parameters**	Stance time(s)	0.62±0.05	0.62±0.04	0.8271	-0.06
	Swing time(s)	0.41±0.04	0.40±0.01	0.3225	0.03
	Pushing time (s)	0.19±0.03	0.20±0.02	0.6805	-0.13
	Cadence (steps/min)	117.22±6.41	116.92±5.69	0.8030	0.06

SD, standard deviation. *p<0.05.

Effect size (Cohen's d): Small = 0.2–0.49, Medium = 0.5–0.79, Large ≥ 0.8

Discussion

~~The purpose of this study was to investigate the effects of high-heeled shoes on walking parameters in healthy adult women using IMS.~~ This study aimed to evaluate the effect of high-heeled shoes on gait parameters in healthy adult women using an IMS system. In previous studies, participants often wore unfamiliar shoes and measurements were commonly conducted on a

treadmill at a constant speed, which may not accurately reflect natural gait patterns in real-life settings. In contrast, this study utilized a compact and user-friendly IMS to collect gait data while participants wore their own familiar shoes. This approach allowed for accurate collection in a manner closer to daily life conditions, providing foundational data for assessing the effects of footwear under more realistic circumstances.

~~As a result, in terms of spatial parameters, the high-heeled group showed significantly shorter stride length, lower maximum dorsiflexion angle, lower maximum plantar flexion angle, and significantly higher toe-out angle compared to the sneaker group. Additionally, in spatiotemporal parameters, the high-heeled group exhibited significantly lower walking speed and maximum speed during swinging phase. However, no significant differences were observed between the groups in temporal parameters.~~ As a result, in terms of spatial parameters, the high-heeled group showed significantly shorter stride length, lower maximum dorsiflexion angle, lower maximum plantar flexion angle, and significantly higher toe-out angle compared to the sneaker group, with effect sizes ranging from moderate to high. Additionally, in spatiotemporal parameters, the high-heeled group exhibited significantly lower walking speed and maximum speed during the swinging phase, with large effect sizes. However, no significant differences were observed between the groups in temporal parameters.

~~Previous studies have shown that wearing high-heeled shoes can results in shorter lengths of the gastrocnemius muscle and Achilles tendon due to the proximity of ground reaction forces to the ankle joint and the curved shape of the sole, causing the ankle joint to be in a plantarflexed position. [11]. Consequently, it has been suggested that during high-heeled shoe use, the ankle plantarflexion moment decreases, indicating weaker propulsive force from the foot compared to sneakers or barefoot conditions [17]. Similarly, in this study, it is plausible that the high-heeled group exhibited significantly lower maximum walking speed, maximum speed during swinging phase, and stride length for similar reasons.~~ Regarding spatial parameters such as walking speed, stride length, and maximum swing speed, it is generally known that wearing high-heeled shoes reduces the base of support during walking, prompting individuals to adopt a safer and more stable gait pattern [28,29]. Additionally, to minimize increases in energy expenditure, they tend to decrease their walking speed and stride length as a result [30,31]. In addition to these stable and efficient walking strategies, previous studies have shown that wearing high heels brings the ground reaction force closer to the ankle joint, and the curved shape of the shoe sole causes the ankle to remain in a plantarflexed position [11]. These factors can shorten the length of the triceps surae muscle and Achilles tendons. Consequently, compared to sneakers or barefoot walking, ankle plantarflexion moments decrease, and the effect of the stretch-shortening cycle on the muscle-tendon complex is reduced [32,33]. The stretch-shortening cycle is a process in which muscles and tendons quickly

stretch and then shorten, store, and release elastic energy, and plays a crucial role in efficient force production [33]. However, the shortened state of the muscle-tendon complex induced by wearing high heels is thought to impede this function, leading to decreased energy efficiency and a weaker push-off force. As a result, the calculated parameters were significantly different between the two groups.

Similarly, foot clearance and foot height were significantly lower in the high-heeled group. ~~Previous studies comparing kinematics such as lower limb sagittal plane joint angles with varying heel heights during steady walking speeds have shown that hip flexion angles during the swing phase do not change with heel height, while knee flexion angles significantly decrease as heel height increases [18,19,20]. In this study, it is possible that the high-heeled group exhibited decreased knee flexion angles during the swing phase, resulting in relatively lower positions of the lower leg and decreased foot clearance. Decreased foot clearance increases the risk of falling [21].~~ As noted above, wearing high-heeled shoes is suggested to reduce the push-off force of the ankle. Additionally, previous studies investigating the role of the ankle in walking have shown that ankle push-off contributes to up to 50% of the power needed for leg swinging during the terminal stance and early swing phases [34,35,36]. This reduction in ankle push-off may decrease the leg swing speed, which is essential for the double-pendulum effect during walking [37]. Consequently, a lack of sufficient knee flexion in the double-pendulum mechanism may cause the lower leg to remain

relatively low, potentially resulting in reduced foot clearance. In fact, prior research comparing joint angles in the sagittal plane under different heel heights at a constant walking speed found that while hip flexion during the swing phase remained unaffected by heel height, the knee flexion angle significantly decreased as heel height increased [38,39,40]. Moreover, a decrease in the foot clearance height can increase the risk of tripping over small obstacles or uneven surfaces while walking, potentially leading to falls [41]. High-heeled shoes also tilt the toes downward and elevate the heel, reducing the contact area of the foot with the ground, which may further destabilize balance. This combination of reduced foot clearance and a limited base of support likely exacerbates the risk of falls [14].

Thus, the high-heeled group showed significantly lower stride length, lower peak dorsiflexion angle of the foot, lower peak plantar flexion angle of the foot, lower walking speed, and swing phase maximum velocity, while the toe angle during the swing phase was significantly higher. A higher toe angle indicates a larger ankle ~~eversion~~ abduction angle. Studies investigating the influence of heel height on the anterior-posterior joint moments of the lower limb have shown that as heel height increases, knee internal rotation moments increase, potentially increasing the load on the medial compartment of the knee [9,~~22~~42]. Conversely, walking with toes pointing outward reduces knee internal rotation moments and decreases the load on the medial compartment [~~23~~43]. In this study, it is suggested that the high-heeled group adopted a walking strategy with toes

pointing outward to reduce the load on the medial compartment of the knee. Additionally, since knee joint movements during stance and toe direction were not measured in this study, further experiments are necessary.

~~In conclusion, while significant differences were observed in spatial/spatiotemporal parameters, similar to previous studies, no significant differences were found in temporal parameters [24]. Previous studies have shown that cadence during inclined and level walking remains unchanged [25]. Furthermore, Alkjaer et al. demonstrated that walking in high heels requires adaptation of a different neural control system compared to normal walking [26]. In this study, it is suggested that the use of a similar neural control system led to changes in spatial/spatiotemporal parameters of the lower limb, while temporal parameters remained unchanged.~~

~~Two main limitations of this study can be identified. First, the study was limited to healthy adult women, which may restrict the generalizability of the findings to other demographic groups. Second, measurements of joint movements and neurological responses, aside from ankle joints such as hip and knee joints, were not conducted. Therefore, the impact of these factors on the results remains unclear. Future research should aim to expand participant demographics and utilize devices capable of measuring movements of joints other than the ankle, as well as neurological responses concurrently with IMS. This comprehensive approach is necessary to more thoroughly evaluate the effects of different types of footwear on walking.~~

There are limitations in this study; the participants were limited to healthy adult women from the same university, which restricts the generalizability of our findings. To apply these results to other age groups, sexes, or individuals with specific health conditions, further studies with more diverse participants are necessary. Second, we were unable to capture the movements of the hip and knee joints, making it challenging to fully assess how these joints affect gait mechanics, particularly balance and posture control. Specifically, it remains unclear to what extent high-heeled shoes stress on these joints under near-daily conditions and how this stress might increase the risk of falls or lead to long-term joint disorders. Future research should incorporate devices that measure the movements of these joints to provide a more comprehensive evaluation of the impact of different footwear types on gait and balance.

Conclusions

This study investigated the effects of high-heeled shoes on walking parameters in healthy adult women using the IMS. As a result, in terms of spatial parameters, the high-heeled group showed significantly shorter stride length, lower maximum dorsiflexion angle, lower maximum plantar flexion angle, and significantly higher toe-out angle compared to the sneaker group, with effect sizes ranging from moderate to high. Additionally, in spatiotemporal parameters, the high-heeled group exhibited significantly lower walking speed and maximum speed during the swinging phase,

with large effect sizes. However, no significant differences were observed between the groups in temporal parameters.

~~Moving forward, it is essential to evaluate the impact of high-heeled shoes on daily walking more comprehensively using experimental devices that can measure hip and knee joint movements, as well as neurological responses concurrently with IMS.~~

~~This study provides new insights into injury prevention related to high-heeled shoe use in healthy adult women and can contribute to future research evaluating everyday walking.~~ These findings suggest that wearing high-heeled shoes has a substantial impact on gait stability and efficiency, potentially increasing the risk of falls and musculoskeletal strain. Future studies should include a more diverse population, such as older adults or individuals with joint disorders, and incorporate motion capture systems or wearable sensors that can measure lower limb joint dynamics. These additional studies would allow for a comprehensive understanding of how footwear affects not only the ankle but also hip and knee joint movements across a wider population. Because this study collected gait data under conditions similar to those of daily life, it provides data suitable for practical applications and may contribute to future research focused on evaluating gait in everyday scenarios.

Acknowledgments

We express our gratitude to all participants, instructors, and laboratory members for their cooperation and valuable input throughout this study. We also thank Editage (www.editage.com) for English editing.

References

1. Prince F, Corriveau H., Hebert R, Winter DA. Gait in elderly. Gait & Posture. 1997;5(2): 128-135. doi: 10.1016/S0966-6362(97)01118-1
2. Bai X, Kim GS, Dev RDO, Talib O, Xiao W, Cai H. Effect of Brisk Walking on Health-Related Physical Fitness Balance and Life Satisfaction Among the Elderly: A Systematic Review. Frontiers in Public Health. 2022;9. doi: https://doi.org/10.3389/fpubh.2021.829367
3. Menant JC, Steele JR, Menz HB, Munro BJ, Lord SR. Effects of walking surfaces and footwear on temporo-spatial gait parameters in young and older people. Gait & Posture. 2009;29(3):392-397. doi: https://doi.org/10.1016/j.gaitpost.2008.10.057
4. Barton CJ, Bonanno D, Menz HB. Development and evaluation of a tool for the assessment of footwear characteristics. Journal of Foot Ankle Research. 2009;2(10). doi: 10.1186/1757-1146-2-10

5. Shakoor N, Block JA. Walking Barefoot Decreases Loading on the Lower Extremity Joints in Knee Osteoarthritis. ARTHRITIS & RHEUMATISM. 2006;54(9):2923-2927. doi: 10.1002/art.22123
6. Keenan GS, Franz JR, Dicharry J, Della Croce U, Casey Kerrigan D. Lower limb joint kinetics in walking: The role of industry recommended footwear. Gait & Posture. 2011;33(3):350-355. doi: 10.1016/j.gaitpost.2010.09.019
7. Luximon Y, Cong Y, Luximon A, Zhang M. Effects of heel base size, walking speed, and slope angle on center of pressure trajectory and plantar pressure when wearing high-heeled shoes. Human Movement Science. 2015;41;307-319. doi: 10.1016/j.humov.2015.04.003
8. Zeng Z, Liu Y, Hu X, Li P, Wang L. Effects of high-heeled shoes on lower extremity biomechanics and balance in females: a systematic review and meta-analysis. BMC Public Health. 2023;23(726). doi: 10.1186/s12889-023-15641-8
9. Barkema DD, Derrick TR, Martin PE. Heel height affects lower extremity frontal plane joint moments during walking. Gait & Posture. 2012;35(3):483-488. doi: 10.1016/j.gaitpost.2011.11.013
10. Xavier MJ, Lambert B, Jenkins GP, McGwin Jr G. Epidemiology of High-Heel Shoe Injuries in U.S. Women: 2002 to 2012. ORIGINAL RESEARCH. 2015;54(4):615-619. doi: 10.1053/j.jfas.2015.04.008

11. Cronin NJ. The effects of high heeled shoes on female gait: A review. Journal of Electromyography and Kinesiology. 2014;24(2):258-263. doi:10.1016/j.jelekin.2014.01.004

12. Alkjaer T, Raffalt P, Petersen NC, Simonsen EB. Movement Behavior of High-Heeled Walking: How Does the Nervous System Control the Ankle Joint during an Unstable Walking Condition? PLoS ONE. 2012;7(5). doi: https://doi.org/10.1371/journal.pone.0037390

13. Falola JM, Koussihouèdé FEN, Falola SMD, Avossevou YG, Bio NI, Dansou HP. Effect of the Base of the Shoe Heel on Postural Stability During Walking in Women. American Journal of BioScience. 2015;3(5):167-170. doi: 10.11648/j.ajbio.20150305.11

14. Annoni I, Mapelli A, Sidequersky FV, Zago M, Sforza C. The effect of high-heeled shoes on overground gait kinematics in young healthy women. Sport Sciences for Health. 2014;10(2):149-157. doi: 10.1007/s11332-014-0191-z

15. Sloot LH, Van der Krogt MM, Harlaar J. Effects of adding a virtual reality environment to different modes of treadmill walking. Gait & Posture. 2014;39(3): 939-945. doi: https://doi.org/10.1016/j.gaitpost.2013.12.005

16. White SC, Yack HJ, Tucker CA, Lin HY. Comparison of vertical ground reaction forces during overground and treadmill walking. Medicine & Science in Sports & Exercise. 1998;30(10): 1537–1542. doi:10.1097/00005768-199810000-00011

17. Stolze H, Kuhtz-Buschbeck JP, Mondwurf C, Boczek-Funcke A, Jöhnk K, Deuschl G, Illert M. Gait analysis during treadmill and overground locomotion in children and adults. Electroencephalography and Clinical Neurophysiology. 1997;105(6): 490–497. doi:10.1016/s0924-980x(97)00055-6

18. Menz HB, Bonanno DR. Footwear comfort: a systematic search and narrative synthesis of the literature. Journal of Foot and Ankle Research. 2021;14:63. doi:https://doi.org/10.1186/s13047-021-00500-9

19. O'Rourke B, Walsh ME, Brophy R, Vallely S, Murphy N, Conroy B, et al. Does the shoe really fit? Characterising ill-fitting footwear among community-dwelling older adults attending geriatric services: an observational cross-sectional study. BMC Geriatrics. 2020; 20: 55. doi:https://doi.org/10.1186/s12877-020-1448-9

20. Aminian K, Renzakhanlou K, De Andres E, Fritsch C, Leyvraz PF, Robert P. Temporal feature estimation during walking using miniature accelerometers: an analysis of gait improvement after hip arthroplasty. Medical & Biological Engineering & Computing. 1999;37(6):686-691. doi: 10.1007/BF02513368

21. Jasiewicz Jan M, Allum John HJ, Middleton James W, Barriskill Andrew, Condie Peter, Pucell Brendan, et al. Gait event detection using linear accelerometers or angular velocity

transducers in able-bodied and spinal-cord injured individuals. Gait & Posture. 2006;24(4):502-509. doi: https://doi.org/10.1016/j.gaitpost.2005.12.017

22. Anwary AR, Yu H, Vassallo M. Optimal Foot Location for Placing Wearable IMU Sensors and Automatic Feature Extraction for Gait Analysis. IEEE Sensors Journal. 2018;18(6):2555-2567. doi: 10.1109/JSEN.2017.2786587

23. Yamamoto A, Fujita K, Yamada E, Ibara T, Nihey F, Inai T, et al. Foot characteristics of the daily-life gait in postmenopausal females with distal radius fractures: a cross-sectional study. BMC Musculoskeletal Disorders. 2023;24(1). doi: 10.1186/s12891-023-06845-5

24. Chard A, Greene A, Hunt A, Vanwanseele B, Smith R. Effect of thong style flip-flops on children's barefoot walking and jogging kinematics. Journal of Foot and Ankle Research. 2013;6:8. doi:https://doi.org/10.1186/1757-1146-6-8

25. Fukushi K, Huang C, Wang Z, Kajitani H, Nihey F, Nakahara K. On-line algorithms of stride-parameter estimation for in-shoe motion-sensor system. IEEE Sensors Journal. 2022;22:9636–9648. doi:https://doi.org/10.1109/JSEN.2022.3164057

26. Huang C, Fukushi K, Wang Z, Nihey F, Kajitani H, Nakahara K. An algorithm for real time minimum toe clearance estimation from signal of in-shoe motion sensor. Annual International Conference of the IEEE Engineering in Medicine and Biology Society. 2021: 6775–6778. doi:https://doi.org/10.1109/EMBC46164.2021.9629875

27. Huang C, Fukushi K, Wang Z, Nihey F, Kajitani H, Nakahara K. Method for estimating temporal gait parameters concerning bilateral lower limbs of healthy subjects using a single in-shoe motion sensor through a gait event detection approach. Sensors (Basel). 2022;22(1):351. doi:https://doi.org/10.3390/S22010351

28. Esenyel M, Walsh K, Walden JG, Gitter A. Kinetics of high-heeled gait. Journal of the American Podiatric Medical Association. 2003;93(1):27-32. doi: 10.7547/87507315-93-1-27

29. Lee CM, Jeong EH, Freivalds A. Biomechanical effects of wearing high-heeled shoes. International Journal of Industrial Ergonomics. 2001;28(6): 321–326. doi:https://doi.org/10.1016/S0169-8141(01)00038-5

30. Ebbeling CJ, Hamill J, Crussemeyer JA. Lower extremity mechanics and energy cost of walking in high-heeled shoes. Journal of Orthopaedic & Sports Physical Therapy. 1994;19(4): 190–196. doi:https://doi.org/10.2519/jospt.1994.19.4.190

31. Curran SA, Holliday JL, Watkeys L. Influence of high-heeled footwear and pre-fabricated orthoses on energy efficiency in ambulation. The Foot and Ankle Online Journal. 2010;3(3): 1–11. doi:https://doi.org/10.3827/faoj.2010.0303.0001

32. Komi PV. Stretch-shortening cycle: a powerful model to study normal and fatigued muscle. Journal of Biomechanics. 2000;33(10): 1197–1206. doi:https://doi.org/10.1016/S0021-9290(00)00064-6

33. Roberts TJ, Azizi E. Flexible mechanisms: the diverse roles of biological springs in vertebrate movement. Journal of Experimental Biology. 2011;214(3): 353–361. doi:https://doi.org/10.1242/jeb.038588

34. Fickey SN, Browne MG, Franz JR. Biomechanical effects of augmented ankle power output during human walking. Journal of Experimental Biology. 2018;221(22): jeb182113. doi:https://doi.org/10.1242/jeb.182113

35. Neptune RR, Clark DJ, Kautz SA. Modular control of human walking: A simulation study. Journal of Biomechanics. 2009;42(9): 1282–1287. doi:https://doi.org/10.1016/j.jbiomech.2009.03.009

36. Meinders M, Gitter A, Czerniecki JM. 1998; The role of ankle plantar flexor muscle work during walking. Scandinavian Journal of Rehabilitation Medicine. 30(1): 39–46. doi:https://doi.org/10.1080/003655098444309

37. Cinto F, Nacusse M, Haimovich H. Making a double pendulum walk: from compass- to whip-like gait. 2022 IEEE Biennial Congress of Argentina (ARGENCON). 2022;pp.1–8. doi:https://doi.org/10.1109/ARGENCON55245.2022.9939939

38. Snow RE, Williams KR. High Heeled Shoes: Their Effect on Center of Mass Position, Posture, Three-Dimensional Kinematics, Rearfoot Motion, and Ground Reaction Forces. Archives of Physical Medicine and Rehabilitation. 1994;75(5):568-576.

doi: https://doi.org/10.1016/S0003-9993(21)01622-1

39. Opila KA. Kinematics of high-heeled gait. Archives of Physical Medicine and Rehabilitation. 1990;71(5):304-309. doi: 10.7547/87507315-93-1-27

40. Gehlsen G, Braarz JS, Assmann N. Effects of heel height on knee rotation and gait. Human Movement Science. 1986;5(2):149-155. doi: https://doi.org/10.1016/0167-9457(86)90021-7

41. Dadashi F, Mariani B, Rochat S, Bula CJ, Santos-Eggiman, B, Aminian K. Gait and Foot Clearance Parameters Obtained Using Shoe-Worn Inertial Sensors in a Large-Population Sample of Older Adults. Sensors(Basel). 2014;14(1):443-457. doi: 10.3390/s140100443

42. Teichtahl AJ, Morrie ME, Wluka AE, Baker R, Wolfe R, Davis SR, et al. Foot rotation-a potential target to modify the knee adduction moment. Journal of Science and Medicine in Sport. 2006;9(1-2):67-71. doi: https://doi.org/10.1016/j.jsams.2006.03.011

43. Chang A, Hurwitz D, Dunlop D, Song J, Cahue S, Hayes K, et al. The relationship between toe-out angle during gait and progression of medial tibiofemoral osteoarthritis. Annals of the Rheumatic Diseases. 2007;66(10):1271-1275. doi: 10.1136/ard.2006.062927

~~44. Adrian MJ, Karpovich PV. Foot Instability during Walking in Shoes with High Heels. Research Quarterly. American Association for Health, Physical Education and Recreation. 1966;37(2):168–175. doi: https://doi.org/10.1080/10671188.1966.10613359~~

45. Dewolf AH, Ivanenko YP, Zelik KE, Lacquaniti F, Willems PA. Differential activation of lumbar and sacral motor pools during walking at different speeds and slopes. Journal of Neurophysiology. 2019;122(2):872-887. doi: 10.1152/jn.00167.2019

46. Alkjaer T, Raffalt P, Petersen NC, Simonsen EB. Movement Behavior of High-Heeled Walking: How Does the Nervous System Control the Ankle Joint during an Unstable Walking Condition? PLoS ONE. 2012;7(5).
doi: https://doi.org/10.1371/journal.pone.0037390

Point-by-point response

We are very grateful to the editors for providing such valuable, thoughtful, and thorough comments. The comments and questions raised have prompted us to consider our data from new and interesting angles, the culmination of which we feel has greatly strengthened the work. Thank you for your time.

Editor's comments:

[Comment #1]
Please use the space provided to explain your answers to the questions above. You may also include additional comments for the author, including concerns about dual publication, research ethics, or publication ethics. (Please upload your review as an attachment if it exceeds 20,000 characters)

[Author action #1]
Thank you for your detailed review and for providing the opportunity to clarify our responses. We have addressed each of your questions thoroughly in the space provided, as well as in the manuscript itself where appropriate. We are committed to maintaining high standards of research and publication ethics, and we confirm that this study adheres to all ethical guidelines, including informed consent from participants and approval from the relevant ethics committee. We appreciate your time and consideration in reviewing our work.

Reviewer #1:
[Comment #1]
The purpose of this quasiexperimental study was to compare walking temporal spatial parameters in women wearing sneakers and high heeled shoes. The authors provided sufficient background perhaps identifying a need for this project. The manuscript was well organized, although insufficient information in the methods section would not allow this research to be replicated. The results, discussion and conclusion related to the study's primary purpose; although the conclusion needs to be expanded a bit. There were some minor errors in word choice and tense. See pdf for specific comments.

[Author action #1]

Thank you very much for your valuable feedback. As you suggested, we have expanded the methods section and conclusion, providing more detail in the methods to enhance reproducibility. In the conclusion, we clarified the implications of the results, future directions, and the significance of this study. We have also corrected errors related to word choice and tense. We would appreciate it if you could review it at your convenience. Thank you for your time and consideration.
The changed text is highlighted in yellow as follows:

Participants

The participants in this study were healthy adult women enrolled at Kyoto University. The inclusion criteria were individuals aged 18 years and older who could walk for at least 6 minutes without rest. To minimize the influence of unfamiliarity with high-heeled shoes on the results, we selected participants with extensive experience wearing high heels in various settings, such as work, social events, shopping, or formal occasions, based on previous studies [14]. The exclusion criteria were individuals with neurological or orthopedic conditions that could affect walking function. The purpose of the study was thoroughly explained to the participants, and their consent was obtained prior to measurement. The sample size for this study was calculated using G Power 3.1 (Heinrich Heine University). Based on prior research [24], with an assumed effect size of 0.62, an alpha level of 0.05, and a statistical power of 0.8, the minimum required number of participants was calculated to be 15. To account for potential data loss or incomplete data, an additional five participants were included, setting the final target number of participants at 20.

Line numbers where the changed text appears: 127-136

Equipment

The IMS System (A-RROWG, NEC Corporation, Tokyo, Japan) was used for the measurements. The IMS is a device used for gait analysis and consists of key components including an ARM Cortex-M4F MCU (nRF52832 by Nordic Semiconductor). This component features a 64 MHz CPU, 64 KB of RAM, and 512 KB of ROM. Additionally, it includes a Bosch IMU (BMI160) for precise motion tracking, an ABLIC EEPROM (S-24C32C, 32K-bit) for storage, and an Epson real-time clock (RX8130CE). This device performs gait measurements at 2-minute intervals, capturing gait parameters on both sides. The system detects the gait cycle during flat walking, operates for 1 min, and then enters sleep mode to conserve energy. During this 1-minute period, it detects vibrations and identifies stable walking patterns, and upon detecting the start of a gait cycle, it measures the parameters for three steps within the following 9 s. These steps do not need to be consecutive; if the three steps are successfully measured within a specified time, the average value is recorded. If fewer than three steps were measured, the average value was not recorded. Additionally, this measurement process can be repeated up to three times within a 1-minute window; if all three attempts fail,

the measurement for that period is considered unsuccessful, and no data is recorded [25,26,27].

The following 16 parameters are calculated: Stride length, Maximum (peak) dorsiflexion angle, Maximum (peak) plantar flexion angle, Foot clearance, Foot height, Circumduction, Toe-in/out angle, Roll angle of heel contact, Roll angle of toe-off, Walking speed, Peak swing angular velocity, Maximum speed during swinging phase, Stance time, Swing time, Pushing time, and Cadence. Table 1 below, disclosed by NEC Corporation, categorizes the definitions of each parameter into spatial, spatiotemporal, and temporal parameters. These parameters were validated in previous studies for accuracy or were derived from validated parameters [25,26,27].

High-heeled shoes were defined as “footwear with a heel elevated higher than the forefoot, a narrow toe box, and a curved sole” [11], while sneakers were defined as “light footwear that has a top made of cloth and a bottom made of rubber.”

Line numbers where the changed text appears: 149-176

Procedure

The measurements were conducted in a 30-meter corridor at the Faculty of Human Health Sciences building, Kyoto University. First, we verified whether the shoes worn by the participants met the aforementioned criteria. Subsequently, we measured the age

(years), height (cm), body mass(kg), size of the shoes(cm), heel height (cm) of the high-heeled shoes, and ~~weight~~ mass (g) of each shoe. Prior to data collection, participants walked 5 m with insoles equipped with an IMS inserted into their shoes to ensure that they experienced no discomfort during walking. For data collection, Participants placed a smartphone, used for data recording, in either pocket of their pants, as there were no restrictions on which side to use. They were instructed to begin walking from one end of the corridor, turn around at the opposite end, and continue this back-and-forth walking for 6 min. The measurements were conducted twice: once in sneakers and once in high-heeled shoes, with the order of conditions determined using Excel's random function. Participants were given the option to take a break between trials upon request. The observer stood in the middle of the pathway during the test to ensure that the participants walked normally and without safety issues, providing updates on the remaining time every minute. If no data were collected, the 6-minute walking test was repeated.

Line numbers where the changed text appears: 179-198

Data analysis

Statistical analyses were performed using the JMP PRO16 software (SAS Institute). First, the Shapiro-Wilk test was conducted to determine whether each dataset followed a

normal distribution. For data that were normally distributed, a paired t-test was used, because the same participants were measured under different conditions (high-heeled shoes and sneakers). For data that did not follow a normal distribution, the Wilcoxon signed-rank test, suitable for paired datasets, was applied. The group wearing high-heeled shoes was referred to as the "high-heeled group" (HG) and the group wearing sneakers as the "sneaker group" (SG). Data are presented as mean standard deviation ± standard deviation(SD). The effect size was evaluated using Cohen's d to assess the magnitude of the differences between groups. The statistical significance level for this study was set at less than 5%.

Line numbers where the changed text appears: 200-213

Conclusions

This study investigated the effects of high-heeled shoes on walking parameters in healthy adult women using the IMS. As a result, in terms of spatial parameters, the high-heeled group showed significantly shorter stride length, lower maximum dorsiflexion angle, lower maximum plantar flexion angle, and significantly higher toe-out angle compared to the sneaker group, with effect sizes ranging from moderate to high. Additionally, in spatiotemporal parameters, the high-heeled group exhibited significantly lower walking speed and maximum speed during the swinging phase, with

large effect sizes. However, no significant differences were observed between the groups in temporal parameters.

These findings suggest that wearing high-heeled shoes has a substantial impact on gait stability and efficiency, potentially increasing the risk of falls and musculoskeletal strain. Future studies should include a more diverse population, such as older adults or individuals with joint disorders, and incorporate motion capture systems or wearable sensors that can measure lower limb joint dynamics. These additional studies would allow for a comprehensive understanding of how footwear affects not only the ankle but also hip and knee joint movements across a wider population. Because this study collected gait data under conditions similar to those of daily life, it provides data suitable for practical applications and may contribute to future research focused on evaluating gait in everyday scenarios.

Line numbers where the changed text appears: 342-364

[Comment #2]
Line 57
you claim "several prior studies" but only cited two. I recommend providing 4-6 total references related to high-heel research but perhaps highlighting only the two
[Author action #2]
Thank you for your valuable feedback. We have added two more references related to high-heel research, bringing the total to four references. As per your suggestion, we have highlighted the two most relevant references.
Additional Previous Research:

12. Alkjaer T, Raffalt P, Petersen NC, Simonsen EB. Movement Behavior of High-Heeled Walking: How Does the Nervous System Control the Ankle Joint during an Unstable Walking Condition? PLoS ONE. 2012;7(5). doi: https://doi.org/10.1371/journal.pone.0037390

13. Falola JM, Koussihouèdé FEN, Falola SMD, Avossevou YG, Bio NI, Dansou HP. Effect of the Base of the Shoe Heel on Postural Stability During Walking in Women. American Journal of BioScience. 2015;3(5):167-170. doi: 10.11648/j.ajbio.20150305.11

[Comment #3]
Lione 101
if you successfully measure three steps in 9 seconds, the average value will be recorded. this phrase is grammatically incorrect, ie, use of the phrase "if you"
[Author action #3]
Thank you for your valuable feedback. We have revised the sentence for clarity and grammatical accuracy. The changed text is highlighted in yellow as follows:
upon detecting the start of a gait cycle, it measures parameters for three steps within the following 9 s.
Line numbers where the changed text appears: 159

[Comment #4]
Line121
Please provide information about the data collection frequency and any calibration procedures related to the IMU.
Was there only one testing session?

[Author action #4]
Thank you for your valuable feedback. We have addressed your comment as follows:

Regarding calibration, the accuracy and validity of the calculated parameters are ensured based on prior studies. Additionally, we have emphasized in the manuscript that these parameters were validated in previous studies for accuracy or were derived from validated parameters [25,26,27], ensuring the reliability of the measurements.
Regarding the test session, the details of the procedure have been described in the "Procedure" section for further clarity.
We appreciate your time and consideration in reviewing our revisions and would be grateful if you could confirm the updated content at your convenience.
The changed text is highlighted in yellow as follows:

Equipment

The IMS System (A-RROWG, NEC Corporation, Tokyo, Japan) was used for the measurements. The IMS is a device used for gait analysis and consists of key components including an ARM Cortex-M4F MCU (nRF52832 by Nordic Semiconductor). This component features a 64 MHz CPU, 64 KB of RAM, and 512 KB of ROM. Additionally, it includes a Bosch IMU (BMI160) for precise motion tracking, an ABLIC EEPROM (S-24C32C, 32K-bit) for storage, and an Epson real-time clock (RX8130CE). This device performs gait measurements at 2-minute intervals, capturing gait parameters on both sides. The system detects the gait cycle during flat walking, operates for 1 min, and then enters sleep mode to conserve energy. During this 1-minute period, it detects vibrations and identifies stable walking patterns, and upon detecting the start of a gait cycle, it measures the parameters for three steps within the following 9 s. These steps do not need to be consecutive; if the three steps are successfully measured within a specified time, the average value is recorded. If fewer than three steps were

measured, the average value was not recorded. Additionally, this measurement process can be repeated up to three times within a 1-minute window; if all three attempts fail, the measurement for that period is considered unsuccessful, and no data is recorded [25,26,27].

The following 16 parameters are calculated: Stride length, Maximum (peak) dorsiflexion angle, Maximum (peak) plantar flexion angle, Foot clearance, Foot height, Circumduction, Toe-in/out angle, Roll angle of heel contact, Roll angle of toe-off, Walking speed, Peak swing angular velocity, Maximum speed during swinging phase, Stance time, Swing time, Pushing time, and Cadence. Table 1 below, disclosed by NEC Corporation, categorizes the definitions of each parameter into spatial, spatiotemporal, and temporal parameters. These parameters were validated in previous studies for accuracy or were derived from validated parameters [25,26,27].

Line numbers where the changed text appears: 149-176

(Additional references)

25. Fukushi K, Huang C, Wang Z, Kajitani H, Nihey F, Nakahara K. On-line algorithms of stride-parameter estimation for in-shoe motion-sensor system. IEEE Sensors Journal. 2022;22:9636–9648. doi:https://doi.org/10.1109/JSEN.2022.3164057

26. Huang C, Fukushi K, Wang Z, Nihey F, Kajitani H, Nakahara K. An algorithm for real time minimum toe clearance estimation from signal of in-shoe motion sensor. Annual International Conference of the IEEE Engineering in Medicine and Biology Society. 2021: 6775–6778. doi:https://doi.org/10.1109/EMBC46164.2021.9629875

27. Huang C, Fukushi K, Wang Z, Nihey F, Kajitani H, Nakahara K. Method for estimating temporal gait parameters concerning bilateral lower limbs of healthy subjects using a single in-shoe motion sensor through a gait event detection approach. Sensors (Basel). 2022;22(1):351. doi:https://doi.org/10.3390/S22010351

[Comment #5]
Line123 Line142 Table 2
weight
[Author action #5]
Thank you for your valuable feedback. We have revised the words “weight” to “mass” for improved accuracy.

[Comment #6]
Line142
should be mass since units were grams
[Author action #6]
Thank you for your valuable feedback. We have revised the words “weight” to “mass” for improved accuracy.

[Comment #7]
Discussion
please briefly also state the problem you identified and the rationale/need for this study

[Author action #7]
Thank you very much for your valuable feedback. As you suggested, I have clarified the theoretical rationale and need for this study in the Discussion, especially in comparison with prior studies. In doing so, we re-evaluated the novelty of this study, which we believe primarily consists of the following three aspects:

1.Measurement under conditions close to daily life: Unlike other studies that use prescribed footwear, our study measured participants in their habitual shoes, collecting data that more accurately represents natural, everyday walking styles. Furthermore, many previous studies have conducted measurements on treadmills, which differ significantly from actual walking conditions. By performing measurements in real-life conditions, our study is able to provide practical insights that more realistically reflect individual walking patterns.

2.High-precision data collection of daily movement using IMS: Instead of traditional motion capture systems or treadmill-based experiments, we utilized the In-Shoe Motion Sensor (IMS) system, allowing measurements in an environment closer to everyday life. This enables a realistic evaluation of the impact of wearing high heels in typical environments outside the laboratory, offering valuable feedback applicable to real-world conditions.

3.Practical application perspective: The data collection method using IMS serves as foundational data that can be leveraged to evaluate fall risks in everyday walking and provide feedback for designing comfortable and safe footwear. This data can be applied in the future to develop more refined footwear suited for everyday walking and create effective gait training programs, adding further value to this study.

Anyway, the changed text is highlighted in yellow as follows:

(The introduction for Discussion)

This study aimed to evaluate the effect of high-heeled shoes on gait parameters in healthy adult women using an IMS system. In previous studies, participants often wore unfamiliar shoes and measurements were commonly conducted on a treadmill at a constant speed, which may not accurately reflect natural gait patterns in real-life settings. In contrast, this study utilized a compact and user-friendly IMS to collect gait data while participants wore their own familiar shoes. This approach allowed for

accurate collection in a manner closer to daily life conditions, providing foundational data for assessing the effects of footwear under more realistic circumstances.

Line numbers where the changed text appears: 238-246

(Conclusion)

Because this study collected gait data under conditions similar to those of daily life, it provides data suitable for practical applications and may contribute to future research focused on evaluating gait in everyday scenarios.

Line numbers where the changed text appears: 355-364

[Comment #8]
Line 169
can results
[Author action #8]
Thank you for pointing out the grammatical issue. We have revised the sentence to ensure it adheres to proper grammatical standards.

[Comment #9]
Line187 “A higher toe angle indicates a larger ankle eversion angle.”
this is not intuitive and so it needs some explanation along with citing a reference
[Author action #9]
Thank you for your comment. I made a correction regarding the angle described. As mentioned in Table 1, the term “toe angle” in the manuscript should refer to abduction, not eversion. We're sorry for my mistake. We have revised the text to ensure the correct terminology is used.
The changed text is highlighted in yellow as follows:
A higher toe angle indicates a larger ankle abduction angle.
Line numbers where the changed text appears: 307

[Comment #10]
Conclusion

a conclusion needs to be more than a summary of major findings; please expand and consider expressing possible implications of your findings

[Author action #10]

Thank you very much for your valuable feedback. As you pointed out, I have expanded the conclusion to include the potential implications of the results. Additionally, I have revised other sections based on your suggestions to make the content more appropriate. I would appreciate it if you could review it at your convenience.

The changed text is highlighted in yellow as follows:

These findings suggest that wearing high-heeled shoes has a substantial impact on gait stability and efficiency, potentially increasing the risk of falls and musculoskeletal strain.

Line numbers where the changed text appears: 355-357

Reviewer #2:

[Comment #1]

My opinions are as follows:

- There are many studies in the literature on this subject (There is a systematic review and meta-analysis). Please clearly state the difference between your study and other studies. Similar biomechanical parameters to the ones you evaluated have been given in many studies. This study does not yield any different results than those studies, nor does it provide information that would help form a different hypothesis. I do not think that the study will add much to the literature.

Zeng et al. 2023. Effects of high-heeled shoes on lower extremity biomechanics and balance in females: a systematic review and meta-analysis.

[Author action #1]

Thank you very much for your valuable feedback. I apologize for the similarity to my response to Reviewer 1, but as you correctly pointed out, I have revised the Discussion section to clarify the novelty of this study in comparison with previous research. Additionally, I aimed to make this study as meaningful as possible for future research by providing a more detailed description of each parameter.

In revising, I re-evaluated the unique aspects of this study and identified three main points that highlight its novelty. The Discussion was revised based on these three points:

1.**Measurement Under Real-Life Conditions:** Unlike other studies that often employ standardized footwear, this study measured participants in the shoes they habitually wear, thus capturing data that closely reflects real-life walking patterns. Many previous

studies have conducted evaluations using treadmill walking, which does not fully represent natural gait. By conducting measurements under conditions equivalent to daily life, this study provides a more realistic reflection of individual walking patterns and contributes valuable practical insights.

2.**High-Precision Data Collection of Everyday Movements Using IMS:** Replacing conventional motion capture systems and treadmill-based experiments, this study utilized an In-Shoe Motion Sensor (IMS) system, allowing for assessments in settings closer to everyday environments. This approach enables a more realistic evaluation of the effects of high-heeled shoes outside of laboratory conditions, providing insights that are applicable to real-life contexts.

3.**Practical Applicability:** The IMS-based data collection method offers foundational data that can be used to assess fall risks during daily walking and to inform the design of safer and more comfortable footwear. This data adds value by potentially contributing to the development of refined shoe designs suited for daily walking and to more effective gait training methods in the future.

Additionally, the changed text is highlighted in yellow as follows:

(The Introduction)

Research on the effects of high-heeled shoes on gait parameters has significantly progressed. However, many evaluations have been conducted using a treadmill with participants walking at a constant speed [14]. Treadmill walking occurs under flat, steady-speed conditions, which differs from everyday walking environments [15]. For example, because a treadmill maintains a fixed speed, participants cannot walk at their natural pace, potentially suppressing their typical gait patterns [16,17]. Additionally, on a treadmill, individuals often need to adjust their posture and gait mechanics to match the treadmill's motion in order to maintain visual and balance cues, which is known to influence gait biomechanics [15]. In certain studies, participants wore unfamiliar shoes that may not accurately reflect their usual gait patterns in daily life [11,14]. Shoes that

are unfamiliar or uncomfortable for an individual often do not fit well with the person's foot shape or gait style, potentially affecting the gait biomechanics [18]. Specifically, when the shoe's size or shape does not align with the foot, it may cause foot pain and fatigue, while also reducing stability and increasing the risk of falls during walking [19]. Therefore, conditions different from those in daily life may alter an individual's natural gait patterns and may not accurately reflect habitual walking [11].

As a more suitable evaluation method, Inertial Measurement Units (IMUs) can be used. IMUs can estimate three-dimensional movement from angular velocity and acceleration [20,21]. Due to their low cost, small size, and ease of use, they have been widely used to evaluate daily life walking in recent years [22]. By using the In-Shoe Motion Sensor system (IMS), which measures the acceleration and angular velocity of the feet using IMUs and calculates walking parameters, it is possible to obtain walking data comparable to daily life more accurately than conventional studies-[23].

Line numbers where the changed text appears: 86-119

(The introduction for Discussion)

This study aimed to evaluate the effect of high-heeled shoes on gait parameters in healthy adult women using an IMS system. In previous studies, participants often wore unfamiliar shoes and measurements were commonly conducted on a treadmill at a constant speed, which may not accurately reflect natural gait patterns in real-life

settings. In contrast, this study utilized a compact and user-friendly IMS to collect gait data while participants wore their own familiar shoes. This approach allowed for accurate collection in a manner closer to daily life conditions, providing foundational data for assessing the effects of footwear under more realistic circumstances.

Line numbers where the changed text appears: 238-246

(Conclusion)

Because this study collected gait data under conditions similar to those of daily life, it provides data suitable for practical applications and may contribute to future research focused on evaluating gait in everyday scenarios.

Line numbers where the changed text appears: 355-364

[Comment #2]
- There is no sample size estimation in your study. One would always like to conduct a study that has adequate sample size and power so that the conclusions generated from the experiment can be applied to the broader population with ample confidence. The required sample size to test a hypothesis is governed by the effect size. Sample size was not calculated in the study. The power of the study should be calculated and added. Because, our aim should be to catch the most significant and best difference by performing the strongest statistical tests with the optimal sample size. Sample size calculation is of great importance here.

[Author action #2]
Thank you for your valuable comment. The sample size for this study was estimated based on the findings of Chard et al (2013), a prior study closely related to our research. Specifically, we calculated a sample size of 15 participants needed to detect significant differences, using an effect size of 0.62, an α level of 0.05, and a statistical power of 0.8. Additionally, we accounted for a potential dropout rate by recruiting several more 5

participants to ensure adequate power for data analysis. Details of this estimation are included in the Participants section.

The changed text is highlighted in yellow as follows:

The sample size for this study was calculated using G Power 3.1 (Heinrich Heine University). Based on prior research [24], with an assumed effect size of 0.62, an alpha level of 0.05, and a statistical power of 0.8, the minimum required number of participants was calculated to be 15. To account for potential data loss or incomplete data, an additional five participants were included, setting the final target number of participants at 20.

Line numbers where the changed text appears:132-136

(Additional references)

24. Chard A, Greene A, Hunt A, Vanwanseele B, Smith R. Effect of thong style flip-flops on children's barefoot walking and jogging kinematics. Journal of Foot and Ankle Research. 2013;6:8. doi:https://doi.org/10.1186/1757-1146-6-8

[Comment #3]

Since no power analysis was performed and it is difficult to interpret the obtained findings, thus, the discussion.

[Author action #3]

Thank you for your valuable comment. The sample size for this study was estimated based on the findings of Chard et al (2013), a prior study closely related to our research. Specifically, we calculated a sample size of 15 participants needed to detect significant differences, using an effect size of 0.62, an α level of 0.05, and a statistical power of 0.8. Additionally, we accounted for a potential dropout rate by recruiting several more 5 participants to ensure adequate power for data analysis. Based on this estimation, we

believe the sample size in our study is appropriate. Thank you for your understanding. Details of this estimation are included in the Participants section.
The changed text is highlighted in yellow as follows:

The sample size for this study was calculated using G Power 3.1 (Heinrich Heine University). Based on prior research [24], with an assumed effect size of 0.62, an alpha level of 0.05, and a statistical power of 0.8, the minimum required number of participants was calculated to be 15. To account for potential data loss or incomplete data, an additional five participants were included, setting the final target number of participants at 20.

Line numbers where the changed text appears: 132-136

(Additional references)

24. Chard A, Greene A, Hunt A, Vanwanseele B, Smith R. Effect of thong style flip-flops on children's barefoot walking and jogging kinematics. Journal of Foot and Ankle Research. 2013;6:8. doi:https://doi.org/10.1186/1757-1146-6-8

Reviewer #3:
[Comment #1]
Introduction:
Clarity of Rationale: The introduction touches upon various studies but lacks a clear argument for why this study is unique. It's essential to emphasize the gaps in previous research more clearly. For instance, highlight that previous studies used treadmill walking or unfamiliar footwear, and explain why in-shoe motion sensors offer a more reliable approach.
[Author action #1]
Thank you for your feedback. As you pointed out, we had not clearly articulated the gap between this study and previous research. Based on your suggestions, we have added a

rationale explaining the issues with using treadmill walking and unfamiliar footwear, as well as why motion sensors provide a more reliable approach.

The changed text is highlighted in yellow as follows:

Research on the effects of high-heeled shoes on gait parameters has significantly progressed. However, many evaluations have been conducted using a treadmill with participants walking at a constant speed [14]. Treadmill walking occurs under flat, steady-speed conditions, which differs from everyday walking environments [15]. For example, because a treadmill maintains a fixed speed, participants cannot walk at their natural pace, potentially suppressing their typical gait patterns [16,17]. Additionally, on a treadmill, individuals often need to adjust their posture and gait mechanics to match the treadmill's motion in order to maintain visual and balance cues, which is known to influence gait biomechanics [15]. In certain studies, participants wore unfamiliar shoes that may not accurately reflect their usual gait patterns in daily life [11,14]. Shoes that are unfamiliar or uncomfortable for an individual often do not fit well with the person's foot shape or gait style, potentially affecting the gait biomechanics [18]. Specifically, when the shoe's size or shape does not align with the foot, it may cause foot pain and fatigue, while also reducing stability and increasing the risk of falls during walking [19]. Therefore, conditions different from those in daily life may alter an individual's natural gait patterns and may not accurately reflect habitual walking [11].

As a more suitable evaluation method, Inertial Measurement Units (IMUs) can be used. IMUs can estimate three-dimensional movement from angular velocity and acceleration [20,21]. Due to their low cost, small size, and ease of use, they have been widely used to evaluate daily life walking in recent years [22]. By using the In-Shoe Motion Sensor system (IMS), which measures the acceleration and angular velocity of the feet using IMUs and calculates walking parameters, it is possible to obtain walking data comparable to daily life more accurately than conventional studies-[23].

Line numbers where the changed text appears: 86-119

[Comment #2]
Study Objectives: The objective of the study is mentioned indirectly, but it should be explicitly stated at the end of the introduction. Clearly articulate the novelty and specific aims of your study beyond what has been done before.
[Author action #2]
Thank you for your insightful feedback. In response to your comments, we have clarified the objectives of our study, highlighting how it differs from previous research. The changed text is highlighted in yellow as follows:

Therefore, this study aimed to evaluate the impact of high-heeled shoes on gait parameters in healthy adult women using the IMS system. Unlike previous studies that typically used treadmill walking at a constant speed or required participants to wear unfamiliar shoes, This study allowed participants to walk in their own footwear that fitted their feet and that they were accustomed to, under conditions similar to those of daily life. This approach enabled us to capture a more natural gait pattern, closely

resembling everyday walking, and to more accurately reflect the effects of high-heeled shoes on gait parameters.

Line numbers where the changed text appears: 113-119

[Comment #3]
Methods:
Participant Recruitment and Demographics: The participant demographics (age, height, weight) are provided, but you need to offer more details on how participants were selected. For example, how did the recruitment process ensure variability in walking experience with high heels? Were any baseline tests performed to evaluate walking proficiency in high heels?
[Author action #3]
Thank you for your feedback. In this study, we primarily targeted women who commonly have opportunities to wear high-heeled shoes in daily life, based on previous research (Annoni et al., 2023). In the revised manuscript, we have described the participant selection process in as much detail as possible based on this context. I would appreciate it if you could review it at your convenience.
The changed text is highlighted in yellow as follows:
The participants in this study were healthy adult women enrolled at Kyoto University. The inclusion criteria were individuals aged 18 years and older who could walk for at least 6 minutes without rest. To minimize the influence of unfamiliarity with high-heeled shoes on the results, we selected participants with extensive experience wearing high heels in various settings, such as work, social events, shopping, or formal occasions, based on previous studies [14]. The exclusion criteria were individuals with neurological or orthopedic conditions that could affect walking function. The purpose of the study was thoroughly explained to the participants, and their consent was obtained prior to measurement.
Line numbers where the changed text appears: 127-129

(Additional references)

14. Annoni I, Mapelli A, Sidequersky FV, Zago M, Sforza C. The effect of high-heeled shoes on overground gait kinematics in young healthy women. Sport Sciences for Health. 2014;10(2):149-157. doi: 10.1007/s11332-014-0191-z

[Comment #4]
Sample Size: The study includes only 17 participants. Was a power analysis conducted to determine whether this sample size is sufficient for detecting significant differences? This omission weakens the study's generalizability and statistical power. Justify the sample size or conduct a power analysis to support your choices.
Shoe Definitions and Comparisons: The criteria for shoe selection are unclear and could be inconsistent. The manuscript states that high-heeled shoes are defined by their heel height but also includes other characteristics like a "narrow toe box." This could lead to confusion about what exactly constitutes a "high-heeled" shoe. A more specific definition is needed.
[Author action #4]
Thank you for your valuable comment. The sample size for this study was estimated based on the findings of Chard et al (2013), a prior study closely related to our research. Specifically, we calculated a sample size of 15 participants needed to detect significant differences, using an effect size of 0.62, an α level of 0.05, and a statistical power of 0.8. Additionally, we accounted for a potential dropout rate by recruiting several more 5 participants to ensure adequate power for data analysis. Based on this estimation, we believe the sample size in our study is appropriate. Thank you for your understanding. Details of this estimation are included in the Participants section.
The changed text is highlighted in yellow as follows:

The sample size for this study was calculated using G Power 3.1 (Heinrich Heine University). Based on prior research [24], with an assumed effect size of 0.62, an alpha level of 0.05, and a statistical power of 0.8, the minimum required number of participants was calculated to be 15. To account for potential data loss or incomplete

data, an additional five participants were included, setting the final target number of participants at 20.

Line numbers where the changed text appears: 132-136

(Additional references)

24. Chard A, Greene A, Hunt A, Vanwanseele B, Smith R. Effect of thong style flip-flops on children's barefoot walking and jogging kinematics. Journal of Foot and Ankle Research. 2013;6:8. doi:https://doi.org/10.1186/1757-1146-6-8

Regarding definitions, in this study, high-heeled shoes were defined as "footwear with a heel elevated higher than the forefoot, a narrow toe box, and a curved sole." This definition is based on existing literature that has demonstrated the specific effects of high heels on walking parameters. In contrast, sneakers were defined as "light footwear that has a top made of cloth and a bottom made of rubber." To the best of our knowledge, no academic definition of "sneakers" was available; thus, we referred to the general definition from the Cambridge Dictionary. Sneakers were chosen as the control group because obtaining walking data with footwear familiar to participants allows for a walking pattern more reflective of daily life.
These definitions were included in the "Equipment" section as follows:

High-heeled shoes were defined as "footwear with a heel elevated higher than the forefoot, a narrow toe box, and a curved sole" [11], while sneakers were defined as "light footwear that has a top made of cloth and a bottom made of rubber."

Line numbers where the changed text appears: 173-176

[Comment #5]
Data Collection Process: The measurement process with IMS should be explained with greater detail. For example, how were the intervals between measurements selected? Why are only three steps used for calculating parameters? Provide a rationale for using

these specific methods, or cite prior studies that validate this approach.
[Author action #5]
Thank you for your valuable feedback. The above methods are established by the manufacturer according to prior research, and their accuracy is validated through rigorous validation processes. I have added relevant prior studies and provided a more detailed description. I would appreciate it if you could review it at your convenience. The changed text is highlighted in yellow as follows:

Equipment

The IMS System (A-RROWG, NEC Corporation, Tokyo, Japan) was used for the measurements. The IMS is a device used for gait analysis and consists of key components including an ARM Cortex-M4F MCU (nRF52832 by Nordic Semiconductor). This component features a 64 MHz CPU, 64 KB of RAM, and 512 KB of ROM. Additionally, it includes a Bosch IMU (BMI160) for precise motion tracking, an ABLIC EEPROM (S-24C32C, 32K-bit) for storage, and an Epson real-time clock (RX8130CE). This device performs gait measurements at 2-minute intervals, capturing gait parameters on both sides. The system detects the gait cycle during flat walking, operates for 1 min, and then enters sleep mode to conserve energy. During this 1-minute period, it detects vibrations and identifies stable walking patterns, and upon detecting the start of a gait cycle, it measures the parameters for three steps within the following 9 s. These steps do not need to be consecutive; if the three steps are successfully measured within a specified time, the average value is recorded. If fewer than three steps were measured, the average value was not recorded. Additionally, this measurement process

can be repeated up to three times within a 1-minute window; if all three attempts fail, the measurement for that period is considered unsuccessful, and no data is recorded [25,26,27].

The following 16 parameters are calculated: Stride length, Maximum (peak) dorsiflexion angle, Maximum (peak) plantar flexion angle, Foot clearance, Foot height, Circumduction, Toe-in/out angle, Roll angle of heel contact, Roll angle of toe-off, Walking speed, Peak swing angular velocity, Maximum speed during swinging phase, Stance time, Swing time, Pushing time, and Cadence. Table 1 below, disclosed by NEC Corporation, categorizes the definitions of each parameter into spatial, spatiotemporal, and temporal parameters. These parameters were validated in previous studies for accuracy or were derived from validated parameters [25,26,27].

Line numbers where the changed text appears: 143-171

[Comment #6]
Results:
Lack of Effect Sizes: The results section presents P-values but lacks effect sizes, which are crucial for understanding the magnitude of differences between high heels and sneakers. Please include effect sizes for all statistically significant results.
[Author action #6]
Thank you for your valuable feedback regarding the need to include effect sizes in the results section. As suggested, we have now included effect sizes for all statistically significant results. Specifically, we have calculated both Cohen's d and Hedges' g to provide a clearer understanding of the magnitude of the differences between the conditions (high heels vs. sneakers).
The changed table is highlighted in yellow as follows:

Classification	Parameters	HG	SG	P-value	Effect Size (Cohen's d)
**Spatial parameters**	Stride length (cm)	130.42±18.14	143.88±16.09	0.0006*	-1.09
	Maximum (peak) dorsiflexion angle (degree)	24.21±5.25	29.88±5.13	0.0017*	-0.94
	Maximum (peak) plantar flexion angle (degree)	62.33±11.15	78.31±6.49	<0.0001*	-1.47
	Foot clearance (cm)	21.17±3.31	24.05±2.15	0.0005*	-1.09
	Foot height (cm)	13.33±2.40	15.70±1.76	0.0023*	-0.90
	Circumduction (cm)	4.10±1.58	3.72±2.01	0.5397	0.15
	Toe-in/out angle (degree)	12.05±8.82	10.14±7.97	0.0476*	0.54
	Roll angle of heel contact (degree)	5.21±4.86	3.71±5.70	0.2343	0.30

	Roll angle of toe-off (degree)	-2.05±6.17	-1.36±4.52	0.5178	-0.17
**Spatiotemporal parameters**	Walking speed (km/h)	4.61±0.68	5.08±0.66	0.0005*	-1.09
	Peak swing angular velocity (degree/s)	531.64±95.96	565.64±75.54	0.0932	-0.45
	Maximum speed during swinging phase (km/h)	15.15±1.65	15.97±1.59	0.0085*	-0.75
**Temporal parameters**	Stance time(s)	0.62±0.05	0.62±0.04	0.8271	-0.06
	Swing time(s)	0.41±0.04	0.40±0.01	0.3225	0.03
	Pushing time (s)	0.19±0.03	0.20±0.02	0.6805	−0.13
	Cadence (steps/min)	117.22±6.41	116.92±5.69	0.8030	0.06

SD, standard deviation. *p<0.05.

Effect size (Cohen's d): Small = 0.2–0.49, Medium = 0.5–0.79, Large ≥ 0.8

Line numbers where the changed text appears: 232

[Comment #7]
Detailed Parameter Reporting: Some parameters such as circumduction and roll angle show no significant difference but are still discussed in detail. Revisit how you report and discuss non-significant findings. Focus should be on significant outcomes unless there is a theoretical justification for the non-significant results.
[Author action #7]
Thank you for your valuable feedback. We acknowledge that providing theoretical justification for non-significant findings is challenging. To maintain the quality of our study, we have removed discussions on non-significant parameters and have elaborated more thoroughly on the significant results.

[Comment #8]
Discussion:
In-depth Analysis: The discussion needs more depth, especially concerning the mechanisms underlying the changes in gait parameters. For example, why do high heels specifically reduce stride length and foot clearance? Refer to the biomechanical literature more thoroughly.
[Author action #8]
Thank you very much for your valuable feedback. As per your suggestion, I have provided a more detailed discussion. I would appreciate it if you could review it at your convenience. The changed table is highlighted in yellow as follows:

Regarding spatial parameters such as walking speed, stride length, and maximum swing speed, it is generally known that wearing high-heeled shoes reduces the base of support during walking, prompting individuals to adopt a safer and more stable gait pattern [28,29]. Additionally, to minimize increases in energy expenditure, they tend to decrease their walking speed and stride length as a result [30,31]. In addition to these stable and efficient walking strategies, previous studies have shown that wearing high heels brings the ground reaction force closer to the ankle joint, and the curved shape of the shoe sole causes the ankle to remain in a plantarflexed position [11]. These factors

can shorten the length of the triceps surae muscle and Achilles tendons. Consequently, compared to sneakers or barefoot walking, ankle plantarflexion moments decrease, and the effect of the stretch-shortening cycle on the muscle-tendon complex is reduced [32,33]. The stretch-shortening cycle is a process in which muscles and tendons quickly stretch and then shorten, store, and release elastic energy, and plays a crucial role in efficient force production [33]. However, the shortened state of the muscle-tendon complex induced by wearing high heels is thought to impede this function, leading to decreased energy efficiency and a weaker push-off force. As a result, the calculated parameters were significantly different between the two groups.

Similarly, foot clearance and foot height were significantly lower in the high-heeled group. As noted above, wearing high-heeled shoes is suggested to reduce the push-off force of the ankle. Additionally, previous studies investigating the role of the ankle in walking have shown that ankle push-off contributes to up to 50% of the power needed for leg swinging during the terminal stance and early swing phases [34,35,36]. This reduction in ankle push-off may decrease the leg swing speed, which is essential for the double-pendulum effect during walking [37]. Consequently, a lack of sufficient knee flexion in the double-pendulum mechanism may cause the lower leg to remain relatively low, potentially resulting in reduced foot clearance. In fact, prior research comparing

joint angles in the sagittal plane under different heel heights at a constant walking speed found that while hip flexion during the swing phase remained unaffected by heel height, the knee flexion angle significantly decreased as heel height increased [38,39,40]. Moreover, a decrease in the foot clearance height can increase the risk of tripping over small obstacles or uneven surfaces while walking, potentially leading to falls [41]. High-heeled shoes also tilt the toes downward and elevate the heel, reducing the contact area of the foot with the ground, which may further destabilize balance. This combination of reduced foot clearance and a limited base of support likely exacerbates the risk of falls [14].

Line numbers where the changed text appears: 266-303

[Comment #9]
Missing Literature Context: While the discussion refers to previous studies, it does not critically evaluate their methods or findings in relation to the current study. More effort should be made to situate your results within the broader field of biomechanics and footwear research.

[Author action #9]
Thank you very much for your valuable feedback. As you suggested, I have clarified the theoretical rationale and need for this study in the Discussion, especially in comparison with prior studies. In doing so, we re-evaluated the novelty of this study, which we believe primarily consists of the following three aspects:

1.Measurement under conditions close to daily life: Unlike other studies that use prescribed footwear, our study measured participants in their habitual shoes, collecting data that more accurately represents natural, everyday walking styles. Furthermore, many previous studies have conducted measurements on treadmills, which differ significantly from actual walking conditions. By performing measurements in real-life

conditions, our study is able to provide practical insights that more realistically reflect individual walking patterns.

2.High-precision data collection of daily movement using IMS: Instead of traditional motion capture systems or treadmill-based experiments, we utilized the In-Shoe Motion Sensor (IMS) system, allowing measurements in an environment closer to everyday life. This enables a realistic evaluation of the impact of wearing high heels in typical environments outside the laboratory, offering valuable feedback applicable to real-world conditions.

3.Practical application perspective: The data collection method using IMS serves as foundational data that can be leveraged to evaluate fall risks in everyday walking and provide feedback for designing comfortable and safe footwear. This data can be applied in the future to develop more refined footwear suited for everyday walking and create effective gait training programs, adding further value to this study.

Anyway, the changed text is highlighted in yellow as follows:

(The introduction for Discussion)

This study aimed to evaluate the effect of high-heeled shoes on gait parameters in healthy adult women using an IMS system. In previous studies, participants often wore unfamiliar shoes and measurements were commonly conducted on a treadmill at a constant speed, which may not accurately reflect natural gait patterns in real-life settings. In contrast, this study utilized a compact and user-friendly IMS to collect gait data while participants wore their own familiar shoes. This approach allowed for accurate collection in a manner closer to daily life conditions, providing foundational data for assessing the effects of footwear under more realistic circumstances.

Line numbers where the changed text appears: 239-246

(Conclusion)

Because this study collected gait data under conditions similar to those of daily life, it provides data suitable for practical applications and may contribute to future research focused on evaluating gait in everyday scenarios.

Line numbers where the changed text appears: 361-364

[Comment #10]
Limitations: Two limitations are briefly mentioned, but these should be expanded. For example, the small sample size and homogeneity of the participants (all from one university) limit generalizability. Additionally, the lack of measures on hip and knee movements should be discussed more critically as it limits the study's ability to fully assess gait mechanics.

[Author action #10]
Thank you for bringing this point to our attention. As per your suggestion, we have revised the Limitations section. The changed text is highlighted in yellow as follows:

There are limitations in this study; the participants were limited to healthy adult women from the same university, which restricts the generalizability of our findings. To apply these results to other age groups, sexes, or individuals with specific health conditions, further studies with more diverse participants are necessary. Second, we were unable to capture the movements of the hip and knee joints, making it challenging to fully assess how these joints affect gait mechanics, particularly balance and posture control. Specifically, it remains unclear to what extent high-heeled shoes stress on these joints under near-daily conditions and how this stress might increase the risk of falls or lead to long-term joint disorders. Future research should incorporate devices that

measure the movements of these joints to provide a more comprehensive evaluation of the impact of different footwear types on gait and balance.

Line numbers where the changed text appears: 331-340

[Comment #11]
Future Directions: The recommendation for future studies is too brief and needs elaboration. What specific devices or methods would you propose for capturing knee and hip joint movements? How should the participant pool be expanded?
[Author action #11]
Thank you for your insightful comment. Based on your suggestion, We have revised the future studies section in Conclusion. The changed text is highlighted in yellow as follows:
Future studies should include a more diverse population, such as older adults or individuals with joint disorders, and incorporate motion capture systems or wearable sensors that can measure lower limb joint dynamics. These additional studies would allow for a comprehensive understanding of how footwear affects not only the ankle but also hip and knee joint movements across a wider population.
Line numbers where the changed text appears: 357-361

[Comment #12]
Statistical Analysis:
Detailed Explanation of Tests Used: The manuscript mentions using Shapiro-Wilk tests but does not explain the justification for using paired t-tests or Wilcoxon signed-rank tests. Were these tests chosen based on normality results? Clarify the decision-making process behind the statistical methods.
[Author action #12]
Thank you for your feedback. We have followed your instructions and made the necessary revisions as per your guidance. The changed text is highlighted in yellow as follows:

Data analysis

Statistical analyses were performed using the JMP PRO16 software (SAS Institute).

First, the Shapiro-Wilk test was conducted to determine whether each dataset followed a

normal distribution. For data that were normally distributed, a paired t-test was used, because the same participants were measured under different conditions (high-heeled shoes and sneakers). For data that did not follow a normal distribution, the Wilcoxon signed-rank test, suitable for paired datasets, was applied. The group wearing high-heeled shoes was referred to as the “high-heeled group” (HG) and the group wearing sneakers as the “sneaker group” (SG). Data are presented as mean standard deviation ± standard deviation(SD). The effect size was evaluated using Cohen’s d to assess the magnitude of the differences between groups. The statistical significance level for this study was set at less than 5%.

Line numbers where the changed text appears: 200-213

[Comment #13]
Conclusion:
Overstatement of Findings: The conclusion overstates the implications of the study, particularly when claiming the study “provides new insights into injury prevention.” While the results are interesting, the small sample size and limitations in the methodology weaken such a broad conclusion. Focus on the specific parameters measured rather than general injury prevention claims.
[Author action #13]
Thank you very much for your valuable feedback. Based on your comments and other suggestions, I have revised the conclusion. I focused on describing the specific parameters measured. Additionally, after estimating the sample size based on previous studies, we believe that the number of participants in our study is reasonable. The methodology has also been described in greater detail in response to your feedback. Furthermore, we have adjusted the general claims to be more appropriate, taking prior research into consideration. I would appreciate it if you could review it at your convenience.

The changed text is highlighted in yellow as follows:

Conclusions

This study investigated the effects of high-heeled shoes on walking parameters in healthy adult women using the IMS. As a result, in terms of spatial parameters, the high-heeled group showed significantly shorter stride length, lower maximum dorsiflexion angle, lower maximum plantar flexion angle, and significantly higher toe-out angle compared to the sneaker group, with effect sizes ranging from moderate to high. Additionally, in spatiotemporal parameters, the high-heeled group exhibited significantly lower walking speed and maximum speed during the swinging phase, with large effect sizes. However, no significant differences were observed between the groups in temporal parameters.

These findings suggest that wearing high-heeled shoes has a substantial impact on gait stability and efficiency, potentially increasing the risk of falls and musculoskeletal strain. Future studies should include a more diverse population, such as older adults or individuals with joint disorders, and incorporate motion capture systems or wearable sensors that can measure lower limb joint dynamics. These additional studies would allow for a comprehensive understanding of how footwear affects not only the ankle but also hip and knee joint movements across a wider population. Because this study collected gait data under conditions similar to those of daily life, it provides data

suitable for practical applications and may contribute to future research focused on evaluating gait in everyday scenarios.

Line numbers where the changed text appears: 355-364

[Comment #14]
Data Availability: Ensure that all data used in the study is easily accessible for replication. The statement “available upon request” may not meet journal guidelines. Consider uploading datasets to a repository like Open Science Framework (OSF).
[Author action #14]
Thank you for bringing this point to our attention. We have uploaded all our datasets to a repository in this study for replication.
