# Supplementary material for: The effects of high-heeled shoes on gait parameters in healthy adult women
Source: PLoS One. 2025 Jul 3;20(7):e0327250. doi: 10.1371/journal.pone.0327250 (PMC12225787; doi:10.1371/journal.pone.0327250)
Supplement: Attachment — Submitted filename: PONE-D-24-32099_R2_reviewer_reviewer comments.pdf [file pone.0327250.s005.pdf]

PLOS ONE

The effects of high-heeled shoes on gait parameters in healthy adult women

--Manuscript Draft--

**Manuscript Number:**	PONE-D-24-32099R2
**Article Type:**	Research Article
**Full Title:**	The effects of high-heeled shoes on gait parameters in healthy adult women
**Short Title:**	The effects of high-heeled shoes on gait parameters
**Corresponding Author:**	Tomoki Aoyama<br>Kyoto University: Kyoto Daigaku<br>JAPAN
**Keywords:**	High-heeled shoes; shoes; walking; women; IMS
**Abstract:**	Walking is an essential aspect of daily life. Research has shown that footwear type significantly affects walking patterns; for example, high-heeled shoes (most commonly worn by women) increase the risk of knee osteoarthritis and falls. However, most studies assessing this influence have used treadmill walking or unfamiliar footwear, which may not reflect daily walking patterns. This study therefore investigated the impact of high-heeled shoes on walking parameters in healthy adult women using in-shoe motion sensors. Seventeen healthy adult women without pain during walking participated in this study. All participants walked for 6 minutes along a 30-meter corridor wearing high-heeled shoes and sneakers. Walking data were recorded using an in-shoe motion sensor system every 2 minutes. The average of three valid consecutive steps was calculated automatically. Statistical analysis was performed to compare the mean walking parameters between the high-heel and sneaker groups. The high-heel group showed significantly lower foot clearance, stride length, peak plantar angle in dorsiflexion, and peak plantar angle in plantarflexion, as well as a significantly higher toe-out angle in spatial parameters. Spatiotemporal parameters revealed significantly lower walking speed and maximum swing phase speed in the high-heel group. No significant inter-group differences were observed in temporal parameters. This study provides insights into possible methods for injury prevention in healthy adult women walking in high-heeled shoes, and thus has the potential to contribute to future research on daily walking evaluations. Future studies should include diverse participants and measurement devices that assess hip and knee joint movements and neurological responses to comprehensively evaluate the impact of high-heeled shoes. Additionally, understanding the biomechanical and neurological effects of different footwear can guide the development of safer shoe designs for daily use.
**Order of Authors:**	Kyoma Tanigawa
	Hiroki Shimizu
	Anuradhi Bandara
	Misa Toyota
	Shota Suzuki
	Momoko Nagai-Tanima
	Tomoki Aoyama
**Response to Reviewers:**	Point-by-point response<br><br>We are very grateful to the editors for providing such valuable, thoughtful, and thorough comments. The comments and questions raised have prompted us to consider our data from new and interesting angles, the culmination of which we feel has greatly strengthened the work. Thank you for your time.<br><br>Editor’s comments:<br>[Comment #1]<br>Limited Contribution to the Literature

Powered by Editorial Manager® and ProduXion Manager® from Aries Systems Corporation

Your study’s findings largely align with existing research. Please clearly articulate what new insights your study provides.
[Author action #1]
Thank you for your thoughtful feedback. We believe we have already addressed our study's novelty and unique insights. We would be grateful if you could review that part, and we’re happy to further clarify or expand on any points if needed. Also, we re-evaluated the novelty of this study, which we believe primarily consists of the following three aspects:

1.Measurement under conditions close to daily life: Unlike other studies that use prescribed footwear, our study measured participants in their habitual shoes, collecting data that more accurately represents natural, everyday walking styles. Furthermore, many previous studies have conducted measurements on treadmills, which differ significantly from actual walking conditions. By performing measurements in real-life conditions, our study is able to provide practical insights that more realistically reflect individual walking patterns.
2.High-precision data collection of daily movement using IMS: Instead of traditional motion capture systems or treadmill-based experiments, we utilized the In-Shoe Motion Sensor (IMS) system, allowing measurements in an environment closer to everyday life. This enables a realistic evaluation of the impact of wearing high heels in typical environments outside the laboratory, offering valuable feedback applicable to real-world conditions.
3.Practical application perspective: The data collection method using IMS serves as foundational data that can be leveraged to evaluate fall risks in everyday walking and provide feedback for designing comfortable and safe footwear. This data can be applied in the future to develop more refined footwear suited for everyday walking and create effective gait training programs, adding further value to this study.
The specific parts demonstrating the novelty are as follows:

(The Introduction)
Research on the effects of high-heeled shoes on gait parameters has significantly progressed. However, many evaluations have been conducted using a treadmill with participants walking at a constant speed [14]. Treadmill walking occurs under flat, steady-speed conditions, which differs from everyday walking environments [15]. For example, because a treadmill maintains a fixed speed, participants cannot walk at their natural pace, potentially suppressing their typical gait patterns [16,17]. Additionally, on a treadmill, individuals often need to adjust their posture and gait mechanics to match the treadmill’s motion in order to maintain visual and balance cues, which is known to influence gait biomechanics [15]. In certain studies, participants wore unfamiliar shoes that may not accurately reflect their usual gait patterns in daily life [11,14]. Shoes that are unfamiliar or uncomfortable for an individual often do not fit well with the person’s foot shape or gait style, potentially affecting the gait biomechanics [18]. Specifically, when the shoe’s size or shape does not align with the foot, it may cause foot pain and fatigue, while also reducing stability and increasing the risk of falls during walking [19]. Therefore, conditions different from those in daily life may alter an individual’s natural gait patterns and may not accurately reflect habitual walking [11].
As a more suitable evaluation method, Inertial Measurement Units (IMUs) can be used. IMUs can estimate three-dimensional movement from angular velocity and acceleration [20,21]. Due to their low cost, small size, and ease of use, they have been widely used to evaluate daily life walking in recent years [22]. By using the In-Shoe Motion Sensor system (IMS), which measures the acceleration and angular velocity of the feet using IMUs and calculates walking parameters, it is possible to obtain walking data comparable to daily life more accurately than conventional studies [23].
Line numbers where the changed text appears: 81-102

(The introduction for Discussion)
This study aimed to evaluate the effect of high-heeled shoes on gait parameters in healthy adult women using an IMS system. In previous studies, participants often wore unfamiliar shoes and measurements were commonly conducted on a treadmill at a constant speed, which may not accurately reflect natural gait patterns in real-life settings. In contrast, this study utilized a compact and user-friendly IMS to collect gait data while participants wore their own familiar shoes. This approach allowed for accurate collection in a manner closer to daily life conditions, providing foundational data for assessing the effects of footwear under more realistic circumstances.

Powered by Editorial Manager® and ProduXion Manager® from Aries Systems Corporation

Line numbers where the changed text appears: 221-227

(Conclusion)
Because this study collected gait data under conditions similar to those of daily life, it provides data suitable for practical applications and may contribute to future research focused on evaluating gait in everyday scenarios.
Line numbers where the changed text appears: 319-321

[Comment #2]
Sample Size and Power Issues
Seventeen participants are insufficient for drawing meaningful conclusions. Even though you state a power calculation, the rationale for choosing an effect size of 0.62 is unclear.
If increasing the sample size is not feasible, discuss this limitation more transparently in the manuscript.
[Author action #2]
Thank you for your valuable comments. We have revised the limitations section to clearly describe the constraints of the sample size and its impact on statistical power. Additionally, we have removed the mention of the sample size from the participants section. We have learned that if increasing the sample size is not feasible in future research, it is important to discuss this limitation more transparently, and we will keep this in mind moving forward. Thank you again for your insights. We appreciate your time in reviewing our revisions at your convenience.
The changed text is highlighted in yellow as follows:
There are limitations in this study; the participants were limited to healthy adult women from the same university, which restricts the generalizability of our findings. To apply these results to other age groups, sexes, or individuals with specific health conditions, further studies with more diverse participants are necessary. Second, the sample size in this study was limited to 17 participants, which poses constraints on statistical robustness and the power to detect effects. In particular, to adequately assess the impact of individual differences, it is essential to include a larger number of participants. Future studies should increase the sample size to enhance statistical robustness and improve the power to detect effects. Finally, we were unable to capture the movements of the hip and knee joints, making it challenging to fully assess how these joints affect gait mechanics, particularly balance and posture control. Specifically, it remains unclear to what extent high-heeled shoes stress on these joints under near-daily conditions and how this stress might increase the risk of falls or lead to long-term joint disorders. Future research should incorporate devices that measure the movements of these joints to provide a more comprehensive evaluation of the impact of different footwear types on gait and balance.
Line numbers where the changed text appears: 293-297

[Comment #3]
Methodology Needs More Justification
Shoe Selection: Your definition of high-heeled shoes is vague. Standardizing heel height and shoe type would improve reproducibility.
Data Collection: The justification for measuring only three steps at a time is weak. Why not use a continuous gait analysis? Provide references that support your approach.
[Author action #3]
Thank you for your insightful comment. The definition of high-heeled shoes used in our study is based on previous research, which we have cited in the manuscript. We would appreciate it if you could review those references for a better understanding of our approach.
Previous research;
Cronin NJ. The effects of high heeled shoes on female gait: A review. Journal of Electromyography and Kinesiology. 2014;24(2):258-263. doi:10.1016/j.jelekin.2014.01.004
About data collection, thank you for your comment. Previous studies have shown that sufficient reliability can be obtained with measurements of three steps, and this approach is particularly effective for evaluating gait parameters. Additionally, to ensure the reliability of the gait data, we perform measurements for three steps, and if the measurement is not successful, we repeat the process of measuring three steps to accurately collect the data.
Previous research;

1.Fukushi K, Huang C, Wang Z, Kajitani H, Nihey F, Nakahara K. On-line algorithms of stride-parameter estimation for in-shoe motion-sensor system. IEEE Sensors Journal. 2022;22:9636–9648. doi:https://doi.org/10.1109/JSEN.2022.3164057
2.Huang C, Fukushi K, Wang Z, Nihey F, Kajitani H, Nakahara K. An algorithm for real time minimum toe clearance estimation from signal of in-shoe motion sensor. Annual International Conference of the IEEE Engineering in Medicine and Biology Society. 2021: 6775–6778. doi:https://doi.org/10.1109/EMBC46164.2021.9629875
3.Huang C, Fukushi K, Wang Z, Nihey F, Kajitani H, Nakahara K. Method for estimating temporal gait parameters concerning bilateral lower limbs of healthy subjects using a single in-shoe motion sensor through a gait event detection approach. Sensors (Basel). 2022;22(1):351. doi:https://doi.org/10.3390/S22010351

[Comment #4]
Weak Biomechanical Explanation
The discussion lacks depth in explaining why high heels alter gait parameters. Consider integrating research on muscle activation, joint mechanics, and kinetic changes caused by high heels.
[Author action #4]
Thank you for your valuable feedback. We have revised the following sections to incorporate a more detailed biomechanical perspective. By adding supplementary explanations to clarify key points, we have aimed to enhance the reader's understanding.
The changed text is highlighted in yellow as follows:
Additionally, when wearing high heels, the reduced base of support during walking leads to increased muscle activity to maintain balance, which in turn tends to increase energy expenditure. To minimize this increase in energy expenditure, it has been shown that walking speed and stride length are reduced as a strategy.
Line numbers where the changed text appears: 239-242
This reduction in ankle push-off may decrease the leg swing speed, which is essential for the double-pendulum effect during walking [37]. In the double-pendulum effect, an increased hip swing speed results in the relative lifting of the lower leg, consequently increasing foot clearance. This occurs as the hip and knee joints work in coordination, with the hip's movement influencing the lower leg's motion. Specifically, a rapid change in the hip angle causes the lower leg to rise, allowing the foot to clear obstacles before making contact with the ground. In the heel group, however, a reduction in leg swing speed leads to insufficient knee flexion in the double-pendulum mechanism, causing the lower leg to remain relatively low and potentially resulting in reduced foot clearance.
Line numbers where the changed text appears: 261-269
[Comment #5]
Please describe the calibration protocol for this device; I assume that is completed before data collection. A couple of concerns with the use of IMUs is 1) data collection interference depending on laboratory conditions, eg, metal, etc., and 2) data drift. Were these issue that you were aware and could evaluate? Please clarify this.
[Author action #5]
Thank you for your insightful comments. As stated in previous studies, this device ensures the accuracy of data collection through real-time correction. By applying this real-time correction, continuous adjustments can be made, allowing for the elimination of experimental environmental errors as much as possible and enabling more precise data measurements. We appreciate your understanding. Additionally, since the experimental environment was designed to closely resemble daily life conditions, it was not possible to achieve complete control. However, as mentioned in prior research, the application of two algorithms—the Three-dimensional Zero-Velocity-Update (3D ZUPT) algorithm and the On-line Stride Segmentation algorithm—ensures measurement accuracy. The on-line stride segmentation algorithm enables stable foot-flat detection using the foot angle, thereby achieving accurate stride segmentation. Meanwhile, the 3D ZUPT algorithm effectively corrects drift errors, allowing for precise stride parameter calculations within the IMS device. By working in conjunction, these algorithms compensate for measurement errors caused by environmental factors and data drift, ensuring highly reliable data collection. Additionally, I have incorporated further explanations into the revised manuscript. I would appreciate it if you could review it at your convenience.
This IMS maintains measurement accuracy by applying real-time correction through two algorithms: the Three-dimensional zero-velocity-update (3D ZUPT) algorithm and

	the On-line stride segmentation algorithm. The on-line stride segmentation algorithm enables stable foot-flat detection using the plantar angle, ensuring precise stride segmentation. Additionally, the 3D ZUPT algorithm effectively compensates for drift errors, allowing for accurate stride parameter computation within the IMS device [25].<br>The changed text is highlighted in yellow as follows: 140-145<br>[Comment #6]<br>please provide a scale with a range of effect size that indicate the strength of the effect size, i.e., its clinial significance.; reference this scale<br>[Author action #6]<br>Thank you for your suggestion. We have revised the manuscript to include a scale that indicates the strength of the effect size and its clinical significance, along with the appropriate reference. Please review the updated section at your convenience.<br>The changed text is highlighted in yellow as follows:<br>The effect size was evaluated using Cohen's d to assess the magnitude of the differences between groups, with thresholds defined as Small (0.2–0.49), Medium (0.5–0.79), and Large (≥ 0.8) [28].<br>Line numbers where the changed text appears: 193-195
Additional Information:	
Question	Response
**Financial Disclosure**<br><br>Enter a financial disclosure statement that describes the sources of funding for the work included in this submission. Review the submission guidelines for detailed requirements. View published research articles from *PLOS ONE* for specific examples.<br><br>This statement is required for submission and **will appear in the published article** if the submission is accepted. Please make sure it is accurate.<br><br>**Funded studies**<br>Enter a statement with the following details:<br>• Initials of the authors who received each award<br>• Grant numbers awarded to each author<br>• The full name of each funder<br>• URL of each funder website<br>• Did the sponsors or funders play any role in the study design, data collection and analysis, decision to publish, or preparation of the manuscript?<br><br>Did you receive funding for this work?	The author(s) received no specific funding for this work.
**Competing Interests**<br><br>Use the instructions below to enter a competing interest statement for this	The authors have declared that no competing interests exist.

submission. On behalf of all authors, disclose any competing interests that could be perceived to bias this work—acknowledging all financial support and any other relevant financial or non-financial competing interests.<br><br>This statement is required for submission and **will appear in the published article** if the submission is accepted. Please make sure it is accurate and that any funding sources listed in your Funding Information later in the submission form are also declared in your Financial Disclosure statement.<br><br>View published research articles from *PLOS ONE* for specific examples.<br><br>**NO authors have competing interests**<br><br>Enter: *The authors have declared that no competing interests exist.*<br><br>**Authors with competing interests**<br><br>Enter competing interest details beginning with this statement:<br><br>*I have read the journal's policy and the authors of this manuscript have the following competing interests: [insert competing interests here]*<br><br>* typeset	
**Ethics Statement**<br><br>Enter an ethics statement for this submission. This statement is required if the study involved:<br><br>• Human participants<br>• Human specimens or tissue<br>• Vertebrate animals or cephalopods<br>• Vertebrate embryos or tissues<br>• Field research<br><br>Write "N/A" if the submission does not	This study was approved by the Kyoto University Medical Ethics Committee (Approval number: R3664-3). Prior to study initiation, written informed consent was obtained from all participants in accordance with the ethical standards and guidelines.

require an ethics statement.<br><br>General guidance is provided below. Consult the submission guidelines for detailed instructions. **Make sure that all information entered here is included in the Methods section of the manuscript.**<br><br>**Format for specific study types**<br><br>**Human Subject Research (involving human participants and/or tissue)**<br>• Give the name of the institutional review board or ethics committee that approved the study<br>• Include the approval number and/or a statement indicating approval of this research<br>• Indicate the form of consent obtained (written/oral) or the reason that consent was not obtained (e.g. the data were analyzed anonymously)<br><br>**Animal Research (involving vertebrate animals, embryos or tissues)**<br>• Provide the name of the Institutional Animal Care and Use Committee (IACUC) or other relevant ethics board that reviewed the study protocol, and indicate whether they approved this research or granted a formal waiver of ethical approval<br>• Include an approval number if one was obtained<br>• If the study involved *non-human primates*, add *additional details* about animal welfare and steps taken to ameliorate suffering<br>• If anesthesia, euthanasia, or any kind of animal sacrifice is part of the study, include briefly which substances and/or methods were applied<br><br>**Field Research**<br><br>Include the following details if this study involves the collection of plant, animal, or other materials from a natural setting:<br>• Field permit number<br>• Name of the institution or relevant body that granted permission	
**Data Availability**	Yes - all data are fully available without restriction

Powered by Editorial Manager® and ProduXion Manager® from Aries Systems Corporation

Authors are required to make all data underlying the findings described fully available, without restriction, and from the time of publication. PLOS allows rare exceptions to address legal and ethical concerns. See the PLOS Data Policy and FAQ for detailed information.<br><br>A Data Availability Statement describing where the data can be found is required at submission. Your answers to this question constitute the Data Availability Statement and **will be published in the article**, if accepted.<br><br>**Important:** Stating 'data available on request from the author' is not sufficient. If your data are only available upon request, select 'No' for the first question and explain your exceptional situation in the text box.<br><br>Do the authors confirm that all data underlying the findings described in their manuscript are fully available without restriction?	
**Describe where the data may be found in full sentences. If you are copying our sample text, replace any instances of XXX with the appropriate details.**<br><br>• If the data are **held or will be held in a public repository**, include URLs, accession numbers or DOIs. If this information will only be available after acceptance, indicate this by ticking the box below. For example: *All XXX files are available from the XXX database (accession number(s) XXX, XXX.*).<br>• If the data are all contained **within the manuscript and/or Supporting Information files**, enter the following: *All relevant data are within the manuscript and its Supporting Information files.*<br>• If neither of these applies but you are able to provide **details of access elsewhere**, with or without limitations, please do so. For example:	"All relevant data are within the paper and its Supporting Information files."

Data cannot be shared publicly because of [XXX]. Data are available from the XXX Institutional Data Access / Ethics Committee (contact via XXX) for researchers who meet the criteria for access to confidential data.<br><br>*The data underlying the results presented in the study are available from (include the name of the third party and contact information or URL).*<br>• This text is appropriate if the data are owned by a third party and authors do not have permission to share the data.<br><br>* typeset	
Additional data availability information:	

Manuscript

Click here to access/download;Manuscript;Manuscript_ver3_Clean

The Effects of High-Heeled Shoes on Gait Parameters in Healthy Adult Women.

Short Title: The effects of high-heeled shoes on gait parameters

Kyoma Tanigawa[a], Hiroki Shimizu[a], Anuradhi Bandara[a], Toyota Misa[a], Shota Suzuki[a], Momoko Nagai-Tanima[a], Tomoki Aoyama*[a]

[a]Department of Physical Therapy, Human Health Sciences, Graduate School of Medicine, Kyoto University, Kyoto, 606-8507, Japan

*Corresponding author: Tomoki Aoyama

E-mail: aoyama.tomoki.4e@kyoto-u.ac.jp

Abstract

Background

Walking is essential in daily life, and footwear type significantly affects walking patterns. High-heeled shoes increase the risk of knee osteoarthritis and falls in women. Traditional studies often use treadmills or unfamiliar footwear, which may not reflect daily walking. This study investigated the impact of high-heeled shoes on walking parameters in healthy adult women using in-shoe motion sensors.

Methods

Seventeen healthy adult women without pain during walking participated. They walked for 6 minutes along a 30-meter corridor wearing high-heeled shoes and sneakers. Walking data were recorded using an in-shoe motion sensor system every 2 minutes. The average of three valid consecutive steps was calculated automatically. Statistical analysis compared the mean walking parameters between the high-heel and sneaker groups.

Results

The high-heel group showed significantly reduced foot clearance, stride length, peak plantar angle in dorsiflexion, and peak plantar angle in plantarflexion, and significantly greater toe-out angle in spatial parameters. Spatiotemporal parameters revealed significantly reduced walking speed and maximum swing

phase speed in the high-heel group. No significant differences were observed in temporal parameters between the groups.

Conclusions

Since this study collected gait data under conditions similar to daily life, it provides data suitable for practical applications and may contribute to future research evaluating everyday gait. Additionally, future studies should include a broader range of participants and incorporate measurement devices capable of capturing hip and knee joint movements, providing a more comprehensive evaluation of the effects of high-heeled shoes on gait in healthy adult women.

Introduction

Walking is a crucial means of transporting the body safely and efficiently [1]. Additionally, it has beneficial effects on both physical and mental health, enhances human quality of life (QOL), and plays a significant role in the prevention and treatment of various diseases [2]. As such, walking is indispensable in daily life. It is also known that walking is influenced by various external factors [3].

One of these external factors is footwear. Shoes come in various types and have multiple functions, including protecting the feet from external environments, assisting walking functions, and adapting to foot deformities. These functions are related to the occurrence of various conditions

such as falls, osteoarthritis, and back pain [4]. Numerous studies have investigated the impact of shoes on walking parameters [5,6]. For example, research comparing walking barefoot and wearing athletic shoes by Shakoor et al. found that wearing athletic shoes decreases step length, increases the number of steps, and reduces joint angles of the lower limbs [5]. Thus, research on how shoes affect walking is of great importance [7].

One type of shoe that warrants attention is high-heeled shoes. High-heeled shoes are characterized by heels higher than the forefoot, narrow toe boxes, and curved soles [8]. High-heeled shoes have been widely worn by women worldwide for centuries, with 37% to 69% of women wearing them regularly and for an average of 1 to 8 hours per day [8]. Despite their regular use, high-heeled shoes are associated with medical conditions, such as osteoarthritis, and can cause frequent falls [9]. The incidence of such injuries doubled from 7.1% to 14.2% between 2002 and 2012 [10]. Therefore, it is crucial to investigate the impact of high-heeled shoes on the walking parameters of healthy adult women [7]. Several previous studies have examined this effect [9,11,12,13]. For instance, it has been shown that wearing high-heeled shoes does not change the temporal walking parameters such as stance time and cadence [11]. Additionally, a study by Barkema et al. noted that the higher the heel height, the greater the maximum knee adduction moment, indicating a relationship between heel height and knee osteoarthritis [9].

Research on the effects of high-heeled shoes on gait parameters has significantly progressed. However, many evaluations have been conducted using a treadmill with participants walking at a constant speed [14]. Treadmill walking occurs under flat, steady-speed conditions, which differs from everyday walking environments [15]. For example, because a treadmill maintains a fixed speed, participants cannot walk at their natural pace, potentially suppressing their typical gait patterns [16,17]. Additionally, on a treadmill, individuals often need to adjust their posture and gait mechanics to match the treadmill's motion in order to maintain visual and balance cues, which is known to influence gait biomechanics [15]. In certain studies, participants wore unfamiliar shoes that may not accurately reflect their usual gait patterns in daily life [11,14]. Shoes that are unfamiliar or uncomfortable for an individual often do not fit well with the person's foot shape or gait style, potentially affecting the gait biomechanics [18]. Specifically, when the shoe's size or shape does not align with the foot, it may cause foot pain and fatigue, while also reducing stability and increasing the risk of falls during walking [19]. Therefore, conditions different from those in daily life may alter an individual's natural gait patterns and may not accurately reflect habitual walking [11].

As a more suitable evaluation method, Inertial Measurement Units (IMUs) can be used. IMUs can estimate three-dimensional movement from angular velocity and acceleration [20,21]. Due to their low cost, small size, and ease of use, they have been widely used to evaluate daily life walking

in recent years [22]. By using the In-Shoe Motion Sensor system (IMS), which measures the acceleration and angular velocity of the feet using IMUs and calculates walking parameters, it is possible to obtain walking data comparable to daily life more accurately than conventional studies– [23].

Therefore, this study aimed to evaluate the impact of high-heeled shoes on gait parameters in healthy adult women using the IMS system. Unlike previous studies that typically used treadmill walking at a constant speed or required participants to wear unfamiliar shoes, this study allowed participants to walk in their own footwear that fitted their feet and that they were accustomed to, under conditions similar to those of daily life. This approach enabled us to capture a more natural gait pattern, closely resembling everyday walking, and to more accurately reflect the effects of high-heeled shoes on gait parameters.

Materials and Methods

Study design

This was an intervention study.

Participants

The participants in this study were healthy adult women enrolled at Kyoto University. The inclusion criteria were individuals aged 18 years and older who could walk for at least 6 minutes

without rest. To minimize the influence of unfamiliarity with high-heeled shoes on the results, we selected participants with extensive experience wearing high heels in various settings, such as work, social events, shopping, or formal occasions, based on previous studies [14]. The exclusion criteria were individuals with neurological or orthopedic conditions that could affect walking function. The purpose of the study was thoroughly explained to the participants, and their consent was obtained prior to measurement.

Ethics statement

This study was approved by the Kyoto University Medical Ethics Committee (Approval number: R3664-3). Prior to study initiation, written informed consent was obtained from all participants in accordance with the ethical standards and guidelines.

Equipment

The IMS System (A-RROWG, NEC Corporation, Tokyo, Japan) was used for the measurements. The IMS is a device used for gait analysis and consists of key components including an ARM Cortex-M4F MCU (nRF52832 by Nordic Semiconductor). This component features a 64 MHz CPU, 64 KB of RAM, and 512 KB of ROM. Additionally, it includes a Bosch IMU (BMI160) for precise motion tracking, an ABLIC EEPROM (S-24C32C, 32K-bit) for storage, and an Epson real-

time clock (RX8130CE). This IMS maintains measurement accuracy by applying real-time correction through two algorithms: the Three-dimensional zero-velocity-update (3D ZUPT) algorithm and the On-line stride segmentation algorithm. The on-line stride segmentation algorithm enables stable foot-flat detection using the plantar angle, ensuring precise stride segmentation. Additionally, the 3D ZUPT algorithm effectively compensates for drift errors, allowing for accurate stride parameter computation within the IMS device [25]. This device performs gait measurements at 2-minute intervals, capturing gait parameters on both sides. The system detects the gait cycle during flat walking, operates for 1 min, and then enters sleep mode to conserve energy. During this 1-minute period, it detects vibrations and identifies stable walking patterns, and upon detecting the start of a gait cycle, it measures the parameters for three steps within the following 9 s. These steps do not need to be consecutive; if the three steps are successfully measured within a specified time, the average value is recorded. If fewer than three steps were measured, the average value was not recorded. Additionally, this measurement process can be repeated up to three times within a 1-minute window; if all three attempts fail, the measurement for that period is considered unsuccessful, and no data is recorded [25,26,27].

The following 16 parameters are calculated: Stride length, Maximum (peak) dorsiflexion angle, Maximum (peak) plantar flexion angle, Foot clearance, Foot height, Circumduction, Toe-in/out angle, Roll angle of heel contact, Roll angle of toe-off, Walking speed, Peak swing angular velocity,

Maximum speed during swinging phase, Stance time, Swing time, Pushing time, and Cadence. Table 1 below, disclosed by NEC Corporation, categorizes the definitions of each parameter into spatial, spatiotemporal, and temporal parameters. These parameters were validated in previous studies for accuracy or were derived from validated parameters [25,26,27].

High-heeled shoes were defined as “footwear with a heel elevated higher than the forefoot, a narrow toe box, and a curved sole” [11], while sneakers were defined as “light footwear that has a top made of cloth and a bottom made of rubber.”

Table 1. Definitions of Each Walking Parameter.

Classification	Parameters	Definition
**Spatial parameters**	Stride length (cm)	Distance from the starting point to end point of the foot trajectory
	Maximum (peak) dorsiflexion angle (degree)	Peak foot-sole angle in dorsiflexion
	Maximum (peak) plantar flexion angle (degree)	Peak foot-sole angle in plantarflexion
	Foot clearance (cm)	Maximum heel height of the foot trajectory

	Foot height (cm)	Maximum vertical height of the foot trajectory
	Circumduction (cm)	Displacement in the medial–lateral direction during the swing phase
	Toe-in/out angle (degree)	Average adduction/abduction angle during the swing phase
	Roll angle of heel contact (degree)	Plantar roll angle when the heel touches the ground
	Roll angle of toe-off (degree)	Plantar roll angle when the toe leaves the ground
**Spatiotemporal parameters**	Walking speed (km/h)	Stride length divided by stride time
	Peak swing angular velocity (degree/s)	Peak angular velocity of the foot during the swing phase
	Maximum speed during swinging phase (km/h)	Maximum forward speed of the swinging leg during the swinging phase
**Temporal parameters**	Stance time(s)	Amount of time that the foot is on the ground during the gait cycle

	Swing time(s)	Amount of time that the foot is off the ground during the gait cycle
	Pushing time (s)	Time between the heel contact and toe leaving the ground.
	Cadence (steps/min)	Number of steps per min

Procedure

The measurements were conducted in a 30-meter corridor at the Faculty of Human Health Sciences building, at Kyoto University. First, we verified whether the shoes worn by the participants met the aforementioned criteria. Subsequently, we measured the age (years), height (cm), body mass(kg), size of the shoes(cm), heel height (cm) of the high-heeled shoes, and mass (g) of each shoe. Prior to data collection, participants walked 5 m with insoles equipped with an IMS inserted into their shoes to ensure that they experienced no discomfort during walking. For data collection, Participants placed a smartphone, used for data recording, in either pocket of their pants, as there were no restrictions on which side to use. They were instructed to begin walking from one end of the corridor, turn around at the opposite end, and continue this back-and-forth walking for 6 min. The measurements were conducted twice: once in sneakers and once in high-heeled shoes, with the order of conditions determined using Excel’s random function. Participants were given the

option to take a break between trials upon request. The observer stood in the middle of the pathway during the test to ensure that the participants walked normally and without safety issues, providing updates on the remaining time every minute. If no data were collected, the 6-minute walking test was repeated.

Data analysis

Statistical analyses were performed using the JMP PRO16 software (SAS Institute). First, the Shapiro-Wilk test was conducted to determine whether each dataset followed a normal distribution. For data that were normally distributed, a paired t-test was used, because the same participants were measured under different conditions (high-heeled shoes and sneakers). For data that did not follow a normal distribution, the Wilcoxon signed-rank test, suitable for paired datasets, was applied. The group wearing high-heeled shoes was referred to as the "high-heeled group" (HG) and the group wearing sneakers as the "sneaker group" (SG). Data are presented as mean standard deviation ± standard deviation (SD). The effect size was evaluated using Cohen's d to assess the magnitude of the differences between groups, with thresholds defined as Small (0.2–0.49), Medium (0.5–0.79), and Large (≥ 0.8) [28]. The statistical significance level for this study was set at less than 5%.

Results

A. Physical Characteristics (Table 2)

There were 17 participants in the study, with no dropouts or adverse events reported. The mean values were as follows: age, 23.4 years; height, 161.78 cm; body mass, 53.46 kg; BMI, 20.38; size of shoes, 24.15 cm; mass of high-heeled shoes, 201.76 g; mass of sneakers, 265.00 g; heel height of high-heeled shoes, 4.93 cm (Table1).

Table 2. Basic Information of Participants.

Characteristics	Mean±SD(N=17)
Age(years)	23.40±1.23
Height(cm)	161.78±6.14
Body Mass(kg)	53.46±5.61
BMI(kg/m^2)	20.38±1.09
Size of shoes(cm)	24.15±1.01
Mass of heel shoes(g)	201.76±37.12
Mass of sneakers(g)	265.00±59.48
Heel height of heel shoes(cm)	4.93±1.17

SD, standard deviation; BMI, body mass index

B. Comparison of Walking Parameters Between High-Heeled and Sneaker Shoes (Table 3)

From the table 3, in the comparison between the high-heeled group and the sneaker group, spatial parameters showed that the high-heeled group had a significantly shorter stride length, reduced peak dorsiflexion angle of the foot, reduced peak plantar flexion angle of the foot, and significantly greater toe angle compared to the sneaker group. Regarding spatiotemporal parameters, the high-heeled group exhibited significantly reduced walking speed and maximum speed during swinging phase. There were no significant differences observed between the groups in temporal parameters.

Table 3. Comparisons of the mean values of each parameter in each category.

Classification	Parameters	HG	SG	P-value	Effect Size (Cohen's d)
**Spatial parameters**	Stride length (cm)	130.42±18.14	143.88±16.09	0.0006*	-1.09
	Maximum (peak) dorsiflexion angle (degree)	24.21±5.25	29.88±5.13	0.0017*	-0.94

	Maximum (peak) plantar flexion angle (degree)	62.33±11.15	78.31±6.49	<0.0001*	-1.47
	Foot clearance (cm)	21.17±3.31	24.05±2.15	0.0005*	-1.09
	Foot height (cm)	13.33±2.40	15.70±1.76	0.0023*	-0.90
	Circumduction (cm)	4.10±1.58	3.72±2.01	0.5397	0.15
	Toe-in/out angle (degree)	12.05±8.82	10.14±7.97	0.0476*	0.54
	Roll angle of heel contact (degree)	5.21±4.86	3.71±5.70	0.2343	0.30
	Roll angle of toe-off (degree)	-2.05±6.17	-1.36±4.52	0.5178	-0.17
**Spatiotemporal parameters**	Walking speed (km/h)	4.61±0.68	5.08±0.66	0.0005*	-1.09
	Peak swing angular velocity (degree/s)	531.64±95.96	565.64±75.54	0.0932	-0.45

	Maximum speed during swinging phase (km/h)	15.15±1.65	15.97±1.59	0.0085*	-0.75
**Temporal parameters**	Stance time(s)	0.62±0.05	0.62±0.04	0.8271	-0.06
	Swing time(s)	0.41±0.04	0.40±0.01	0.3225	0.03
	Pushing time (s)	0.19±0.03	0.20±0.02	0.6805	-0.13
	Cadence (steps/min)	117.22±6.41	116.92±5.69	0.8030	0.06

SD, standard deviation. *$p<0.05$.

Effect size (Cohen's d): Small = 0.2–0.49, Medium = 0.5–0.79, Large ≥ 0.8

Discussion

This study aimed to evaluate the effect of high-heeled shoes on gait parameters in healthy adult women using an IMS system. In previous studies, participants often wore unfamiliar shoes and measurements were commonly conducted on a treadmill at a constant speed, which may not accurately reflect natural gait patterns in real-life settings. In contrast, this study utilized a compact and user-friendly IMS to collect gait data while participants wore their own familiar shoes. This

approach allowed for accurate collection in a manner closer to daily life conditions, providing foundational data for assessing the effects of footwear under more realistic circumstances.

As a result, in terms of spatial parameters, the high-heeled group showed significantly shorter stride length, reduced maximum dorsiflexion angle, reduced maximum plantar flexion angle, and significantly greater toe-out angle compared to the sneaker group, with effect sizes ranging from moderate to high. Additionally, in spatiotemporal parameters, the high-heeled group exhibited significantly reduced walking speed and maximum speed during the swinging phase, with large effect sizes. However, no significant differences were observed between the groups in temporal parameters.

Regarding spatial parameters such as walking speed, stride length, and maximum swing speed, it is generally known that wearing high-heeled shoes reduces the base of support during walking, prompting individuals to adopt a safer and more stable gait pattern [29,30]. Additionally, when wearing high heels, the reduced base of support during walking leads to increased muscle activity to maintain balance, which in turn tends to increase energy expenditure. To minimize this increase in energy expenditure, it has been shown that walking speed and stride length are reduced as a strategy [31,32]. In addition to these stable and efficient walking strategies, previous studies have shown that wearing high heels brings the ground reaction force closer to the ankle joint, and the curved shape of the shoe sole causes the ankle to remain in a plantarflexed position [11]. These factors can

shorten the length of the triceps surae muscle and Achilles tendons. Consequently, compared to sneakers or barefoot walking, ankle plantarflexion moments decrease, and the effect of the stretch-shortening cycle on the muscle-tendon complex is reduced [33,34]. The stretch-shortening cycle is a process in which muscles and tendons quickly stretch and then shorten, store, and release elastic energy, and plays a crucial role in efficient force production [34]. However, the shortened state of the muscle-tendon complex induced by wearing high heels is thought to impede this function, leading to decreased energy efficiency and a weaker push-off force. As a result, the calculated parameters were significantly different between the two groups.

Similarly, foot clearance and foot height were significantly reduced in the high-heeled group. As noted above, wearing high-heeled shoes is suggested to reduce the push-off force of the ankle. Additionally, previous studies investigating the role of the ankle in walking have shown that ankle push-off contributes to up to 50% of the power needed for leg swinging during the terminal stance and early swing phases [35,36,37]. This reduction in ankle push-off may decrease the leg swing speed, which is essential for the double-pendulum effect during walking [38]. In the double-pendulum effect, an increased hip swing speed results in the relative lifting of the lower leg, consequently increasing foot clearance. This occurs as the hip and knee joints work in coordination, with the hip’s movement influencing the lower leg’s motion. Specifically, a rapid change in the hip angle causes the lower leg to rise, allowing the foot to clear obstacles before making contact with

the ground. In the heel group, however, a reduction in leg swing speed leads to insufficient knee flexion in the double-pendulum mechanism, causing the lower leg to remain relatively low and potentially resulting in reduced foot clearance.

In fact, prior research comparing joint angles in the sagittal plane under different heel heights at a constant walking speed found that while hip flexion during the swing phase remained unaffected by heel height, the knee flexion angle significantly decreased as heel height increased [39,40,41]. Moreover, a decrease in the foot clearance height can increase the risk of tripping over small obstacles or uneven surfaces while walking, potentially leading to falls [42]. High-heeled shoes also tilt the toes downward and elevate the heel, reducing the contact area of the foot with the ground, which may further destabilize balance. This combination of reduced foot clearance and a limited base of support likely exacerbates the risk of falls [14].

Thus, the high-heeled group showed significantly reduced stride length, reduced peak dorsiflexion angle of the foot, reduced peak plantar flexion angle of the foot, reduced walking speed, and swing phase maximum velocity, while the toe angle during the swing phase was significantly greater. A greater toe angle indicates a larger ankle abduction angle. Studies investigating the influence of heel height on the anterior-posterior joint moments of the reduced limb have shown that as heel height increases, knee internal rotation moments increase, potentially increasing the load on the medial compartment of the knee [9,43]. Conversely, walking with toes

pointing outward reduces knee internal rotation moments and decreases the load on the medial compartment [44]. In this study, it is suggested that the high-heeled group adopted a walking strategy with toes pointing outward to reduce the load on the medial compartment of the knee. Additionally, since knee joint movements during stance and toe direction were not measured in this study, further experiments are necessary.

There are limitations in this study; the participants were limited to healthy adult women from the same university, which restricts the generalizability of our findings. To apply these results to other age groups, sexes, or individuals with specific health conditions, further studies with more diverse participants are necessary. Second, the sample size in this study was limited to 17 participants, which poses constraints on statistical robustness and the power to detect effects. In particular, to adequately assess the impact of individual differences, it is essential to include a larger number of participants. Future studies should increase the sample size to enhance statistical robustness and improve the power to detect effects. Finally, we were unable to capture the movements of the hip and knee joints, making it challenging to fully assess how these joints affect gait mechanics, particularly balance and posture control. Specifically, it remains unclear to what extent high-heeled shoes stress on these joints under near-daily conditions and how this stress might increase the risk of falls or lead to long-term joint disorders. Future research should incorporate devices that measure

the movements of these joints to provide a more comprehensive evaluation of the impact of different footwear types on gait and balance.

Conclusions

This study investigated the effects of high-heeled shoes on walking parameters in healthy adult women using the IMS. As a result, in terms of spatial parameters, the high-heeled group showed significantly shorter stride length, reduced maximum dorsiflexion angle, reduced maximum plantar flexion angle, and significantly greater toe-out angle compared to the sneaker group, with effect sizes ranging from moderate to high. Additionally, in spatiotemporal parameters, the high-heeled group exhibited significantly reduced walking speed and maximum speed during the swinging phase, with large effect sizes. However, no significant differences were observed between the groups in temporal parameters. These findings suggest that wearing high-heeled shoes has a substantial impact on gait stability and efficiency, potentially increasing the risk of falls and musculoskeletal strain. Future studies should include a more diverse population, such as older adults or individuals with joint disorders, and incorporate motion capture systems or wearable sensors that can measure reduced limb joint dynamics. These additional studies would allow for a comprehensive understanding of how footwear affects not only the ankle but also hip and knee joint movements across a wider population. Because this study collected gait data under conditions

similar to those of daily life, it provides data suitable for practical applications and may contribute to future research focused on evaluating gait in everyday scenarios.

Acknowledgments

We express our gratitude to all participants, instructors, and laboratory members for their cooperation and valuable input throughout this study. We also thank Editage (www.editage.com) for English editing.

References

1. Prince F, Corriveau H., Hebert R, Winter DA. Gait in elderly. Gait & Posture. 1997;5(2): 128-135. doi: 10.1016/S0966-6362(97)01118-1
2. Bai X, Kim GS, Dev RDO, Talib O, Xiao W, Cai H. Effect of Brisk Walking on Health-Related Physical Fitness Balance and Life Satisfaction Among the Elderly: A Systematic Review. Frontiers in Public Health. 2022;9. doi: https://doi.org/10.3389/fpubh.2021.829367
3. Menant JC, Steele JR, Menz HB, Munro BJ, Lord SR. Effects of walking surfaces and footwear on temporo-spatial gait parameters in young and older people. Gait & Posture. 2009;29(3):392-397. doi: https://doi.org/10.1016/j.gaitpost.2008.10.057

4. Barton CJ, Bonanno D, Menz HB. Development and evaluation of a tool for the assessment of footwear characteristics. Journal of Foot Ankle Research. 2009;2(10). doi: 10.1186/1757-1146-2-10
5. Shakoor N, Block JA. Walking Barefoot Decreases Loading on the Lower Extremity Joints in Knee Osteoarthritis. ARTHRITIS & RHEUMATISM. 2006;54(9):2923-2927. doi: 10.1002/art.22123
6. Keenan GS, Franz JR, Dicharry J, Della Croce U, Casey Kerrigan D. Lower limb joint kinetics in walking: The role of industry recommended footwear. Gait & Posture. 2011;33(3):350-355. doi: 10.1016/j.gaitpost.2010.09.019
7. Luximon Y, Cong Y, Luximon A, Zhang M. Effects of heel base size, walking speed, and slope angle on center of pressure trajectory and plantar pressure when wearing high-heeled shoes. Human Movement Science. 2015;41;307-319. doi: 10.1016/j.humov.2015.04.003
8. Zeng Z, Liu Y, Hu X, Li P, Wang L. Effects of high-heeled shoes on lower extremity biomechanics and balance in females: a systematic review and meta-analysis. BMC Public Health. 2023;23(726). doi: 10.1186/s12889-023-15641-8
9. Barkema DD, Derrick TR, Martin PE. Heel height affects lower extremity frontal plane joint moments during walking. Gait & Posture. 2012;35(3):483-488. doi: 10.1016/j.gaitpost.2011.11.013

10. Xavier MJ, Lambert B, Jenkins GP, McGwin Jr G. Epidemiology of High-Heel Shoe Injuries in U.S. Women: 2002 to 2012. ORIGINAL RESEARCH. 2015;54(4):615-619. doi: 10.1053/j.jfas.2015.04.008
11. Cronin NJ. The effects of high heeled shoes on female gait: A review. Journal of Electromyography and Kinesiology. 2014;24(2):258-263. doi:10.1016/j.jelekin.2014.01.004
12. Alkjaer T, Raffalt P, Petersen NC, Simonsen EB. Movement Behavior of High-Heeled Walking: How Does the Nervous System Control the Ankle Joint during an Unstable Walking Condition? PLoS ONE. 2012;7(5). doi: https://doi.org/10.1371/journal.pone.0037390
13. Falola JM, Koussihouèdé FEN, Falola SMD, Avossevou YG, Bio NI, Dansou HP. Effect of the Base of the Shoe Heel on Postural Stability During Walking in Women. American Journal of BioScience. 2015;3(5):167-170. doi: 10.11648/j.ajbio.20150305.11
14. Annoni I, Mapelli A, Sidequersky FV, Zago M, Sforza C. The effect of high-heeled shoes on overground gait kinematics in young healthy women. Sport Sciences for Health. 2014;10(2):149-157. doi: 10.1007/s11332-014-0191-z
15. Sloot LH, Van der Krogt MM, Harlaar J. Effects of adding a virtual reality environment to different modes of treadmill walking. Gait & Posture. 2014;39(3): 939-945. doi: https://doi.org/10.1016/j.gaitpost.2013.12.005

16. White SC, Yack HJ, Tucker CA, Lin HY. Comparison of vertical ground reaction forces during overground and treadmill walking. Medicine & Science in Sports & Exercise. 1998;30(10): 1537–1542. doi:10.1097/00005768-199810000-00011

17. Stolze H, Kuhtz-Buschbeck JP, Mondwurf C, Boczek-Funcke A, Jöhnk K, Deuschl G, Illert M. Gait analysis during treadmill and overground locomotion in children and adults. Electroencephalography and Clinical Neurophysiology. 1997;105(6): 490–497. doi:10.1016/s0924-980x(97)00055-6

18. Menz HB, Bonanno DR. Footwear comfort: a systematic search and narrative synthesis of the literature. Journal of Foot and Ankle Research. 2021;14:63. doi:https://doi.org/10.1186/s13047-021-00500-9

19. O'Rourke B, Walsh ME, Brophy R, Vallely S, Murphy N, Conroy B, et al. Does the shoe really fit? Characterising ill-fitting footwear among community-dwelling older adults attending geriatric services: an observational cross-sectional study. BMC Geriatrics. 2020; 20: 55. doi:https://doi.org/10.1186/s12877-020-1448-9

20. Aminian K, Renzakhanlou K, De Andres E, Fritsch C, Leyvraz PF, Robert P. Temporal feature estimation during walking using miniature accelerometers: an analysis of gait improvement after hip arthroplasty. Medical & Biological Engineering & Computing. 1999;37(6):686-691. doi: 10.1007/BF02513368

21. Jasiewicz Jan M, Allum John HJ, Middleton James W, Barriskill Andrew, Condie Peter, Pucell Brendan, et al. Gait event detection using linear accelerometers or angular velocity transducers in able-bodied and spinal-cord injured individuals. Gait & Posture. 2006;24(4):502-509. doi: https://doi.org/10.1016/j.gaitpost.2005.12.017

22. Anwary AR, Yu H, Vassallo M. Optimal Foot Location for Placing Wearable IMU Sensors and Automatic Feature Extraction for Gait Analysis. IEEE Sensors Journal. 2018;18(6):2555-2567. doi: 10.1109/JSEN.2017.2786587

23. Yamamoto A, Fujita K, Yamada E, Ibara T, Nihey F, Inai T, et al. Foot characteristics of the daily-life gait in postmenopausal females with distal radius fractures: a cross-sectional study. BMC Musculoskeletal Disorders. 2023;24(1). doi: 10.1186/s12891-023-06845-5

24. Chard A, Greene A, Hunt A, Vanwanseele B, Smith R. Effect of thong style flip-flops on children's barefoot walking and jogging kinematics. Journal of Foot and Ankle Research. 2013;6:8. doi:https://doi.org/10.1186/1757-1146-6-8

25. Fukushi K, Huang C, Wang Z, Kajitani H, Nihey F, Nakahara K. On-line algorithms of stride-parameter estimation for in-shoe motion-sensor system. IEEE Sensors Journal. 2022;22:9636–9648. doi:https://doi.org/10.1109/JSEN.2022.3164057

26. Huang C, Fukushi K, Wang Z, Nihey F, Kajitani H, Nakahara K. An algorithm for real time minimum toe clearance estimation from signal of in-shoe motion sensor. Annual International

Conference of the IEEE Engineering in Medicine and Biology Society. 2021: 6775–6778. doi:https://doi.org/10.1109/EMBC46164.2021.9629875

27. Huang C, Fukushi K, Wang Z, Nihey F, Kajitani H, Nakahara K. Method for estimating temporal gait parameters concerning bilateral lower limbs of healthy subjects using a single in-shoe motion sensor through a gait event detection approach. Sensors (Basel). 2022;22(1):351. doi:https://doi.org/10.3390/S22010351

28. Cohen J. Statistical power analysis for the behavioral sciences. 2nd ed. Lawrence Erlbaum Associates; 1988. doi: https://doi.org/10.4324/9780203771587

29. Esenyel M, Walsh K, Walden JG, Gitter A. Kinetics of high-heeled gait. Journal of the American Podiatric Medical Association. 2003;93(1):27-32. doi: 10.7547/87507315-93-1-27

30. Lee CM, Jeong EH, Freivalds A. Biomechanical effects of wearing high-heeled shoes. International Journal of Industrial Ergonomics. 2001;28(6): 321–326. doi:https://doi.org/10.1016/S0169-8141(01)00038-5

31. Ebbeling CJ, Hamill J, Crussemeyer JA. Lower extremity mechanics and energy cost of walking in high-heeled shoes. Journal of Orthopaedic & Sports Physical Therapy. 1994;19(4): 190–196. doi:https://doi.org/10.2519/jospt.1994.19.4.190

32. Curran SA, Holliday JL, Watkeys L. Influence of high-heeled footwear and pre-fabricated orthoses on energy efficiency in ambulation. The Foot and Ankle Online Journal. 2010;3(3): 1–11. doi:https://doi.org/10.3827/faoj.2010.0303.0001

33. Komi PV. Stretch-shortening cycle: a powerful model to study normal and fatigued muscle. Journal of Biomechanics. 2000;33(10): 1197–1206. doi:https://doi.org/10.1016/S0021-9290(00)00064-6

34. Roberts TJ, Azizi E. Flexible mechanisms: the diverse roles of biological springs in vertebrate movement. Journal of Experimental Biology. 2011;214(3): 353–361. doi:https://doi.org/10.1242/jeb.038588

35. Fickey SN, Browne MG, Franz JR. Biomechanical effects of augmented ankle power output during human walking. Journal of Experimental Biology. 2018;221(22): jeb182113. doi:https://doi.org/10.1242/jeb.182113

36. Neptune RR, Clark DJ, Kautz SA. Modular control of human walking: A simulation study. Journal of Biomechanics. 2009;42(9): 1282–1287. doi:https://doi.org/10.1016/j.jbiomech.2009.03.009

37. Meinders M, Gitter A, Czerniecki JM. 1998; The role of ankle plantar flexor muscle work during walking. Scandinavian Journal of Rehabilitation Medicine. 30(1): 39–46. doi:https://doi.org/10.1080/003655098444309

38. Cinto F, Nacusse M, Haimovich H. Making a double pendulum walk: from compass- to whip-like gait. 2022 IEEE Biennial Congress of Argentina (ARGENCON). 2022;pp.1–8. doi:https://doi.org/10.1109/ARGENCON55245.2022.9939939

39. Snow RE, Williams KR. High Heeled Shoes: Their Effect on Center of Mass Position, Posture, Three-Dimensional Kinematics, Rearfoot Motion, and Ground Reaction Forces. Archives of Physical Medicine and Rehabilitation. 1994;75(5):568-576. doi: https://doi.org/10.1016/S0003-9993(21)01622-1

40. Opila KA. Kinematics of high-heeled gait. Archives of Physical Medicine and Rehabilitation. 1990;71(5):304-309. doi: 10.7547/87507315-93-1-27

41. Gehlsen G, Braarz JS, Assmann N. Effects of heel height on knee rotation and gait. Human Movement Science. 1986;5(2):149-155. doi: https://doi.org/10.1016/0167-9457(86)90021-7

42. Dadashi F, Mariani B, Rochat S, Bula CJ, Santos-Eggiman, B, Aminian K. Gait and Foot Clearance Parameters Obtained Using Shoe-Worn Inertial Sensors in a Large-Population Sample of Older Adults. Sensors(Basel). 2014;14(1):443-457. doi: 10.3390/s140100443

43. Teichtahl AJ, Morrie ME, Wluka AE, Baker R, Wolfe R, Davis SR, et al. Foot rotation-a potential target to modify the knee adduction moment. Journal of Science and Medicine in Sport. 2006;9(1-2):67-71. doi: https://doi.org/10.1016/j.jsams.2006.03.011

44. Chang A, Hurwitz D, Dunlop D, Song J, Cahue S, Hayes K, et al. The relationship between toe-out angle during gait and progression of medial tibiofemoral osteoarthritis. Annals of the Rheumatic Diseases. 2007;66(10):1271-1275. doi: 10.1136/ard.2006.062927

Click here to access/download
Supporting Information
Raw Data for Repository Ver3.xlsx

The Effects of High-Heeled Shoes on Gait Parameters in Healthy Adult Women.

Short Title: The effects of high-heeled shoes on gait parameters

Kyoma Tanigawa[a], Hiroki Shimizu[a], Anuradhi Bandara[a], Toyota Misa[a], Shota Suzuki[a], Momoko Nagai-Tanima[a], Tomoki Aoyama*[a]

[a]Department of Physical Therapy, Human Health Sciences, Graduate School of Medicine, Kyoto University, Kyoto, 606-8507, Japan

*Corresponding author: Tomoki Aoyama

E-mail: aoyama.tomoki.4e@kyoto-u.ac.jp

Abstract

Background

Walking is essential in daily life, and footwear type significantly affects walking patterns. High-heeled shoes increase the risk of knee osteoarthritis and falls in women. Traditional studies often use treadmills or unfamiliar footwear, which may not reflect daily walking. This study ~~investigates~~ investigated the impact of high-heeled shoes on walking parameters in healthy adult women using in-shoe motion sensors.

Methods

Seventeen healthy adult women without pain during walking participated. They walked for 6 minutes along a 30-meter corridor wearing high-heeled shoes and sneakers. Walking data were recorded using an in-shoe motion sensor system every 2 minutes. The average of three valid consecutive steps was calculated automatically. Statistical analysis compared the mean walking parameters between the high-heel and sneaker groups.

Results

The high-heel group showed significantly ~~lower~~ reduced foot clearance, stride length, peak plantar angle in dorsiflexion, and peak plantar angle in plantarflexion, and significantly ~~higher~~ greater toe-out angle in spatial parameters. Spatiotemporal parameters revealed significantly ~~lower~~ reduced walking speed and maximum

swing phase speed in the high-heel group. No significant differences were observed in temporal parameters between the groups.

Conclusions

Since this study collected gait data under conditions similar to daily life, it provides data suitable for practical applications and may contribute to future research evaluating everyday gait. Additionally, future studies should include a broader range of participants and incorporate measurement devices capable of capturing hip and knee joint movements, providing a more comprehensive evaluation of the effects of high-heeled shoes on gait in healthy adult women.

Introduction

Walking is a crucial means of transporting the body safely and efficiently [1]. Additionally, it has beneficial effects on both physical and mental health, enhances human quality of life (QOL), and plays a significant role in the prevention and treatment of various diseases [2]. As such, walking is indispensable in daily life. It is also known that walking is influenced by various external factors [3].

One of these external factors is footwear. Shoes come in various types and have multiple functions, including protecting the feet from external environments, assisting walking functions, and adapting to foot deformities. These functions are related to the occurrence of various conditions

such as falls, osteoarthritis, and back pain [4]. Numerous studies have investigated the impact of shoes on walking parameters [5,6]. For example, research comparing walking barefoot and wearing athletic shoes by Shakoor et al. found that wearing athletic shoes decreases step length, increases the number of steps, and reduces joint angles of the lower limbs [5]. Thus, research on how shoes affect walking is of great importance [7].

One type of shoe that warrants attention is high-heeled shoes. High-heeled shoes are characterized by heels higher than the forefoot, narrow toe boxes, and curved soles [8]. High-heeled shoes have been widely worn by women worldwide for centuries, with 37% to 69% of women wearing them regularly and for an average of 1 to 8 hours per day [8]. Despite their regular use, high-heeled shoes are associated with medical conditions, such as osteoarthritis, and can cause frequent falls [9]. The incidence of such injuries doubled from 7.1% to 14.2% between 2002 and 2012 [10]. Therefore, it is crucial to investigate the impact of high-heeled shoes on the walking parameters of healthy adult women [7]. Several previous studies have examined this effect [9,11,12,13]. For instance, it has been shown that wearing high-heeled shoes does not change the temporal walking parameters such as stance time and cadence [11]. Additionally, a study by Barkema et al. noted that the higher the heel height, the greater the maximum knee adduction moment, indicating a relationship between heel height and knee osteoarthritis [9].

Research on the effects of high-heeled shoes on gait parameters has significantly progressed. However, many evaluations have been conducted using a treadmill with participants walking at a constant speed [14]. Treadmill walking occurs under flat, steady-speed conditions, which differs from everyday walking environments [15]. For example, because a treadmill maintains a fixed speed, participants cannot walk at their natural pace, potentially suppressing their typical gait patterns [16,17]. Additionally, on a treadmill, individuals often need to adjust their posture and gait mechanics to match the treadmill's motion in order to maintain visual and balance cues, which is known to influence gait biomechanics [15]. In certain studies, participants wore unfamiliar shoes that may not accurately reflect their usual gait patterns in daily life [11,14]. Shoes that are unfamiliar or uncomfortable for an individual often do not fit well with the person's foot shape or gait style, potentially affecting the gait biomechanics [18]. Specifically, when the shoe's size or shape does not align with the foot, it may cause foot pain and fatigue, while also reducing stability and increasing the risk of falls during walking [19]. Therefore, conditions different from those in daily life may alter an individual's natural gait patterns and may not accurately reflect habitual walking [11].

As a more suitable evaluation method, Inertial Measurement Units (IMUs) can be used. IMUs can estimate three-dimensional movement from angular velocity and acceleration [20,21]. Due to their low cost, small size, and ease of use, they have been widely used to evaluate daily life walking

in recent years [22]. By using the In-Shoe Motion Sensor system (IMS), which measures the acceleration and angular velocity of the feet using IMUs and calculates walking parameters, it is possible to obtain walking data comparable to daily life more accurately than conventional studies–[23].

Therefore, this study aimed to evaluate the impact of high-heeled shoes on gait parameters in healthy adult women using the IMS system. Unlike previous studies that typically used treadmill walking at a constant speed or required participants to wear unfamiliar shoes, this study allowed participants to walk in their own footwear that fitted their feet and that they were accustomed to, under conditions similar to those of daily life. This approach enabled us to capture a more natural gait pattern, closely resembling everyday walking, and to more accurately reflect the effects of high-heeled shoes on gait parameters.

Materials and Methods

Study design

This was an intervention study.

Participants

The participants in this study were healthy adult women enrolled at Kyoto University. The inclusion criteria were individuals aged 18 years and older who could walk for at least 6 minutes

without rest. To minimize the influence of unfamiliarity with high-heeled shoes on the results, we selected participants with extensive experience wearing high heels in various settings, such as work, social events, shopping, or formal occasions, based on previous studies [14]. The exclusion criteria were individuals with neurological or orthopedic conditions that could affect walking function. The purpose of the study was thoroughly explained to the participants, and their consent was obtained prior to measurement. ~~The sample size for this study was calculated using G Power 3.1 (Heinrich Heine University). Based on prior research [24], with an assumed effect size of 0.62, an alpha level of 0.05, and a statistical power of 0.8, the minimum required number of participants was calculated to be 15. To account for potential data loss or incomplete data, an additional five participants were included, setting the final target number of participants at 20.~~

Ethics statement

This study was approved by the Kyoto University Medical Ethics Committee (Approval number: R3664-3). Prior to study initiation, written informed consent was obtained from all participants in accordance with the ethical standards and guidelines.

Equipment

The IMS System (A-RROWG, NEC Corporation, Tokyo, Japan) was used for the measurements. The IMS is a device used for gait analysis and consists of key components including an ARM Cortex-M4F MCU (nRF52832 by Nordic Semiconductor). This component features a 64 MHz CPU, 64 KB of RAM, and 512 KB of ROM. Additionally, it includes a Bosch IMU (BMI160) for precise motion tracking, an ABLIC EEPROM (S-24C32C, 32K-bit) for storage, and an Epson real-time clock (RX8130CE). This IMS maintains measurement accuracy by applying real-time correction through two algorithms: the Three-dimensional zero-velocity-update (3D ZUPT) algorithm and the On-line stride segmentation algorithm. The on-line stride segmentation algorithm enables stable foot-flat detection using the plantar angle, ensuring precise stride segmentation. Additionally, the 3D ZUPT algorithm effectively compensates for drift errors, allowing for accurate stride parameter computation within the IMS device [25]. This device performs gait measurements at 2-minute intervals, capturing gait parameters on both sides. The system detects the gait cycle during flat walking, operates for 1 min, and then enters sleep mode to conserve energy. During this 1-minute period, it detects vibrations and identifies stable walking patterns, and upon detecting the start of a gait cycle, it measures the parameters for three steps within the following 9 s. These steps do not need to be consecutive; if the three steps are successfully measured within a specified time, the average value is recorded. If fewer than three steps were measured, the average value was not recorded. Additionally, this measurement process can be repeated up to three times within a 1-

minute window; if all three attempts fail, the measurement for that period is considered unsuccessful, and no data is recorded [25,26,27].

The following 16 parameters are calculated: Stride length, Maximum (peak) dorsiflexion angle, Maximum (peak) plantar flexion angle, Foot clearance, Foot height, Circumduction, Toe-in/out angle, Roll angle of heel contact, Roll angle of toe-off, Walking speed, Peak swing angular velocity, Maximum speed during swinging phase, Stance time, Swing time, Pushing time, and Cadence. Table 1 below, disclosed by NEC Corporation, categorizes the definitions of each parameter into spatial, spatiotemporal, and temporal parameters. These parameters were validated in previous studies for accuracy or were derived from validated parameters [25,26,27].

High-heeled shoes were defined as "footwear with a heel elevated higher than the forefoot, a narrow toe box, and a curved sole" [11], while sneakers were defined as "light footwear that has a top made of cloth and a bottom made of rubber."

Table 1. Definitions of Each Walking Parameter.

Classification	Parameters	Definition
**Spatial parameters**	Stride length (cm)	Distance from the starting point to end point of the foot trajectory

	Maximum (peak) dorsiflexion angle (degree)	Peak foot-sole angle in dorsiflexion
	Maximum (peak) plantar flexion angle (degree)	Peak foot-sole angle in plantarflexion
	Foot clearance (cm)	Maximum heel height of the foot trajectory
	Foot height (cm)	Maximum vertical height of the foot trajectory
	Circumduction (cm)	Displacement in the medial–lateral direction during the swing phase
	Toe-in/out angle (degree)	Average adduction/abduction angle during the swing phase
	Roll angle of heel contact (degree)	Plantar roll angle when the heel touches the ground
	Roll angle of toe-off (degree)	Plantar roll angle when the toe leaves the ground
**Spatiotemporal parameters**	Walking speed (km/h)	Stride length divided by stride time

	Peak swing angular velocity (degree/s)	Peak angular velocity of the foot during the swing phase
	Maximum speed during swinging phase (km/h)	Maximum forward speed of the swinging leg during the swinging phase
**Temporal parameters**	Stance time(s)	Amount of time that the foot is on the ground during the gait cycle
	Swing time(s)	Amount of time that the foot is off the ground during the gait cycle
	Pushing time (s)	Time between the heel contact and toe leaving the ground.
	Cadence (steps/min)	Number of steps per min

Procedure

The measurements were conducted in a 30-meter corridor at the Faculty of Human Health Sciences building, at Kyoto University. First, we verified whether the shoes worn by the participants met the aforementioned criteria. Subsequently, we measured the age (years), height (cm), body mass(kg), size of the shoes(cm), heel height (cm) of the high-heeled shoes, and mass (g)

of each shoe. Prior to data collection, participants walked 5 m with insoles equipped with an IMS inserted into their shoes to ensure that they experienced no discomfort during walking. For data collection, Participants placed a smartphone, used for data recording, in either pocket of their pants, as there were no restrictions on which side to use. They were instructed to begin walking from one end of the corridor, turn around at the opposite end, and continue this back-and-forth walking for 6 min. The measurements were conducted twice: once in sneakers and once in high-heeled shoes, with the order of conditions determined using Excel's random function. Participants were given the option to take a break between trials upon request. The observer stood in the middle of the pathway during the test to ensure that the participants walked normally and without safety issues, providing updates on the remaining time every minute. If no data were collected, the 6-minute walking test was repeated.

Data analysis

Statistical analyses were performed using the JMP PRO16 software (SAS Institute). First, the Shapiro-Wilk test was conducted to determine whether each dataset followed a normal distribution. For data that were normally distributed, a paired t-test was used, because the same participants were measured under different conditions (high-heeled shoes and sneakers). For data that did not follow a normal distribution, the Wilcoxon signed-rank test, suitable for paired datasets, was applied. The

group wearing high-heeled shoes was referred to as the “high-heeled group” (HG) and the group wearing sneakers as the “sneaker group” (SG). Data are presented as mean standard deviation ± standard deviation (SD). ~~The effect size was evaluated using Cohen’s d to assess the magnitude of the differences between groups.~~ The effect size was evaluated using Cohen’s d to assess the magnitude of the differences between groups, with thresholds defined as Small (0.2–0.49), Medium (0.5–0.79), and Large (≥ 0.8) [28]. The statistical significance level for this study was set at less than 5%.

Results

A. Physical Characteristics (Table 2)

There were 17 participants in the study, with no dropouts or adverse events reported. The mean values were as follows: age, 23.4 years; height, 161.78 cm; body mass, 53.46 kg; BMI, 20.38; size of shoes, 24.15 cm; mass of high-heeled shoes, 201.76 g; mass of sneakers, 265.00 g; heel height of high-heeled shoes, 4.93 cm (Table1).

Table 2. Basic Information of Participants.

Characteristics	Mean±SD(N=17)
Age(years)	23.40±1.23
Height(cm)	161.78±6.14

Body Mass(kg)	53.46±5.61
BMI(kg/m^2)	20.38±1.09
Size of shoes(cm)	24.15±1.01
Mass of heel shoes(g)	201.76±37.12
Mass of sneakers(g)	265.00±59.48
Heel height of heel shoes(cm)	4.93±1.17

SD, standard deviation; BMI, body mass index

B. Comparison of Walking Parameters Between High-Heeled and Sneaker Shoes (Table 3)

From the table 3, in the comparison between the high-heeled group and the sneaker group, spatial parameters showed that the high-heeled group had a significantly shorter stride length, ~~lower~~ reduced peak dorsiflexion angle of the foot, ~~lower~~ reduced peak plantar flexion angle of the foot, and significantly ~~higher~~ greater toe angle compared to the sneaker group. Regarding spatiotemporal parameters, the high-heeled group exhibited significantly ~~lower~~ reduced walking speed and maximum speed during swinging phase. There were no significant differences observed between the groups in temporal parameters.

Table 3. Comparisons of the mean values of each parameter in each category.

Classification	Parameters	HG	SG	P-value	Effect Size (Cohen's d)
**Spatial parameters**	Stride length (cm)	130.42±18.14	143.88±16.09	0.0006*	-1.09
	Maximum (peak) dorsiflexion angle (degree)	24.21±5.25	29.88±5.13	0.0017*	-0.94
	Maximum (peak) plantar flexion angle (degree)	62.33±11.15	78.31±6.49	<0.0001*	-1.47
	Foot clearance (cm)	21.17±3.31	24.05±2.15	0.0005*	-1.09
	Foot height (cm)	13.33±2.40	15.70±1.76	0.0023*	-0.90
	Circumduction (cm)	4.10±1.58	3.72±2.01	0.5397	0.15
	Toe-in/out angle (degree)	12.05±8.82	10.14±7.97	0.0476*	0.54
	Roll angle of heel contact (degree)	5.21±4.86	3.71±5.70	0.2343	0.30

	Roll angle of toe-off (degree)	-2.05±6.17	-1.36±4.52	0.5178	-0.17
**Spatiotemporal parameters**	Walking speed (km/h)	4.61±0.68	5.08±0.66	0.0005*	-1.09
	Peak swing angular velocity (degree/s)	531.64±95.96	565.64±75.54	0.0932	-0.45
	Maximum speed during swinging phase (km/h)	15.15±1.65	15.97±1.59	0.0085*	-0.75
**Temporal parameters**	Stance time(s)	0.62±0.05	0.62±0.04	0.8271	-0.06
	Swing time(s)	0.41±0.04	0.40±0.01	0.3225	0.03
	Pushing time (s)	0.19±0.03	0.20±0.02	0.6805	-0.13
	Cadence (steps/min)	117.22±6.41	116.92±5.69	0.8030	0.06

SD, standard deviation. *p<0.05.

Effect size (Cohen's d): Small = 0.2–0.49, Medium = 0.5–0.79, Large ≥ 0.8

Discussion

This study aimed to evaluate the effect of high-heeled shoes on gait parameters in healthy adult women using an IMS system. In previous studies, participants often wore unfamiliar shoes and measurements were commonly conducted on a treadmill at a constant speed, which may not accurately reflect natural gait patterns in real-life settings. In contrast, this study utilized a compact and user-friendly IMS to collect gait data while participants wore their own familiar shoes. This approach allowed for accurate collection in a manner closer to daily life conditions, providing foundational data for assessing the effects of footwear under more realistic circumstances.

As a result, in terms of spatial parameters, the high-heeled group showed significantly shorter stride length, ~~lower~~ reduced maximum dorsiflexion angle, ~~lower~~ reduced maximum plantar flexion angle, and significantly ~~higher~~ greater toe-out angle compared to the sneaker group, with effect sizes ranging from moderate to high. Additionally, in spatiotemporal parameters, the high-heeled group exhibited significantly ~~lower~~ reduced walking speed and maximum speed during the swinging phase, with large effect sizes. However, no significant differences were observed between the groups in temporal parameters.

Regarding spatial parameters such as walking speed, stride length, and maximum swing speed, it is generally known that wearing high-heeled shoes reduces the base of support during walking, prompting individuals to adopt a safer and more stable gait pattern [~~28,29~~ 29,30]. ~~Additionally, to minimize increases in energy expenditure, they tend to decrease their walking speed and stride~~

~~length as a result.~~ Additionally, when wearing high heels, the reduced base of support during walking leads to increased muscle activity to maintain balance, which in turn tends to increase energy expenditure. To minimize this increase in energy expenditure, it has been shown that walking speed and stride length are reduced as a strategy [~~30,31~~ 31,32]. In addition to these stable and efficient walking strategies, previous studies have shown that wearing high heels brings the ground reaction force closer to the ankle joint, and the curved shape of the shoe sole causes the ankle to remain in a plantarflexed position [11]. These factors can shorten the length of the triceps surae muscle and Achilles tendons. Consequently, compared to sneakers or barefoot walking, ankle plantarflexion moments decrease, and the effect of the stretch-shortening cycle on the muscle-tendon complex is reduced [~~32,33~~ 33,34]. The stretch-shortening cycle is a process in which muscles and tendons quickly stretch and then shorten, store, and release elastic energy, and plays a crucial role in efficient force production [~~33~~ 34]. However, the shortened state of the muscle-tendon complex induced by wearing high heels is thought to impede this function, leading to decreased energy efficiency and a weaker push-off force. As a result, the calculated parameters were significantly different between the two groups.

Similarly, foot clearance and foot height were significantly ~~lower~~ reduced in the high-heeled group. As noted above, wearing high-heeled shoes is suggested to reduce the push-off force of the ankle. Additionally, previous studies investigating the role of the ankle in walking have shown that ankle

push-off contributes to up to 50% of the power needed for leg swinging during the terminal stance and early swing phases [~~34,35,36~~ 35,36,37]. ~~This reduction in ankle push-off may decrease the leg swing speed, which is essential for the double-pendulum effect during walking [37]. Consequently, a lack of sufficient knee flexion in the double-pendulum mechanism may cause the lower leg to remain relatively low, potentially resulting in reduced foot clearance.~~ This reduction in ankle push-off may decrease the leg swing speed, which is essential for the double-pendulum effect during walking [~~37~~, 38]. In the double-pendulum effect, an increased hip swing speed results in the relative lifting of the lower leg, consequently increasing foot clearance. This occurs as the hip and knee joints work in coordination, with the hip's movement influencing the lower leg's motion. Specifically, a rapid change in the hip angle causes the lower leg to rise, allowing the foot to clear obstacles before making contact with the ground. In the heel group, however, a reduction in leg swing speed leads to insufficient knee flexion in the double-pendulum mechanism, causing the lower leg to remain relatively low and potentially resulting in reduced foot clearance.

In fact, prior research comparing joint angles in the sagittal plane under different heel heights at a constant walking speed found that while hip flexion during the swing phase remained unaffected by heel height, the knee flexion angle significantly decreased as heel height increased [~~38,39,40~~ 39,40,41]. Moreover, a decrease in the foot clearance height can increase the risk of tripping over small obstacles or uneven surfaces while walking, potentially leading to falls [~~41~~ 42]. High-heeled

shoes also tilt the toes downward and elevate the heel, reducing the contact area of the foot with the ground, which may further destabilize balance. This combination of reduced foot clearance and a limited base of support likely exacerbates the risk of falls [14].

Thus, the high-heeled group showed significantly ~~lower~~ reduced stride length, ~~lower~~ reduced peak dorsiflexion angle of the foot, ~~lower~~ reduced peak plantar flexion angle of the foot, ~~lower~~ reduced walking speed, and swing phase maximum velocity, while the toe angle during the swing phase was significantly ~~higher~~ greater. A ~~higher~~ greater toe angle indicates a larger ankle abduction angle. Studies investigating the influence of heel height on the anterior-posterior joint moments of the ~~lower~~ reduced limb have shown that as heel height increases, knee internal rotation moments increase, potentially increasing the load on the medial compartment of the knee [9,~~42~~ 43]. Conversely, walking with toes pointing outward reduces knee internal rotation moments and decreases the load on the medial compartment [~~43~~ 44]. In this study, it is suggested that the high-heeled group adopted a walking strategy with toes pointing outward to reduce the load on the medial compartment of the knee. Additionally, since knee joint movements during stance and toe direction were not measured in this study, further experiments are necessary.

There are limitations in this study; the participants were limited to healthy adult women from the same university, which restricts the generalizability of our findings. To apply these results to other age groups, sexes, or individuals with specific health conditions, further studies with more diverse

participants are necessary. Second, the sample size in this study was limited to 17 participants, which poses constraints on statistical robustness and the power to detect effects. In particular, to adequately assess the impact of individual differences, it is essential to include a larger number of participants. Future studies should increase the sample size to enhance statistical robustness and improve the power to detect effects. Finally, we were unable to capture the movements of the hip and knee joints, making it challenging to fully assess how these joints affect gait mechanics, particularly balance and posture control. Specifically, it remains unclear to what extent high-heeled shoes stress on these joints under near-daily conditions and how this stress might increase the risk of falls or lead to long-term joint disorders. Future research should incorporate devices that measure the movements of these joints to provide a more comprehensive evaluation of the impact of different footwear types on gait and balance.

Conclusions

This study investigated the effects of high-heeled shoes on walking parameters in healthy adult women using the IMS. As a result, in terms of spatial parameters, the high-heeled group showed significantly shorter stride length, ~~lower~~ reduced maximum dorsiflexion angle, ~~lower~~ reduced maximum plantar flexion angle, and significantly ~~higher~~ greater toe-out angle compared to the sneaker group, with effect sizes ranging from moderate to high. Additionally, in spatiotemporal

parameters, the high-heeled group exhibited significantly ~~lower~~ reduced walking speed and maximum speed during the swinging phase, with large effect sizes. However, no significant differences were observed between the groups in temporal parameters. These findings suggest that wearing high-heeled shoes has a substantial impact on gait stability and efficiency, potentially increasing the risk of falls and musculoskeletal strain. Future studies should include a more diverse population, such as older adults or individuals with joint disorders, and incorporate motion capture systems or wearable sensors that can measure ~~lower~~ reduced limb joint dynamics. These additional studies would allow for a comprehensive understanding of how footwear affects not only the ankle but also hip and knee joint movements across a wider population. Because this study collected gait data under conditions similar to those of daily life, it provides data suitable for practical applications and may contribute to future research focused on evaluating gait in everyday scenarios.

Acknowledgments

We express our gratitude to all participants, instructors, and laboratory members for their cooperation and valuable input throughout this study. We also thank Editage (www.editage.com) for English editing.

References

1. Prince F, Corriveau H., Hebert R, Winter DA. Gait in elderly. Gait & Posture. 1997;5(2): 128-135. doi: 10.1016/S0966-6362(97)01118-1
2. Bai X, Kim GS, Dev RDO, Talib O, Xiao W, Cai H. Effect of Brisk Walking on Health-Related Physical Fitness Balance and Life Satisfaction Among the Elderly: A Systematic Review. Frontiers in Public Health. 2022;9. doi: https://doi.org/10.3389/fpubh.2021.829367
3. Menant JC, Steele JR, Menz HB, Munro BJ, Lord SR. Effects of walking surfaces and footwear on temporo-spatial gait parameters in young and older people. Gait & Posture. 2009;29(3):392-397. doi: https://doi.org/10.1016/j.gaitpost.2008.10.057
4. Barton CJ, Bonanno D, Menz HB. Development and evaluation of a tool for the assessment of footwear characteristics. Journal of Foot Ankle Research. 2009;2(10). doi: 10.1186/1757-1146-2-10
5. Shakoor N, Block JA. Walking Barefoot Decreases Loading on the Lower Extremity Joints in Knee Osteoarthritis. ARTHRITIS & RHEUMATISM. 2006;54(9):2923-2927. doi: 10.1002/art.22123
6. Keenan GS, Franz JR, Dicharry J, Della Croce U, Casey Kerrigan D. Lower limb joint kinetics in walking: The role of industry recommended footwear. Gait & Posture. 2011;33(3):350-355. doi: 10.1016/j.gaitpost.2010.09.019

7. Luximon Y, Cong Y, Luximon A, Zhang M. Effects of heel base size, walking speed, and slope angle on center of pressure trajectory and plantar pressure when wearing high-heeled shoes. Human Movement Science. 2015;41;307-319. doi: 10.1016/j.humov.2015.04.003

8. Zeng Z, Liu Y, Hu X, Li P, Wang L. Effects of high-heeled shoes on lower extremity biomechanics and balance in females: a systematic review and meta-analysis. BMC Public Health. 2023;23(726). doi: 10.1186/s12889-023-15641-8

9. Barkema DD, Derrick TR, Martin PE. Heel height affects lower extremity frontal plane joint moments during walking. Gait & Posture. 2012;35(3):483-488. doi: 10.1016/j.gaitpost.2011.11.013

10. Xavier MJ, Lambert B, Jenkins GP, McGwin Jr G. Epidemiology of High-Heel Shoe Injuries in U.S. Women: 2002 to 2012. ORIGINAL RESEARCH. 2015;54(4):615-619. doi: 10.1053/j.jfas.2015.04.008

11. Cronin NJ. The effects of high heeled shoes on female gait: A review. Journal of Electromyography and Kinesiology. 2014;24(2):258-263. doi:10.1016/j.jelekin.2014.01.004

12. Alkjaer T, Raffalt P, Petersen NC, Simonsen EB. Movement Behavior of High-Heeled Walking: How Does the Nervous System Control the Ankle Joint during an Unstable Walking Condition? PLoS ONE. 2012;7(5). doi: https://doi.org/10.1371/journal.pone.0037390

13. Falola JM, Koussihouèdé FEN, Falola SMD, Avossevou YG, Bio NI, Dansou HP. Effect of the Base of the Shoe Heel on Postural Stability During Walking in Women. American Journal of BioScience. 2015;3(5):167-170. doi: 10.11648/j.ajbio.20150305.11

14. Annoni I, Mapelli A, Sidequersky FV, Zago M, Sforza C. The effect of high-heeled shoes on overground gait kinematics in young healthy women. Sport Sciences for Health. 2014;10(2):149-157. doi: 10.1007/s11332-014-0191-z

15. Sloot LH, Van der Krogt MM, Harlaar J. Effects of adding a virtual reality environment to different modes of treadmill walking. Gait & Posture. 2014;39(3): 939-945. doi: https://doi.org/10.1016/j.gaitpost.2013.12.005

16. White SC, Yack HJ, Tucker CA, Lin HY. Comparison of vertical ground reaction forces during overground and treadmill walking. Medicine & Science in Sports & Exercise. 1998;30(10): 1537–1542. doi:10.1097/00005768-199810000-00011

17. Stolze H, Kuhtz-Buschbeck JP, Mondwurf C, Boczek-Funcke A, Jöhnk K, Deuschl G, Illert M. Gait analysis during treadmill and overground locomotion in children and adults. Electroencephalography and Clinical Neurophysiology. 1997;105(6): 490–497. doi:10.1016/s0924-980x(97)00055-6

18. Menz HB, Bonanno DR. Footwear comfort: a systematic search and narrative synthesis of the literature. Journal of Foot and Ankle Research. 2021;14:63. doi:https://doi.org/10.1186/s13047-021-00500-9

19. O'Rourke B, Walsh ME, Brophy R, Vallely S, Murphy N, Conroy B, et al. Does the shoe really fit? Characterising ill-fitting footwear among community-dwelling older adults attending geriatric services: an observational cross-sectional study. BMC Geriatrics. 2020; 20: 55. doi:https://doi.org/10.1186/s12877-020-1448-9

20. Aminian K, Renzakhanlou K, De Andres E, Fritsch C, Leyvraz PF, Robert P. Temporal feature estimation during walking using miniature accelerometers: an analysis of gait improvement after hip arthroplasty. Medical & Biological Engineering & Computing. 1999;37(6):686-691. doi: 10.1007/BF02513368

21. Jasiewicz Jan M, Allum John HJ, Middleton James W, Barriskill Andrew, Condie Peter, Pucell Brendan, et al. Gait event detection using linear accelerometers or angular velocity transducers in able-bodied and spinal-cord injured individuals. Gait & Posture. 2006;24(4):502-509. doi: https://doi.org/10.1016/j.gaitpost.2005.12.017

22. Anwary AR, Yu H, Vassallo M. Optimal Foot Location for Placing Wearable IMU Sensors and Automatic Feature Extraction for Gait Analysis. IEEE Sensors Journal. 2018;18(6):2555-2567. doi: 10.1109/JSEN.2017.2786587

23. Yamamoto A, Fujita K, Yamada E, Ibara T, Nihey F, Inai T, et al. Foot characteristics of the daily-life gait in postmenopausal females with distal radius fractures: a cross-sectional study. BMC Musculoskeletal Disorders. 2023;24(1). doi: 10.1186/s12891-023-06845-5

24. Chard A, Greene A, Hunt A, Vanwanseele B, Smith R. Effect of thong style flip-flops on children's barefoot walking and jogging kinematics. Journal of Foot and Ankle Research. 2013;6:8. doi:https://doi.org/10.1186/1757-1146-6-8

25. Fukushi K, Huang C, Wang Z, Kajitani H, Nihey F, Nakahara K. On-line algorithms of stride-parameter estimation for in-shoe motion-sensor system. IEEE Sensors Journal. 2022;22:9636–9648. doi:https://doi.org/10.1109/JSEN.2022.3164057

26. Huang C, Fukushi K, Wang Z, Nihey F, Kajitani H, Nakahara K. An algorithm for real time minimum toe clearance estimation from signal of in-shoe motion sensor. Annual International Conference of the IEEE Engineering in Medicine and Biology Society. 2021: 6775–6778. doi:https://doi.org/10.1109/EMBC46164.2021.9629875

27. Huang C, Fukushi K, Wang Z, Nihey F, Kajitani H, Nakahara K. Method for estimating temporal gait parameters concerning bilateral lower limbs of healthy subjects using a single in-shoe motion sensor through a gait event detection approach. Sensors (Basel). 2022;22(1):351. doi:https://doi.org/10.3390/S22010351

28. Cohen J. Statistical power analysis for the behavioral sciences. 2nd ed. Lawrence Erlbaum Associates; 1988. doi: https://doi.org/10.4324/9780203771587

29. Esenyel M, Walsh K, Walden JG, Gitter A. Kinetics of high-heeled gait. Journal of the American Podiatric Medical Association. 2003;93(1):27-32. doi: 10.7547/87507315-93-1-27

30. Lee CM, Jeong EH, Freivalds A. Biomechanical effects of wearing high-heeled shoes. International Journal of Industrial Ergonomics. 2001;28(6): 321–326. doi:https://doi.org/10.1016/S0169-8141(01)00038-5

31. Ebbeling CJ, Hamill J, Crussemeyer JA. Lower extremity mechanics and energy cost of walking in high-heeled shoes. Journal of Orthopaedic & Sports Physical Therapy. 1994;19(4): 190–196. doi:https://doi.org/10.2519/jospt.1994.19.4.190

32. Curran SA, Holliday JL, Watkeys L. Influence of high-heeled footwear and pre-fabricated orthoses on energy efficiency in ambulation. The Foot and Ankle Online Journal. 2010;3(3): 1–11. doi:https://doi.org/10.3827/faoj.2010.0303.0001

33. Komi PV. Stretch-shortening cycle: a powerful model to study normal and fatigued muscle. Journal of Biomechanics. 2000;33(10): 1197–1206. doi:https://doi.org/10.1016/S0021-9290(00)00064-6

34. Roberts TJ, Azizi E. Flexible mechanisms: the diverse roles of biological springs in vertebrate movement. Journal of Experimental Biology. 2011;214(3): 353–361. doi:https://doi.org/10.1242/jeb.038588

35. Fickey SN, Browne MG, Franz JR. Biomechanical effects of augmented ankle power output during human walking. Journal of Experimental Biology. 2018;221(22): jeb182113. doi:https://doi.org/10.1242/jeb.182113

36. Neptune RR, Clark DJ, Kautz SA. Modular control of human walking: A simulation study. Journal of Biomechanics. 2009;42(9): 1282–1287. doi:https://doi.org/10.1016/j.jbiomech.2009.03.009

37. Meinders M, Gitter A, Czerniecki JM. 1998; The role of ankle plantar flexor muscle work during walking. Scandinavian Journal of Rehabilitation Medicine. 30(1): 39–46. doi:https://doi.org/10.1080/003655098444309

38. Cinto F, Nacusse M, Haimovich H. Making a double pendulum walk: from compass- to whip-like gait. 2022 IEEE Biennial Congress of Argentina (ARGENCON). 2022;pp.1–8. doi:https://doi.org/10.1109/ARGENCON55245.2022.9939939

39. Snow RE, Williams KR. High Heeled Shoes: Their Effect on Center of Mass Position, Posture, Three-Dimensional Kinematics, Rearfoot Motion, and Ground Reaction Forces. Archives of Physical Medicine and Rehabilitation. 1994;75(5):568-576.

doi: https://doi.org/10.1016/S0003-9993(21)01622-1

40. Opila KA. Kinematics of high-heeled gait. Archives of Physical Medicine and Rehabilitation. 1990;71(5):304-309. doi: 10.7547/87507315-93-1-27

41. Gehlsen G, Braarz JS, Assmann N. Effects of heel height on knee rotation and gait. Human Movement Science. 1986;5(2):149-155. doi: https://doi.org/10.1016/0167-9457(86)90021-7

42. Dadashi F, Mariani B, Rochat S, Bula CJ, Santos-Eggiman, B, Aminian K. Gait and Foot Clearance Parameters Obtained Using Shoe-Worn Inertial Sensors in a Large-Population Sample of Older Adults. Sensors(Basel). 2014;14(1):443-457. doi: 10.3390/s140100443

43. Teichtahl AJ, Morrie ME, Wluka AE, Baker R, Wolfe R, Davis SR, et al. Foot rotation-a potential target to modify the knee adduction moment. Journal of Science and Medicine in Sport. 2006;9(1-2):67-71. doi: https://doi.org/10.1016/j.jsams.2006.03.011

44. Chang A, Hurwitz D, Dunlop D, Song J, Cahue S, Hayes K, et al. The relationship between toe-out angle during gait and progression of medial tibiofemoral osteoarthritis. Annals of the Rheumatic Diseases. 2007;66(10):1271-1275. doi: 10.1136/ard.2006.062927

Point-by-point response

We are very grateful to the editors for providing such valuable, thoughtful, and thorough comments. The comments and questions raised have prompted us to consider our data from new and interesting angles, the culmination of which we feel has greatly strengthened the work. Thank you for your time.

Editor’s comments:

[Comment #1]

Limited Contribution to the Literature

Your study’s findings largely align with existing research. Please clearly articulate what new insights your study provides.

[Author action #1]

Thank you for your thoughtful feedback. We believe we have already addressed our study's novelty and unique insights. We would be grateful if you could review that part, and we’re happy to further clarify or expand on any points if needed. Also, we re-evaluated the novelty of this study, which we believe primarily consists of the following three aspects:

1.Measurement under conditions close to daily life: Unlike other studies that use prescribed footwear, our study measured participants in their habitual shoes, collecting data that more accurately represents natural, everyday walking styles. Furthermore, many previous studies have conducted measurements on treadmills, which differ significantly from actual walking conditions. By performing measurements in real-life conditions, our study is able to provide practical insights that more realistically reflect individual walking patterns.

2.High-precision data collection of daily movement using IMS: Instead of traditional motion capture systems or treadmill-based experiments, we utilized the In-Shoe Motion Sensor (IMS) system, allowing measurements in an environment closer to everyday life. This enables a realistic evaluation of the impact of wearing high heels in typical environments outside the laboratory, offering valuable feedback applicable to real-world conditions.

3.Practical application perspective: The data collection method using IMS serves as foundational data that can be leveraged to evaluate fall risks in everyday walking and provide feedback for designing comfortable and safe footwear. This data can be applied in the future to develop more refined footwear suited for everyday walking and create effective gait training programs, adding further value to this study.

The specific parts demonstrating the novelty are as follows:

(The Introduction)

Research on the effects of high-heeled shoes on gait parameters has significantly progressed. However, many evaluations have been conducted using a treadmill with participants walking at a constant speed [14]. Treadmill walking occurs under flat, steady-speed conditions, which differs from everyday walking environments [15]. For example, because a treadmill maintains a fixed speed, participants cannot walk at their natural pace, potentially suppressing their typical gait patterns [16,17]. Additionally, on a treadmill, individuals often need to adjust their posture and gait mechanics to match the treadmill’s motion in order to maintain visual and balance cues, which is known to influence gait biomechanics [15]. In certain studies, participants wore unfamiliar shoes that may not accurately reflect their usual gait patterns in daily life [11,14]. Shoes that are unfamiliar or uncomfortable for an individual often do not fit well with the person’s foot shape or gait style, potentially affecting the gait biomechanics [18]. Specifically, when the shoe’s size or shape does not align with the foot, it may cause foot pain and fatigue, while also reducing stability and increasing the risk of falls during walking [19].

Therefore, conditions different from those in daily life may alter an individual's natural gait patterns and may not accurately reflect habitual walking [11].

As a more suitable evaluation method, Inertial Measurement Units (IMUs) can be used. IMUs can estimate three-dimensional movement from angular velocity and acceleration [20,21]. Due to their low cost, small size, and ease of use, they have been widely used to evaluate daily life walking in recent years [22]. By using the In-Shoe Motion Sensor system (IMS), which measures the acceleration and angular velocity of the feet using IMUs and calculates walking parameters, it is possible to obtain walking data comparable to daily life more accurately than conventional studies-[23].

Line numbers where the changed text appears: 81-102

(The introduction for Discussion)

This study aimed to evaluate the effect of high-heeled shoes on gait parameters in healthy adult women using an IMS system. In previous studies, participants often wore unfamiliar shoes and measurements were commonly conducted on a treadmill at a constant speed, which may not accurately reflect natural gait patterns in real-life settings. In contrast, this study utilized a compact and user-friendly IMS to collect gait data while participants wore their own familiar shoes. This approach allowed for

accurate collection in a manner closer to daily life conditions, providing foundational data for assessing the effects of footwear under more realistic circumstances.

Line numbers where the changed text appears: 221-227

(Conclusion)

Because this study collected gait data under conditions similar to those of daily life, it provides data suitable for practical applications and may contribute to future research focused on evaluating gait in everyday scenarios.

Line numbers where the changed text appears: 319-321

[Comment #2]

Sample Size and Power Issues

Seventeen participants are insufficient for drawing meaningful conclusions. Even though you state a power calculation, the rationale for choosing an effect size of 0.62 is unclear.

If increasing the sample size is not feasible, discuss this limitation more transparently in the manuscript.

[Author action #2]

Thank you for your valuable comments. We have revised the limitations section to clearly describe the constraints of the sample size and its impact on statistical power. Additionally, we have removed the mention of the sample size from the participants section. We have learned that if increasing the sample size is not feasible in future research, it is important to discuss this limitation more transparently, and we will keep this in mind moving forward. Thank you again for your insights. We appreciate your time in reviewing our revisions at your convenience.

The changed text is highlighted in yellow as follows:

There are limitations in this study; the participants were limited to healthy adult women from the same university, which restricts the generalizability of our findings. To apply these results to other age groups, sexes, or individuals with specific health conditions, further studies with more diverse participants are necessary. Second, the sample size in this study was limited to 17 participants, which poses constraints on statistical robustness and the power to detect effects. In particular, to adequately assess the impact of individual differences, it is essential to include a larger number of participants. Future studies should increase the sample size to enhance statistical robustness and improve the power to detect effects. Finally, we were unable to capture the movements of the hip and knee joints, making it challenging to fully assess how these joints affect gait

mechanics, particularly balance and posture control. Specifically, it remains unclear to what extent high-heeled shoes stress on these joints under near-daily conditions and how this stress might increase the risk of falls or lead to long-term joint disorders. Future research should incorporate devices that measure the movements of these joints to provide a more comprehensive evaluation of the impact of different footwear types on gait and balance.

Line numbers where the changed text appears: 293-297

[Comment #3]

Methodology Needs More Justification

Shoe Selection: Your definition of high-heeled shoes is vague. Standardizing heel height and shoe type would improve reproducibility.

Data Collection: The justification for measuring only three steps at a time is weak. Why not use a continuous gait analysis? Provide references that support your approach.

[Author action #3]

Thank you for your insightful comment. The definition of high-heeled shoes used in our study is based on previous research, which we have cited in the manuscript. We would

appreciate it if you could review those references for a better understanding of our approach.

Previous research;

Cronin NJ. The effects of high heeled shoes on female gait: A review. Journal of Electromyography and Kinesiology. 2014;24(2):258-263. doi:10.1016/j.jelekin.2014.01.004

About data collection, thank you for your comment. Previous studies have shown that sufficient reliability can be obtained with measurements of three steps, and this approach is particularly effective for evaluating gait parameters. Additionally, to ensure the reliability of the gait data, we perform measurements for three steps, and if the measurement is not successful, we repeat the process of measuring three steps to accurately collect the data.

Previous research;

1. Fukushi K, Huang C, Wang Z, Kajitani H, Nihey F, Nakahara K. On-line algorithms of stride-parameter estimation for in-shoe motion-sensor system. IEEE Sensors Journal. 2022;22:9636–9648. doi:https://doi.org/10.1109/JSEN.2022.3164057

2. Huang C, Fukushi K, Wang Z, Nihey F, Kajitani H, Nakahara K. An algorithm for real time minimum toe clearance estimation from signal of in-shoe motion sensor. Annual International Conference of the IEEE Engineering in Medicine and Biology Society. 2021: 6775–6778. doi:https://doi.org/10.1109/EMBC46164.2021.9629875
3. Huang C, Fukushi K, Wang Z, Nihey F, Kajitani H, Nakahara K. Method for estimating temporal gait parameters concerning bilateral lower limbs of healthy subjects using a single in-shoe motion sensor through a gait event detection approach. Sensors (Basel). 2022;22(1):351. doi:https://doi.org/10.3390/S22010351

[Comment #4]

Weak Biomechanical Explanation

The discussion lacks depth in explaining why high heels alter gait parameters. Consider integrating research on muscle activation, joint mechanics, and kinetic changes caused by high heels.

[Author action #4]

Thank you for your valuable feedback. We have revised the following sections to incorporate a more detailed biomechanical perspective. By adding supplementary

explanations to clarify key points, we have aimed to enhance the reader's understanding.

The changed text is highlighted in yellow as follows:

Additionally, when wearing high heels, the reduced base of support during walking leads to increased muscle activity to maintain balance, which in turn tends to increase energy expenditure. To minimize this increase in energy expenditure, it has been shown that walking speed and stride length are reduced as a strategy.

Line numbers where the changed text appears: 239-242

This reduction in ankle push-off may decrease the leg swing speed, which is essential for the double-pendulum effect during walking [37]. In the double-pendulum effect, an increased hip swing speed results in the relative lifting of the lower leg, consequently increasing foot clearance. This occurs as the hip and knee joints work in coordination, with the hip's movement influencing the lower leg's motion. Specifically, a rapid change in the hip angle causes the lower leg to rise, allowing the foot to clear obstacles before making contact with the ground. In the heel group, however, a reduction in leg swing speed leads to insufficient knee flexion in the double-pendulum mechanism,

causing the lower leg to remain relatively low and potentially resulting in reduced foot clearance.

Line numbers where the changed text appears: 261-269

[Comment #5]

Please describe the calibration protocol for this device; I assume that is completed before data collection. A couple of concerns with the use of IMUs is 1) data collection interference depending on laboratory conditions, eg, metal, etc., and 2) data drift. Were these issue that you were aware and could evaluate? Please clarify this.

[Author action #5]

Thank you for your insightful comments. As stated in previous studies, this device ensures the accuracy of data collection through real-time correction. By applying this real-time correction, continuous adjustments can be made, allowing for the elimination of experimental environmental errors as much as possible and enabling more precise data measurements. We appreciate your understanding. Additionally, since the experimental environment was designed to closely resemble daily life conditions, it was not possible to achieve complete control. However, as mentioned in prior research, the application of two algorithms—**the Three-dimensional Zero-Velocity-Update (3D**

ZUPT) algorithm and the On-line Stride Segmentation algorithm—ensures measurement accuracy. The on-line stride segmentation algorithm enables stable foot-flat detection using the foot angle, thereby achieving accurate stride segmentation. Meanwhile, the 3D ZUPT algorithm effectively corrects drift errors, allowing for precise stride parameter calculations within the IMS device. By working in conjunction, these algorithms compensate for measurement errors caused by environmental factors and data drift, ensuring highly reliable data collection. Additionally, I have incorporated further explanations into the revised manuscript. I would appreciate it if you could review it at your convenience.

This IMS maintains measurement accuracy by applying real-time correction through two algorithms: the Three-dimensional zero-velocity-update (3D ZUPT) algorithm and the On-line stride segmentation algorithm. The on-line stride segmentation algorithm enables stable foot-flat detection using the plantar angle, ensuring precise stride segmentation. Additionally, the 3D ZUPT algorithm effectively compensates for drift errors, allowing for accurate stride parameter computation within the IMS device [25].

The changed text is highlighted in yellow as follows: 140-145

[Comment #6]

please provide a scale with a range of effect size that indicate the strength of the effect size, i.e., its clinial significance.; reference this scale

[Author action #6]

Thank you for your suggestion. We have revised the manuscript to include a scale that indicates the strength of the effect size and its clinical significance, along with the appropriate reference. Please review the updated section at your convenience.

The changed text is highlighted in yellow as follows:

The effect size was evaluated using Cohen's d to assess the magnitude of the differences between groups, with thresholds defined as Small (0.2–0.49), Medium (0.5–0.79), and Large (≥ 0.8) [28].

Line numbers where the changed text appears: 193-195
