# Supplementary material for: The effects of high-heeled shoes on gait parameters in healthy adult women
Source: PLoS One. 2025 Jul 3;20(7):e0327250. doi: 10.1371/journal.pone.0327250 (PMC12225787; doi:10.1371/journal.pone.0327250)
Supplement: Attachment — Submitted filename: Point by Point Response ver2.docx [file pone.0327250.s004.docx]

Point-by-point response

We are very grateful to the editors for providing such valuable, thoughtful, and thorough comments. The comments and questions raised have prompted us to consider our data from new and interesting angles, the culmination of which we feel has greatly strengthened the work. Thank you for your time.

Editor’s comments:

[Comment #1]

Limited Contribution to the Literature
Your study’s findings largely align with existing research. Please clearly articulate what new insights your study provides.

[Author action #1]

Thank you for your thoughtful feedback. We believe we have already addressed our study's novelty and unique insights. We would be grateful if you could review that part, and we’re happy to further clarify or expand on any points if needed. Also, we re-evaluated the novelty of this study, which we believe primarily consists of the following three aspects:

1.Measurement under conditions close to daily life: Unlike other studies that use prescribed footwear, our study measured participants in their habitual shoes, collecting data that more accurately represents natural, everyday walking styles. Furthermore, many previous studies have conducted measurements on treadmills, which differ significantly from actual walking conditions. By performing measurements in real-life conditions, our study is able to provide practical insights that more realistically reflect individual walking patterns.

2.High-precision data collection of daily movement using IMS: Instead of traditional motion capture systems or treadmill-based experiments, we utilized the In-Shoe Motion Sensor (IMS) system, allowing measurements in an environment closer to everyday life. This enables a realistic evaluation of the impact of wearing high heels in typical environments outside the laboratory, offering valuable feedback applicable to real-world conditions.

3.Practical application perspective: The data collection method using IMS serves as foundational data that can be leveraged to evaluate fall risks in everyday walking and provide feedback for designing comfortable and safe footwear. This data can be applied in the future to develop more refined footwear suited for everyday walking and create effective gait training programs, adding further value to this study.

The specific parts demonstrating the novelty are as follows:

(The Introduction)

Research on the effects of high-heeled shoes on gait parameters has significantly progressed. However, many evaluations have been conducted using a treadmill with participants walking at a constant speed [14]. Treadmill walking occurs under flat, steady-speed conditions, which differs from everyday walking environments [15]. For example, because a treadmill maintains a fixed speed, participants cannot walk at their natural pace, potentially suppressing their typical gait patterns [16,17]. Additionally, on a treadmill, individuals often need to adjust their posture and gait mechanics to match the treadmill’s motion in order to maintain visual and balance cues, which is known to influence gait biomechanics [15]. In certain studies, participants wore unfamiliar shoes that may not accurately reflect their usual gait patterns in daily life [11,14]. Shoes that are unfamiliar or uncomfortable for an individual often do not fit well with the person’s foot shape or gait style, potentially affecting the gait biomechanics [18]. Specifically, when the shoe’s size or shape does not align with the foot, it may cause foot pain and fatigue, while also reducing stability and increasing the risk of falls during walking [19]. Therefore, conditions different from those in daily life may alter an individual’s natural gait patterns and may not accurately reflect habitual walking [11].

As a more suitable evaluation method, Inertial Measurement Units (IMUs) can be used. IMUs can estimate three-dimensional movement from angular velocity and acceleration [20,21]. Due to their low cost, small size, and ease of use, they have been widely used to evaluate daily life walking in recent years [22]. By using the In-Shoe Motion Sensor system (IMS), which measures the acceleration and angular velocity of the feet using IMUs and calculates walking parameters, it is possible to obtain walking data comparable to daily life more accurately than conventional studies [23].

Line numbers where the changed text appears: 81-102

(The introduction for Discussion)

This study aimed to evaluate the effect of high-heeled shoes on gait parameters in healthy adult women using an IMS system. In previous studies, participants often wore unfamiliar shoes and measurements were commonly conducted on a treadmill at a constant speed, which may not accurately reflect natural gait patterns in real-life settings. In contrast, this study utilized a compact and user-friendly IMS to collect gait data while participants wore their own familiar shoes. This approach allowed for accurate collection in a manner closer to daily life conditions, providing foundational data for assessing the effects of footwear under more realistic circumstances.

Line numbers where the changed text appears: 221-227

(Conclusion)

Because this study collected gait data under conditions similar to those of daily life, it provides data suitable for practical applications and may contribute to future research focused on evaluating gait in everyday scenarios.

Line numbers where the changed text appears: 319-321

[Comment #2]

Sample Size and Power Issues
Seventeen participants are insufficient for drawing meaningful conclusions. Even though you state a power calculation, the rationale for choosing an effect size of 0.62 is unclear.
If increasing the sample size is not feasible, discuss this limitation more transparently in the manuscript.

[Author action #2]

Thank you for your valuable comments. We have revised the limitations section to clearly describe the constraints of the sample size and its impact on statistical power. Additionally, we have removed the mention of the sample size from the participants section. We have learned that if increasing the sample size is not feasible in future research, it is important to discuss this limitation more transparently, and we will keep this in mind moving forward. Thank you again for your insights. We appreciate your time in reviewing our revisions at your convenience.

The changed text is highlighted in yellow as follows:

There are limitations in this study; the participants were limited to healthy adult women from the same university, which restricts the generalizability of our findings. To apply these results to other age groups, sexes, or individuals with specific health conditions, further studies with more diverse participants are necessary. Second, the sample size in this study was limited to 17 participants, which poses constraints on statistical robustness and the power to detect effects. In particular, to adequately assess the impact of individual differences, it is essential to include a larger number of participants. Future studies should increase the sample size to enhance statistical robustness and improve the power to detect effects. Finally, we were unable to capture the movements of the hip and knee joints, making it challenging to fully assess how these joints affect gait mechanics, particularly balance and posture control. Specifically, it remains unclear to what extent high-heeled shoes stress on these joints under near-daily conditions and how this stress might increase the risk of falls or lead to long-term joint disorders. Future research should incorporate devices that measure the movements of these joints to provide a more comprehensive evaluation of the impact of different footwear types on gait and balance.

Line numbers where the changed text appears: 293-297

[Comment #3]
Methodology Needs More Justification
Shoe Selection: Your definition of high-heeled shoes is vague. Standardizing heel height and shoe type would improve reproducibility.
Data Collection: The justification for measuring only three steps at a time is weak. Why not use a continuous gait analysis? Provide references that support your approach.
[Author action #3]

Thank you for your insightful comment. The definition of high-heeled shoes used in our study is based on previous research, which we have cited in the manuscript. We would appreciate it if you could review those references for a better understanding of our approach.

Previous research;

Cronin NJ. The effects of high heeled shoes on female gait: A review. Journal of Electromyography and Kinesiology. 2014;24(2):258-263. doi:[10.1016/j.jelekin.2014.01.004](https://doi.org/10.1016/j.jelekin.2014.01.004)

About data collection, thank you for your comment. Previous studies have shown that sufficient reliability can be obtained with measurements of three steps, and this approach is particularly effective for evaluating gait parameters. Additionally, to ensure the reliability of the gait data, we perform measurements for three steps, and if the measurement is not successful, we repeat the process of measuring three steps to accurately collect the data.

Previous research;

1. Fukushi K, Huang C, Wang Z, Kajitani H, Nihey F, Nakahara K. On-line algorithms of stride-parameter estimation for in-shoe motion-sensor system. IEEE Sensors Journal. 2022;22:9636–9648. doi:<https://doi.org/10.1109/JSEN.2022.3164057>
2. Huang C, Fukushi K, Wang Z, Nihey F, Kajitani H, Nakahara K. An algorithm for real time minimum toe clearance estimation from signal of in-shoe motion sensor. Annual International Conference of the IEEE Engineering in Medicine and Biology Society. 2021: 6775–6778. doi:<https://doi.org/10.1109/EMBC46164.2021.9629875>
3. Huang C, Fukushi K, Wang Z, Nihey F, Kajitani H, Nakahara K. Method for estimating temporal gait parameters concerning bilateral lower limbs of healthy subjects using a single in-shoe motion sensor through a gait event detection approach. Sensors (Basel). 2022;22(1):351. doi:<https://doi.org/10.3390/S22010351>

[Comment #4]

Weak Biomechanical Explanation

The discussion lacks depth in explaining why high heels alter gait parameters. Consider integrating research on muscle activation, joint mechanics, and kinetic changes caused by high heels.

[Author action #4]

Thank you for your valuable feedback. We have revised the following sections to incorporate a more detailed biomechanical perspective. By adding supplementary explanations to clarify key points, we have aimed to enhance the reader’s understanding.

The changed text is highlighted in yellow as follows:

Additionally, when wearing high heels, the reduced base of support during walking leads to increased muscle activity to maintain balance, which in turn tends to increase energy expenditure. To minimize this increase in energy expenditure, it has been shown that walking speed and stride length are reduced as a strategy.

Line numbers where the changed text appears: 239-242

This reduction in ankle push-off may decrease the leg swing speed, which is essential for the double-pendulum effect during walking [37]. In the double-pendulum effect, an increased hip swing speed results in the relative lifting of the lower leg, consequently increasing foot clearance. This occurs as the hip and knee joints work in coordination, with the hip’s movement influencing the lower leg’s motion. Specifically, a rapid change in the hip angle causes the lower leg to rise, allowing the foot to clear obstacles before making contact with the ground. In the heel group, however, a reduction in leg swing speed leads to insufficient knee flexion in the double-pendulum mechanism, causing the lower leg to remain relatively low and potentially resulting in reduced foot clearance.

Line numbers where the changed text appears: 261-269
[Comment #5]

Please describe the calibration protocol for this device; I assume that is completed before data collection. A couple of concerns with the use of IMUs is 1) data collection interference depending on laboratory conditions, eg, metal, etc., and 2) data drift. Were these issue that you were aware and could evaluate? Please clarify this.

[Author action #5]

Thank you for your insightful comments. As stated in previous studies, this device ensures the accuracy of data collection through real-time correction. By applying this real-time correction, continuous adjustments can be made, allowing for the elimination of experimental environmental errors as much as possible and enabling more precise data measurements. We appreciate your understanding. Additionally, since the experimental environment was designed to closely resemble daily life conditions, it was not possible to achieve complete control. However, as mentioned in prior research, the application of two algorithms—**the Three-dimensional Zero-Velocity-Update (3D ZUPT) algorithm and the On-line Stride Segmentation algorithm**—ensures measurement accuracy. The on-line stride segmentation algorithm enables stable foot-flat detection using the foot angle, thereby achieving accurate stride segmentation. Meanwhile, the 3D ZUPT algorithm effectively corrects drift errors, allowing for precise stride parameter calculations within the IMS device. By working in conjunction, these algorithms compensate for measurement errors caused by environmental factors and data drift, ensuring highly reliable data collection. Additionally, I have incorporated further explanations into the revised manuscript. I would appreciate it if you could review it at your convenience.

This IMS maintains measurement accuracy by applying real-time correction through two algorithms: the Three-dimensional zero-velocity-update (3D ZUPT) algorithm and the On-line stride segmentation algorithm. The on-line stride segmentation algorithm enables stable foot-flat detection using the plantar angle, ensuring precise stride segmentation. Additionally, the 3D ZUPT algorithm effectively compensates for drift errors, allowing for accurate stride parameter computation within the IMS device [25].
The changed text is highlighted in yellow as follows: 140-145

[Comment #6]

please provide a scale with a range of effect size that indicate the strength of the effect size, i.e., its clinial significance.; reference this scale

[Author action #6]

Thank you for your suggestion. We have revised the manuscript to include a scale that indicates the strength of the effect size and its clinical significance, along with the appropriate reference. Please review the updated section at your convenience.

The changed text is highlighted in yellow as follows:

The effect size was evaluated using Cohen’s d to assess the magnitude of the differences between groups, with thresholds defined as Small (0.2–0.49), Medium (0.5–0.79), and Large (≥ 0.8) [28].

Line numbers where the changed text appears: 193-195
