# Supplementary material for: The effects of high-heeled shoes on gait parameters in healthy adult women
Source: PLoS One. 2025 Jul 3;20(7):e0327250. doi: 10.1371/journal.pone.0327250 (PMC12225787; doi:10.1371/journal.pone.0327250)
Supplement: Attachment — Submitted filename: Point by point response_ver4.docx [file pone.0327250.s006.docx]

Point-by-point response

We are very grateful to the editors for providing such valuable, thoughtful, and thorough comments. The comments and questions raised have prompted us to consider our data from new and interesting angles, the culmination of which we feel has greatly strengthened the work. This manuscript has not been published or presented elsewhere and is not under consideration by any other journal. All study participants provided informed consent, and the study design was approved by the appropriate ethics review board. We have read and understood your journal’s policies and believe that neither the manuscript nor the study violates any of these policies.

Thank you for your time.

[Comment #1]

Theoretical Context and Terminology:
The revised introduction and discussion more clearly articulate the novelty of the study. However, to improve cohesion, I recommend defining terms such as “habitual shoes,” “natural gait,” and “real-life conditions” early on and using them consistently throughout the manuscript.

[Author action #1]

Thank you very much for your valuable feedback. As you rightly pointed out, clearly defining key terms at the beginning of the manuscript is essential for enhancing the clarity and consistency of the overall text. Therefore, we have added definitions for the terms “habitual shoes,” “natural gait,” and “real-life conditions” in the Introduction section and ensured that these terms are used consistently throughout the manuscript. We believe these revisions have improved the theoretical foundation and overall readability of the paper. The revised sections are as follows:

In this study, real-life conditions refer to natural walking situations that closely resemble daily life, without the use of treadmills or experimental footwear. Natural gait refers to an individual’s inherent walking pattern under conditions without external constraints or artificial instructions, and without any intentional modification of their gait.

Line numbers where the changed text appears: 184-187

Accordingly, the habitual shoes used in this study refer to the footwear that the participants considered to be the ones they wore most frequently during daily activities, such as commuting and shopping.

Line numbers where the changed text appears: 161-165

[Comment #2]

Methodology Clarity:
While your explanation for using a three-step gait measurement approach is now supported by literature, the rationale could benefit from slightly deeper elaboration. Briefly explaining why this method was chosen over continuous gait analysis (e.g., sensor limitations, data precision, ecological validity) would enhance transparency.

[Author action #2]

Thank you very much for your valuable feedback. We would like to respond to this matter from the perspective of *data precision*. In order to ensure the reliability of the three-step gait measurement in this study, we compared the RMSE values reported in a previous study (Matsumura et al., 2022, IEEE) using the same three-step gait approach with the intergroup differences observed in our research. As a result, the intergroup differences exceeded the RMSE in most variables, suggesting that the measurement method used in our study possesses a certain degree of reliability. We believe that this revision is the most appropriate way to demonstrate the reliability of our measurement approach among the options we considered. We hope this measurement method will be adopted by more researchers in future studies. We kindly ask for your understanding and confirmation on this point. The revised sections are as follows:

In a previous study [25], the RMSE for stride length and walking speed based on measurements taken over three steps was reported as 0.042 and 0.071, respectively. In contrast, the differences between the HG and SG in the present study were 0.1346 for stride length and 0.47 for walking speed, both of which substantially exceeded the reported RMSE values. Therefore, these differences are considered to exceed the range of measurement error and can be interpreted as practically meaningful and statistically significant group differences.

Line numbers where the changed text appears: 246-251

In a previous study [25], the RMSE for foot height was reported to be 0.017. In contrast, the difference between the HG and SG observed in this study was 0.0464, which substantially exceeds the RMSE. Therefore, the difference in foot clearance can be considered to exceed the margin of measurement error and represents a practically and statistically significant intergroup difference.

Line numbers where the changed text appears: 270-273

In a previous study [25], the RMSE for toe angle during the swing phase was reported to be 2.071. In contrast, the difference between the HG and SG observed in this study was 1.91, which is approximately the same as the RMSE. Therefore, it is possible that the observed difference in toe angle may not be entirely free from the influence of measurement error.

Line numbers where the changed text appears: 306-310

Finally, for the toe angle, the difference between groups was comparable to the RMSE, making it difficult to conclusively determine that the observed difference was statistically meaningful. Therefore, future studies should adopt more precise measurement techniques to verify the reliability of this difference.

Line numbers where the changed text appears: 324-327

[Comment #3]

Standardization of High-Heeled Shoe Definition:
Although you referenced existing literature to define high-heeled shoes, consider including a more specific numerical range (e.g., heel height in centimeters) within your methods section to improve reproducibility for future studies.
[Author action #3]

Thank you very much for your valuable feedback. In response to your comment, we have revised the Methods section to explicitly state the heel height in centimeters. In this study, we defined high-heeled shoes as those with a heel height of 3 cm or more. This definition was based on previous studies that examined gait characteristics in young adult females (e.g., Lee, 2014; Park, 2009), in which shoes with heel heights of 3 cm or greater were used. By specifying a numerical range in centimeters, we aimed to enhance the reproducibility and clarity of the operational definition of high-heeled shoes. We appreciate your thoughtful review and hope the revision addresses your concern. The changed text is highlighted in yellow as follows:

Based on previous studies that used shoes with a heel height of 3 cm or more as high-heeled footwear, the present study defined high-heeled shoes as those with a heel height of 3 cm or greater.

Line numbers where the changed text appears: 161-165

(Previous research)

Lee CR. The Effects of Lower Extremity Angle According to Heel-height Changes in Young Ladies in Their 20s during Gait. *Journal of Physical Therapy Science.* 2014;26(7):1055–1058. doi:10.1589/jpts.26.1055

Park JJ. A Comparative Analysis on Changes of Foot Pressure by Shoe Heel Height during Walking. *Korean Journal of Applied Biomechanics.* 2009;19(4):771–778. doi:10.5103/KJSB.2009.19.4.771

[Comment #4]

Effect Size Interpretation:
The inclusion of Cohen’s d scale is appropriate and appreciated. However, to enhance interpretability for clinical or applied readers, you might briefly comment on how the observed effect sizes relate to meaningful changes in gait mechanics (e.g., stride length, foot clearance).

[Author action #4]
Thank you for your insightful comment regarding the interpretation of effect sizes. In response, we have added a brief explanation of how the observed effect sizes relate to meaningful changes in gait mechanics, such as stride length and foot clearance. The changed text is highlighted in yellow as follows:

Regarding spatial parameters such as walking speed, stride length, and swing speed, all were significantly reduced in the high-heeled group, with large to moderate effect sizes: Cohen’s d = -1.09 for stride length and walking speed, and Cohen’s d = -0.75 for swing speed. These results strongly suggest that wearing high-heeled shoes has a substantial impact on propulsion and walking efficiency. Generally, wearing high heels narrows the base of support during walking, prompting individuals to adopt a safer and more stable gait pattern [31,32].

Line numbers where the changed text appears: 240-246

A very large effect size for foot clearance (Cohen’s d = -1.09) and a large effect size for foot height (Cohen’s d = -0.90) strongly suggest that foot clearance was more reduced in the high-heeled group.

Line numbers where the changed text appears: 267-269

This change demonstrated a moderate effect size (Cohen’s d = 0.54), suggesting that individuals wearing high heels may adjust foot positioning to enhance stability.

Line numbers where the changed text appears: 296-298

[Comment #5]

Calibration Protocol and Sensor Accuracy:
Your explanation of the 3D ZUPT and stride segmentation algorithms is technically sound. One suggestion would be to clarify explicitly whether a manual calibration procedure was performed prior to data collection, as this is a common step in IMU-based research.

[Author action #5]

Thank you for your comment regarding the calibration protocol and sensor accuracy. In this study, we did not perform manual calibration, as we relied on the ZUPT and stride segmentation algorithms for accurate measurements. We have now clarified this point in the revised manuscript. The changed text is highlighted in yellow as follows:

Due to the implementation of these algorithms, no manual calibration of the IMU was conducted in this study.

Line numbers where the changed text appears: 141-142
